# Supplementary figures and images for: Sepsis-associated pathways segregate cancer groups
Source: BMC Cancer. 2020 Apr 15;20:309. doi: 10.1186/s12885-020-06774-9 (PMC7160985; doi:10.1186/s12885-020-06774-9)

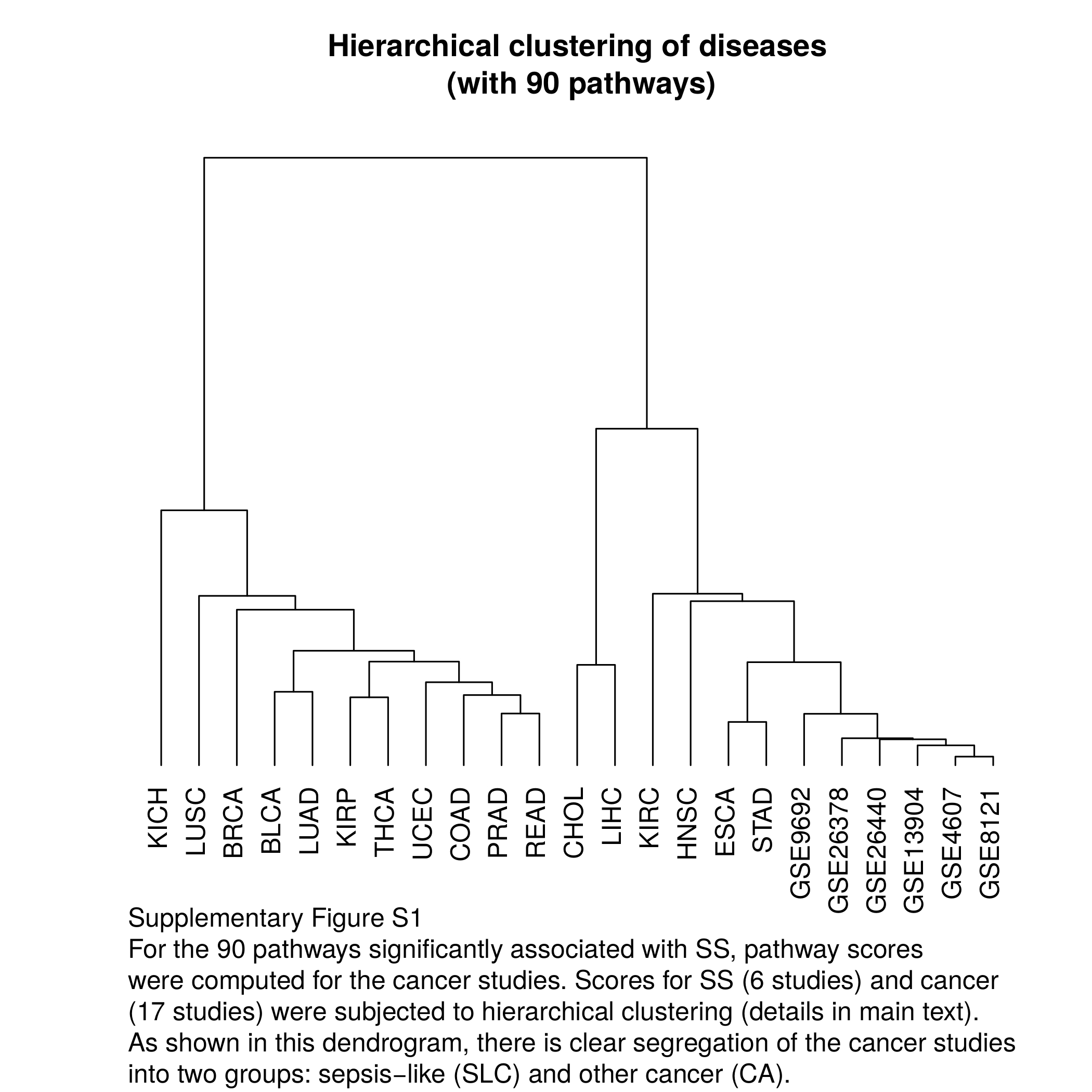

Supplement: Supplementary file 1 — Additional file 1: Figure S1. For the 90 pathways significantly associated with SS, pathway scores were computed for the cancer studies. Scores for SS (6 studies) and cancer (17 studies) were subjected to hierarchical clustering (details in main text). As shown in this dendrogram, there is clear segregation of the cancer studies into two groups: sepsis−like (SLC) and other cancer (CA). [file 12885_2020_6774_MOESM1_ESM.jpg]

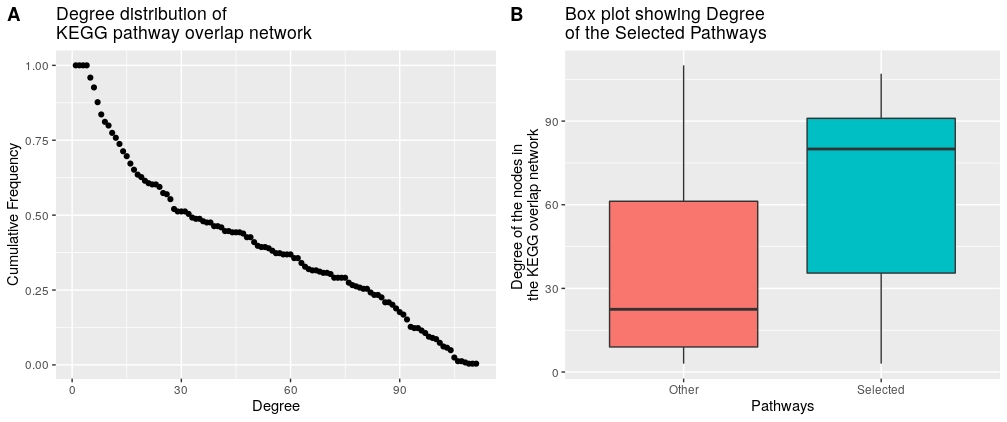

Supplement: Supplementary file 3 — Additional file 3: Figure S3. (A) This is the degree distribution of the network generated by overlapping KEGG pathways. This shows that there are many nodes with very low connectivity but a few nodes with very high connectivity. This is consistent with the scale-free property of biological networks. (B) Box plot showing comparison of degree between two groups of nodes in this network - selected 66 and others. The higher value of the selected 66 nodes suggests that these are highly overlapping pathways forming a functional core of the human genome. [file 12885_2020_6774_MOESM3_ESM.jpeg]
