# Supplementary material for: Sepsis-associated pathways segregate cancer groups
Source: BMC Cancer. 2020 Apr 15;20:309. doi: 10.1186/s12885-020-06774-9 (PMC7160985; doi:10.1186/s12885-020-06774-9)
Supplement: Supplementary file 2 — Additional file 2: Figure S2. Gene level heat map of each of the selected 66 pathways. LFC for each gene was calculated from disease and normal samples (disease-normal) for all studies. For each disease type (CA, SLC or SS), median LFC was calculated across the studies in that disease type. Combined heat map was generated based on the pathway genes and the three disease groups. We considered only those genes which have similar LFC directionality in SLC and SS. Red colour represents up-regulation in disease compared to control, while green colour represents down-regulation. Pathway names with KEGG identifiers are shown on the top, gene symbols and names are row-labels of heat map. The major class (to which the pathway belongs) is shown at the bottom. [file 12885_2020_6774_MOESM2_ESM.pdf]

Gene−level heatmap for 66 pathways

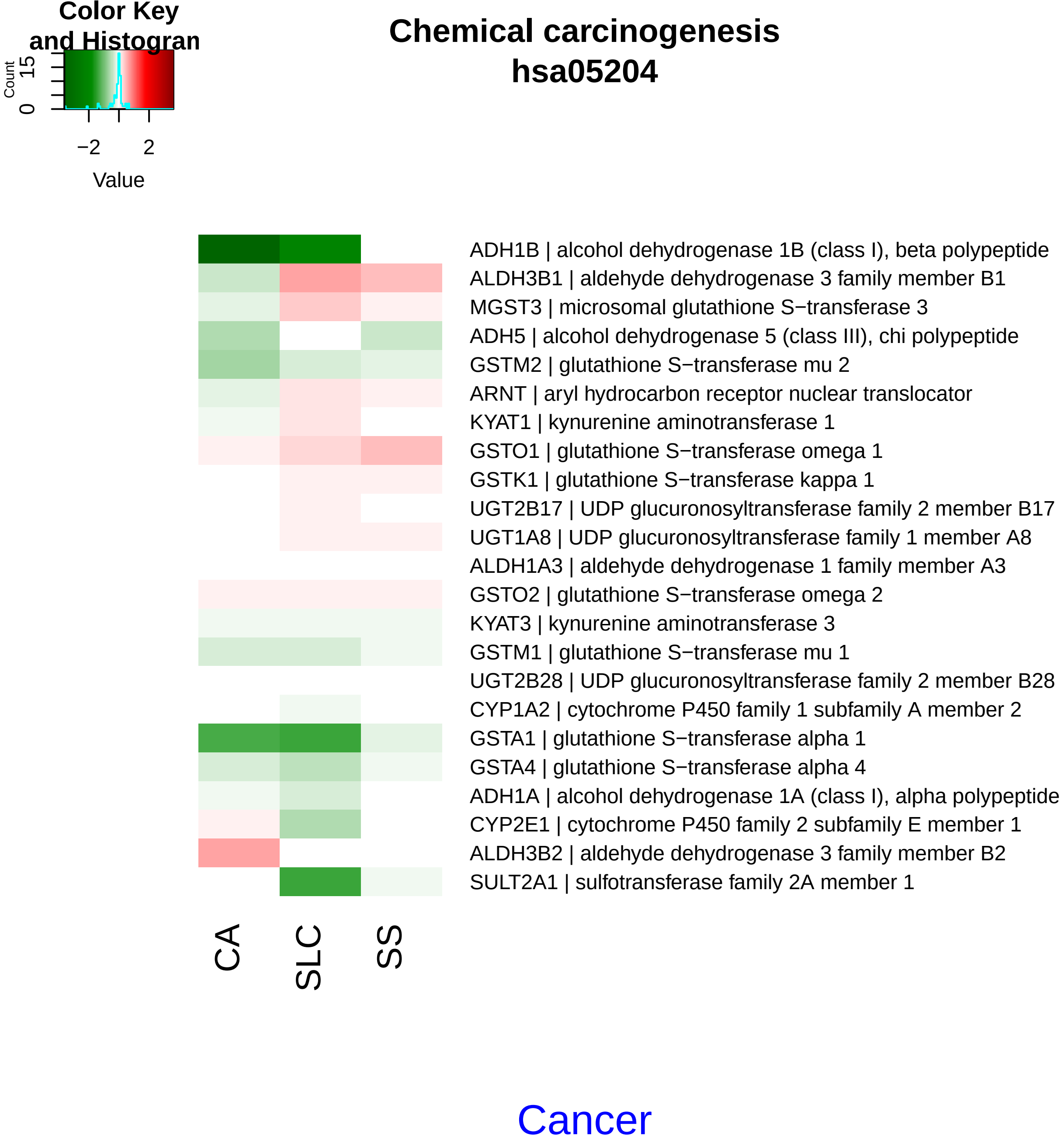
Color Key
and Histogran
Count
0
15
−2
2
Value
Chemical carcinogenesis
hsa05204
ADH1B | alcohol dehydrogenase 1B (class I), beta polypeptide
ALDH3B1 | aldehyde dehydrogenase 3 family member B1
MGST3 | microsomal glutathione S−transferase 3
ADH5 | alcohol dehydrogenase 5 (class III), chi polypeptide
GSTM2 | glutathione S−transferase mu 2
ARNT | aryl hydrocarbon receptor nuclear translocator
KYAT1 | kynurenine aminotransferase 1
GSTO1 | glutathione S−transferase omega 1
GSTK1 | glutathione S−transferase kappa 1
UGT2B17 | UDP glucuronosyltransferase family 2 member B17
UGT1A8 | UDP glucuronosyltransferase family 1 member A8
ALDH1A3 | aldehyde dehydrogenase 1 family member A3
GSTO2 | glutathione S−transferase omega 2
KYAT3 | kynurenine aminotransferase 3
GSTM1 | glutathione S−transferase mu 1
UGT2B28 | UDP glucuronosyltransferase family 2 member B28
CYP1A2 | cytochrome P450 family 1 subfamily A member 2
GSTA1 | glutathione S−transferase alpha 1
GSTA4 | glutathione S−transferase alpha 4
ADH1A | alcohol dehydrogenase 1A (class I), alpha polypeptide
CYP2E1 | cytochrome P450 family 2 subfamily E member 1
ALDH3B2 | aldehyde dehydrogenase 3 family member B2
SULT2A1 | sulfotransferase family 2A member 1
CA
SLC
SS
Cancer

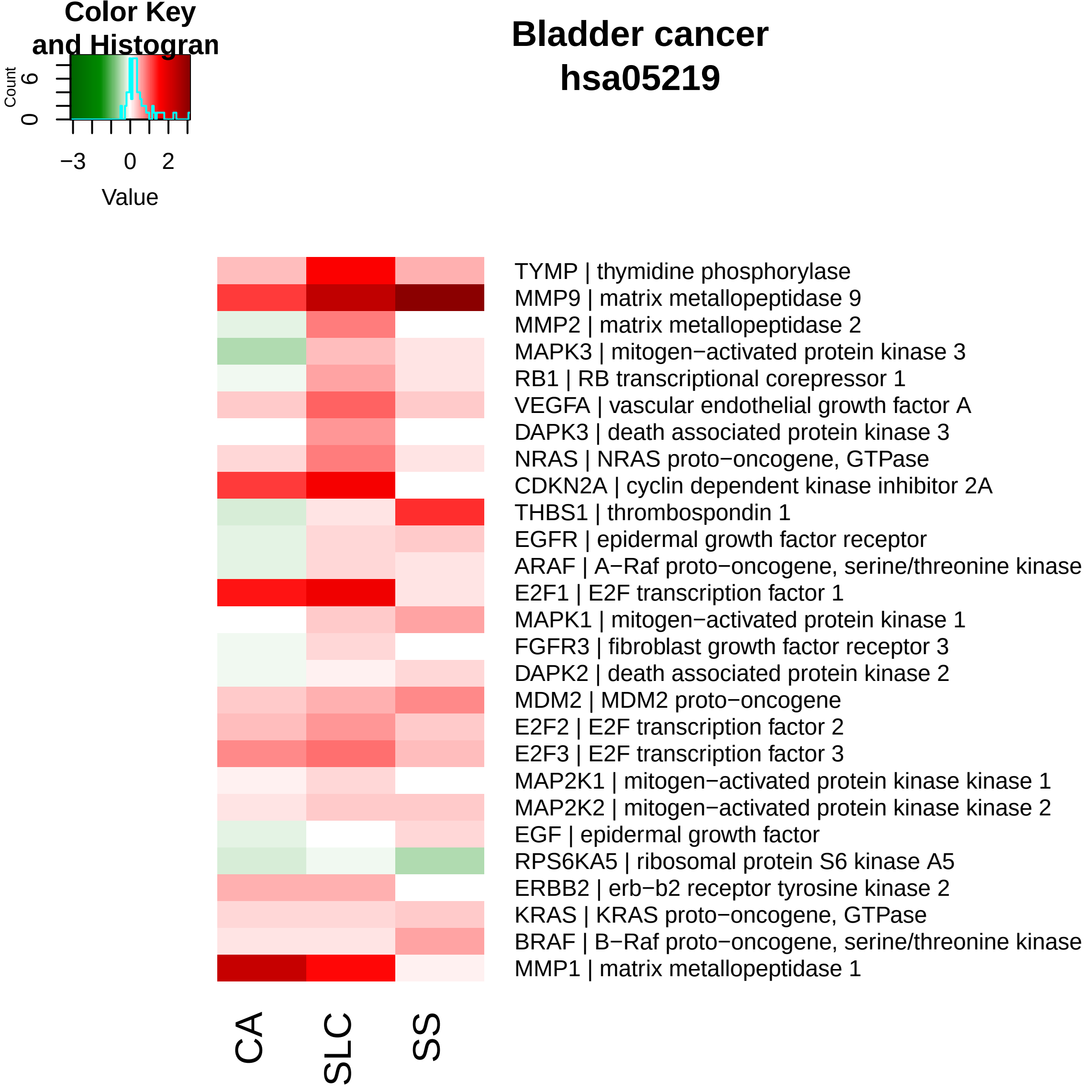

Bladder cancer
hsa05219
Color Key
and Histogram
Count
0
6
−3
0
2
Value
TYMP | thymidine phosphorylase
MMP9 | matrix metallopeptidase 9
MMP2 | matrix metallopeptidase 2
MAPK3 | mitogen−activated protein kinase 3
RB1 | RB transcriptional corepressor 1
VEGFA | vascular endothelial growth factor A
DAPK3 | death associated protein kinase 3
NRAS | NRAS proto−oncogene, GTPase
CDKN2A | cyclin dependent kinase inhibitor 2A
THBS1 | thrombospondin 1
EGFR | epidermal growth factor receptor
ARAF | A−Raf proto−oncogene, serine/threonine kinase
E2F1 | E2F transcription factor 1
MAPK1 | mitogen−activated protein kinase 1
FGFR3 | fibroblast growth factor receptor 3
DAPK2 | death associated protein kinase 2
MDM2 | MDM2 proto−oncogene
E2F2 | E2F transcription factor 2
E2F3 | E2F transcription factor 3
MAP2K1 | mitogen−activated protein kinase kinase 1
MAP2K2 | mitogen−activated protein kinase kinase 2
EGF | epidermal growth factor
RPS6KA5 | ribosomal protein S6 kinase A5
ERBB2 | erb−b2 receptor tyrosine kinase 2
KRAS | KRAS proto−oncogene, GTPase
BRAF | B−Raf proto−oncogene, serine/threonine kinase
MMP1 | matrix metallopeptidase 1
CA
SLC
SS

Cancer

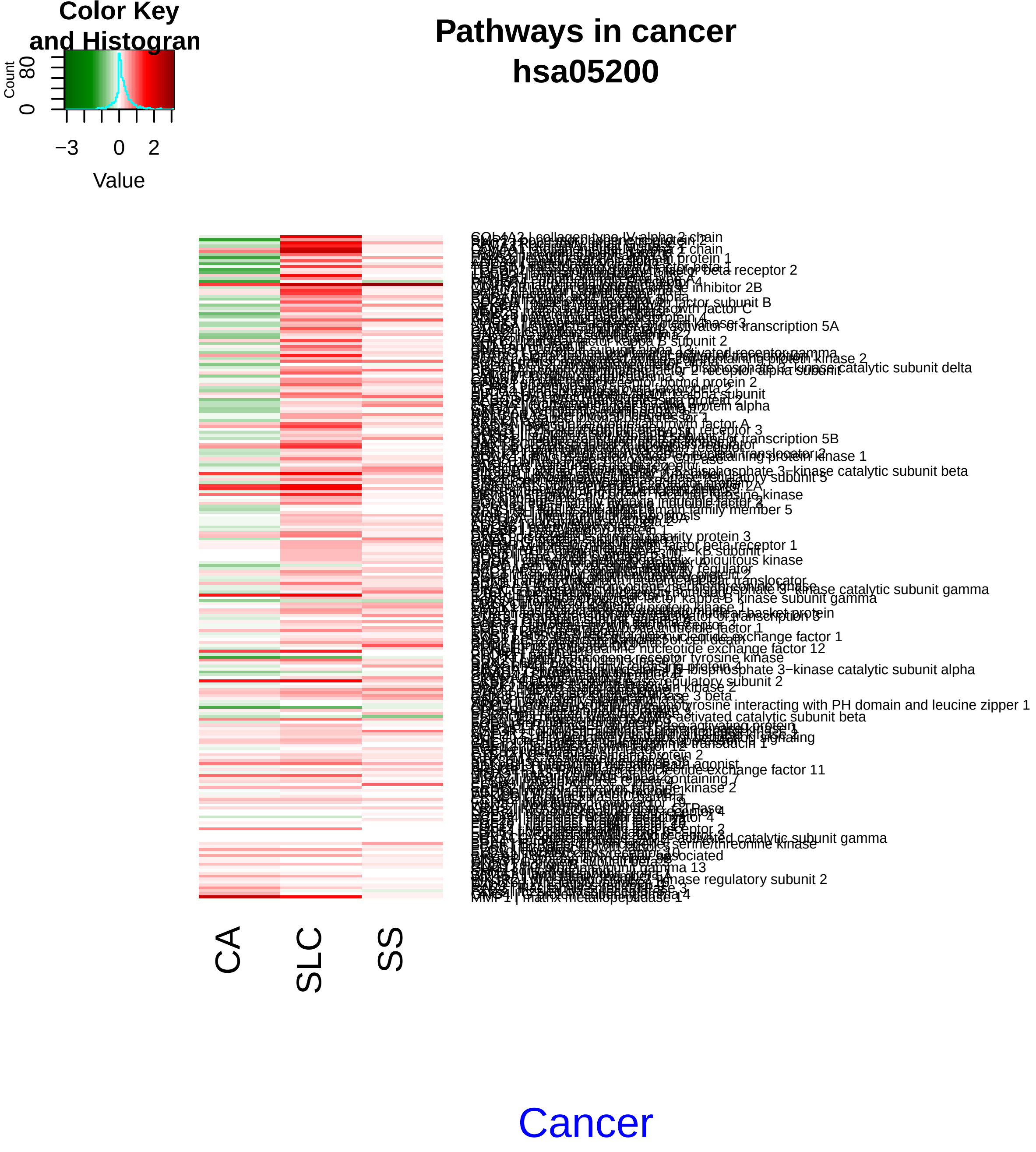

Color Key and Histogran
Count
0
80
−3
0
2
Value
Pathways in cancer
hsa05200
CA
SLC
SS
Cancer

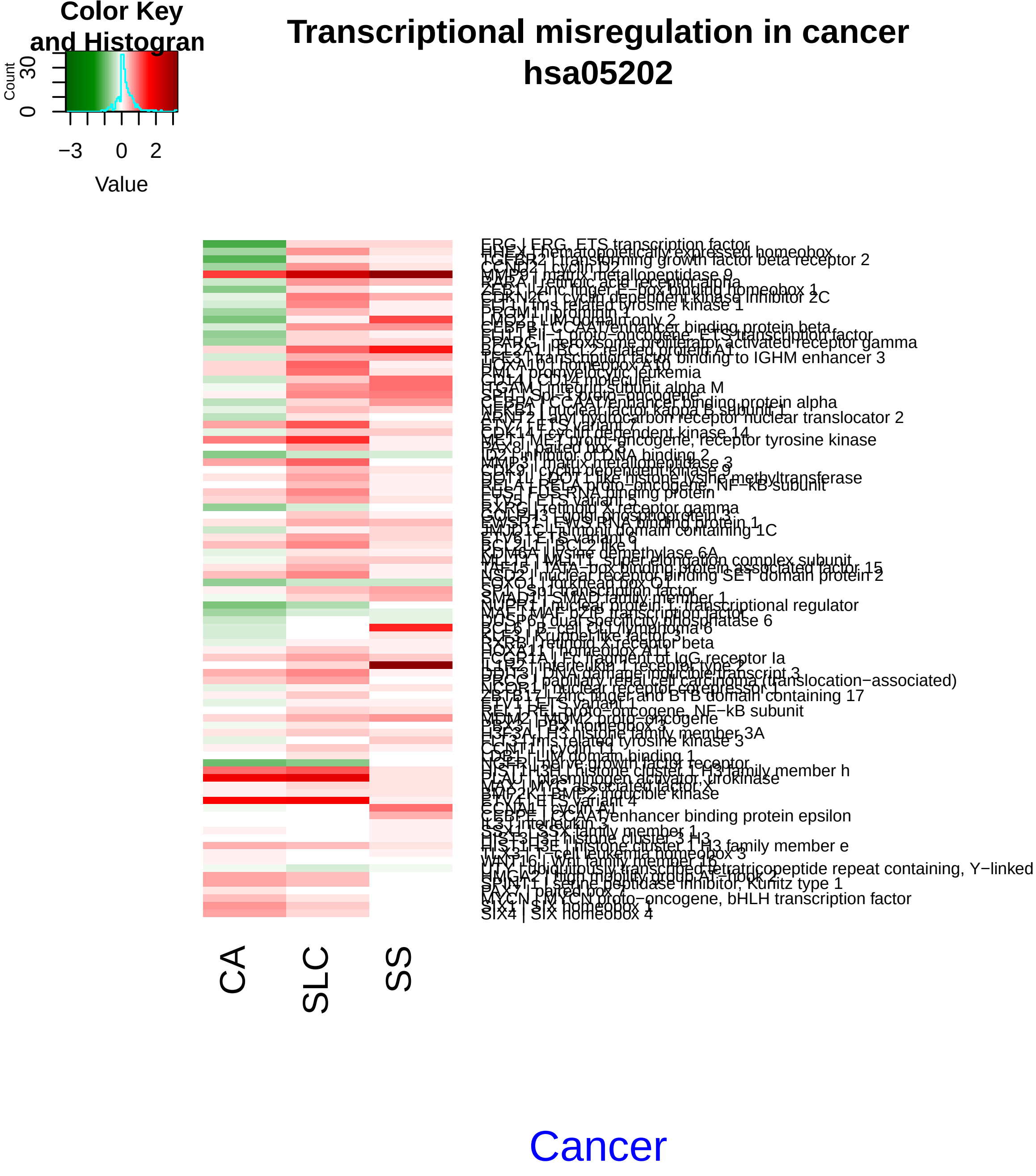

Color Key and Histogram
Count
0
30
−3
0
2
Value
Transcriptional misregulation in cancer
hsa05202
ERG | ERG, ETS transcription factor
HHEX | hematopoietically expressed homeobox
TGFBR2 | transforming growth factor beta receptor 2
CCND2 | cyclin D2
MMP9 | matrix metallopeptidase 9
RARA | retinoic acid receptor alpha
ZEB1 | zinc finger E−box binding homeobox 1
CDKN2C | cyclin dependent kinase inhibitor 2C
FLT1 | fms related tyrosine kinase 1
PROM1 | prominin 1
LMO2 | LIM domain only 2
CEBPB | CCAAT/enhancer binding protein beta
FLI1 | Fli−1 proto−oncogene, ETS transcription factor
PPARG | peroxisome proliferator activated receptor gamma
BCL2A1 | BCL2 related protein A1
TFE3 | transcription factor binding to IGHM enhancer 3
HOXA10 | homeobox A10
PML | promyelocytic leukemia
CD14 | CD14 molecule
ITGAM | integrin subunit alpha M
SPI1 | Spi−1 proto−oncogene
CEBPA | CCAAT/enhancer binding protein alpha
NFKB1 | nuclear factor kappa B subunit 1
ARNT2 | aryl hydrocarbon receptor nuclear translocator 2
CDK14 | cyclin dependent kinase 14
MET | MET proto−oncogene, receptor tyrosine kinase
PAX8 | paired box 8
ID2 | inhibitor of DNA binding 2
MMP3 | matrix metallopeptidase 3
CDK9 | cyclin dependent kinase 9
DOT1L | DOT1 like histone lysine methyltransferase
RELA | RELA proto−oncogene, NF−kB subunit
FUS | FUS RNA binding protein
ETV5 | ETS variant 5
RXRG | retinoid X receptor gamma
EWSR1 | EWS RNA binding protein 1
JMJD1C | jumonji domain containing 1C
ETV6 | ETS variant 6
BCL2L1 | BCL2 like 1
KDM6A | lysine demethylase 6A
TAF15 | TATA−box binding protein associated factor 15
FOXO1 | forkhead box O1
SP1 | Sp1 transcription factor
SMAD1 | SMAD family member 1
NUPR1 | nuclear protein 1, transcriptional regulator
MAF | MAF bZIP transcription factor
DUSP6 | dual specificity phosphatase 6
BCL6 | B−cell CLL/lymphoma 6
KLF3 | Kruppel like factor 3
RXRB | retinoid X receptor beta
HOXA11 | homeobox A11
FCGR1A | Fc fragment of IgG receptor Ia
IL1R2 | interleukin 1 receptor type 2
DDIT3 | DNA damage inducible transcript 3
PRCC | papillary renal cell carcinoma (translocation−associated)
NCOR1 | nuclear receptor corepressor 1
ZBTB17 | zinc finger and BTB domain containing 17
REL | REL proto−oncogene, NF−kB subunit
MDM2 | MDM2 proto−oncogene
PBX3 | PBX homeobox 3
H3F3A | H3 histone family member 3A
FLT3 | fms related tyrosine kinase 3
CCNT1 | cyclin T1
LDB1 | LIM domain binding 1
NGFR | nerve growth factor receptor
HIST1H3H | histone cluster 1 H3 family member h
PLAU | plasminogen activator, urokinase
MAX | MYC associated factor X
BMP2K | BMP2 inducible kinase
ETV4 | ETS variant 4
CCNA1 | cyclin A1
CEBPE | CCAAT/enhancer binding protein epsilon
IL3 | interleukin 3
SSX1 | SSX family member 1
HIST3H3 | histone cluster 3 H3
HIST1H3E | histone cluster 1 H3 family member e
TLX3 | T−cell leukemia homeobox 3
WNT16 | Wnt family member 16
UTY | ubiquitously transcribed tetratricopeptide repeat containing, Y−linked
HMGA2 | high mobility group AT−hook 2
SPINT1 | serine peptidase inhibitor, Kunitz type 1
PAX7 | paired box 7
MYCN | MYCN proto−oncogene, bHLH transcription factor
SIX1 | SIX homeobox 1
SIX4 | SIX homeobox 4
CA
SLC
SS
Cancer

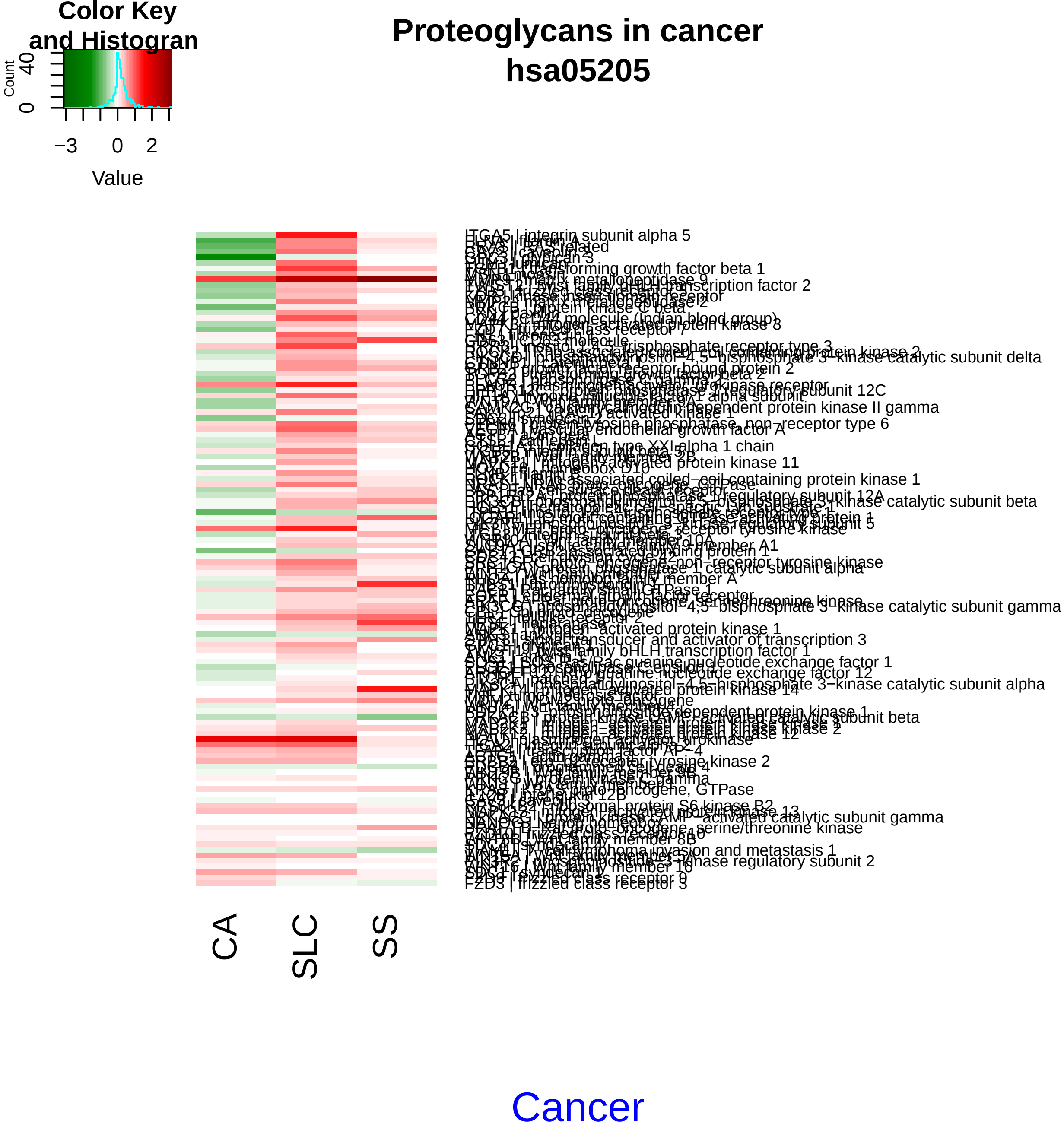

Color Key and Histogram
Count
0
40
−3
0
2
Value
Proteoglycans in cancer
hsa05205
ITGA5 | integrin subunit alpha 5
TGFB1 | transforming growth factor beta 1
MMP9 | matrix metallopeptidase 9
CD44 | CD44 molecule (Indian blood group)
COL21A1 | collagen type XXI alpha 1 chain
MAPK11 | mitogen-activated protein kinase 11
PIK3CG | phosphatidylinositol-4,5-bisphosphate 3-kinase catalytic subunit gamma
MAPK1 | mitogen-activated protein kinase 1
STAT3 | signal transducer and activator of transcription 3
TWIST1 | twist family bHLH transcription factor 1
PIK3CA | phosphatidylinositol-4,5-bisphosphate 3-kinase catalytic subunit alpha
ERBB2 | erb-b2 receptor tyrosine kinase 2
PRKACG | protein kinase cAMP-activated catalytic subunit gamma
FZD3 | frizzled class receptor 3
CA
SLC
SS
Cancer

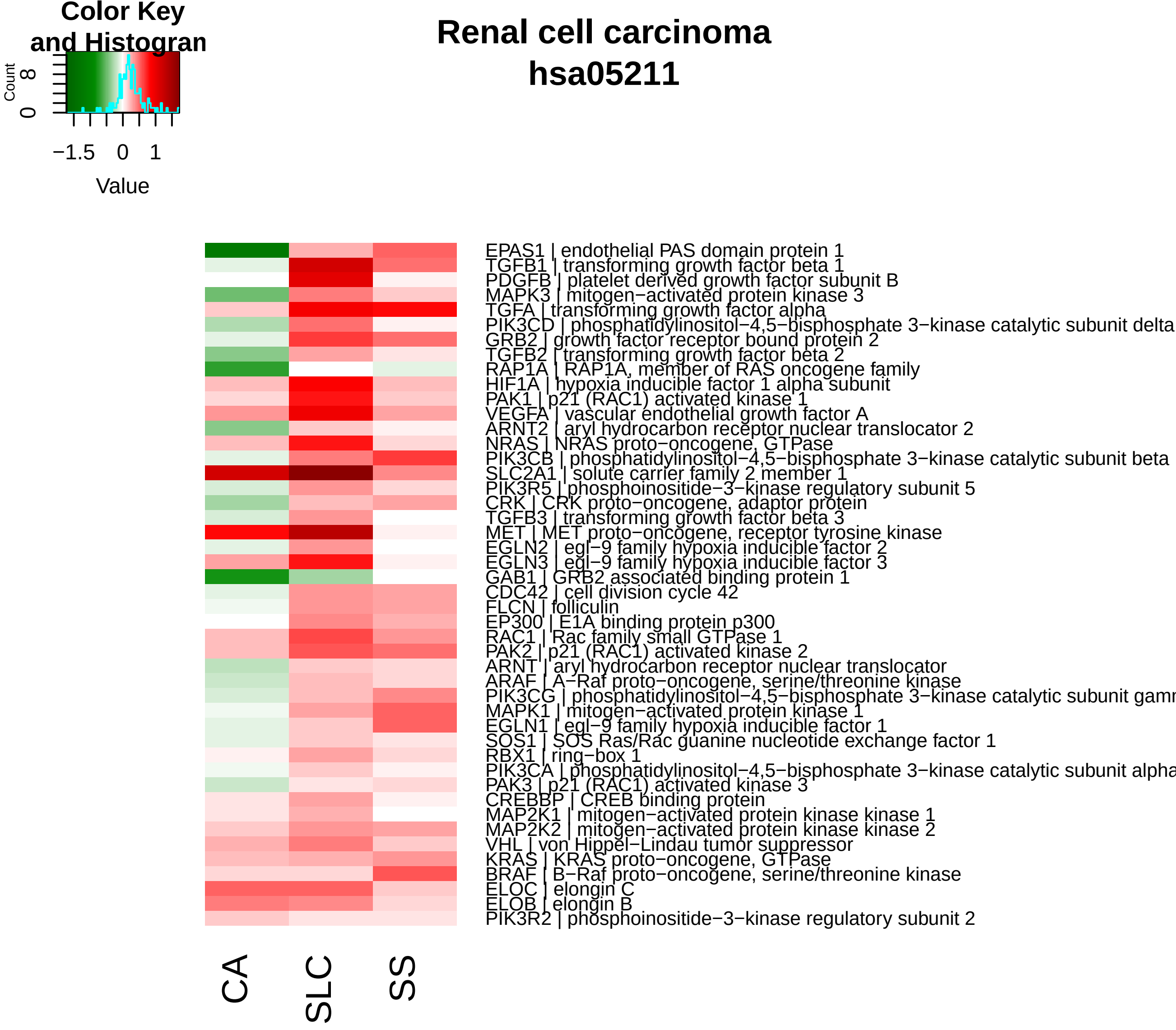
Renal cell carcinoma
hsa05211
Color Key
and Histogran
Count
0
8
−1.5
0
1
Value
EPAS1 | endothelial PAS domain protein 1
TGFB1 | transforming growth factor beta 1
PDGFB | platelet derived growth factor subunit B
MAPK3 | mitogen−activated protein kinase 3
TGFA | transforming growth factor alpha
PIK3CD | phosphatidylinositol−4,5−bisphosphate 3−kinase catalytic subunit delta
GRB2 | growth factor receptor bound protein 2
TGFB2 | transforming growth factor beta 2
RAP1A | RAP1A, member of RAS oncogene family
HIF1A | hypoxia inducible factor 1 alpha subunit
PAK1 | p21 (RAC1) activated kinase 1
VEGFA | vascular endothelial growth factor A
ARNT2 | aryl hydrocarbon receptor nuclear translocator 2
NRAS | NRAS proto−oncogene, GTPase
PIK3CB | phosphatidylinositol−4,5−bisphosphate 3−kinase catalytic subunit beta
SLC2A1 | solute carrier family 2 member 1
PIK3R5 | phosphoinositide−3−kinase regulatory subunit 5
CRK | CRK proto−oncogene, adaptor protein
TGFB3 | transforming growth factor beta 3
MET | MET proto−oncogene, receptor tyrosine kinase
EGLN2 | egl−9 family hypoxia inducible factor 2
EGLN3 | egl−9 family hypoxia inducible factor 3
GAB1 | GRB2 associated binding protein 1
CDC42 | cell division cycle 42
FLCN | folliculin
EP300 | E1A binding protein p300
RAC1 | Rac family small GTPase 1
PAK2 | p21 (RAC1) activated kinase 2
ARNT | aryl hydrocarbon receptor nuclear translocator
ARAF | A−Raf proto−oncogene, serine/threonine kinase
PIK3CG | phosphatidylinositol−4,5−bisphosphate 3−kinase catalytic subunit gamr
MAPK1 | mitogen−activated protein kinase 1
EGLN1 | egl−9 family hypoxia inducible factor 1
SOS1 | SOS Ras/Rac guanine nucleotide exchange factor 1
RBX1 | ring−box 1
PIK3CA | phosphatidylinositol−4,5−bisphosphate 3−kinase catalytic subunit alpha
PAK3 | p21 (RAC1) activated kinase 3
CREBBP | CREB binding protein
MAP2K1 | mitogen−activated protein kinase kinase 1
MAP2K2 | mitogen−activated protein kinase kinase 2
VHL | von Hippel−Lindau tumor suppressor
KRAS | KRAS proto−oncogene, GTPase
BRAF | B−Raf proto−oncogene, serine/threonine kinase
ELOC | elongin C
ELOB | elongin B
PIK3R2 | phosphoinositide−3−kinase regulatory subunit 2
CA
SLC
SS

Cancer

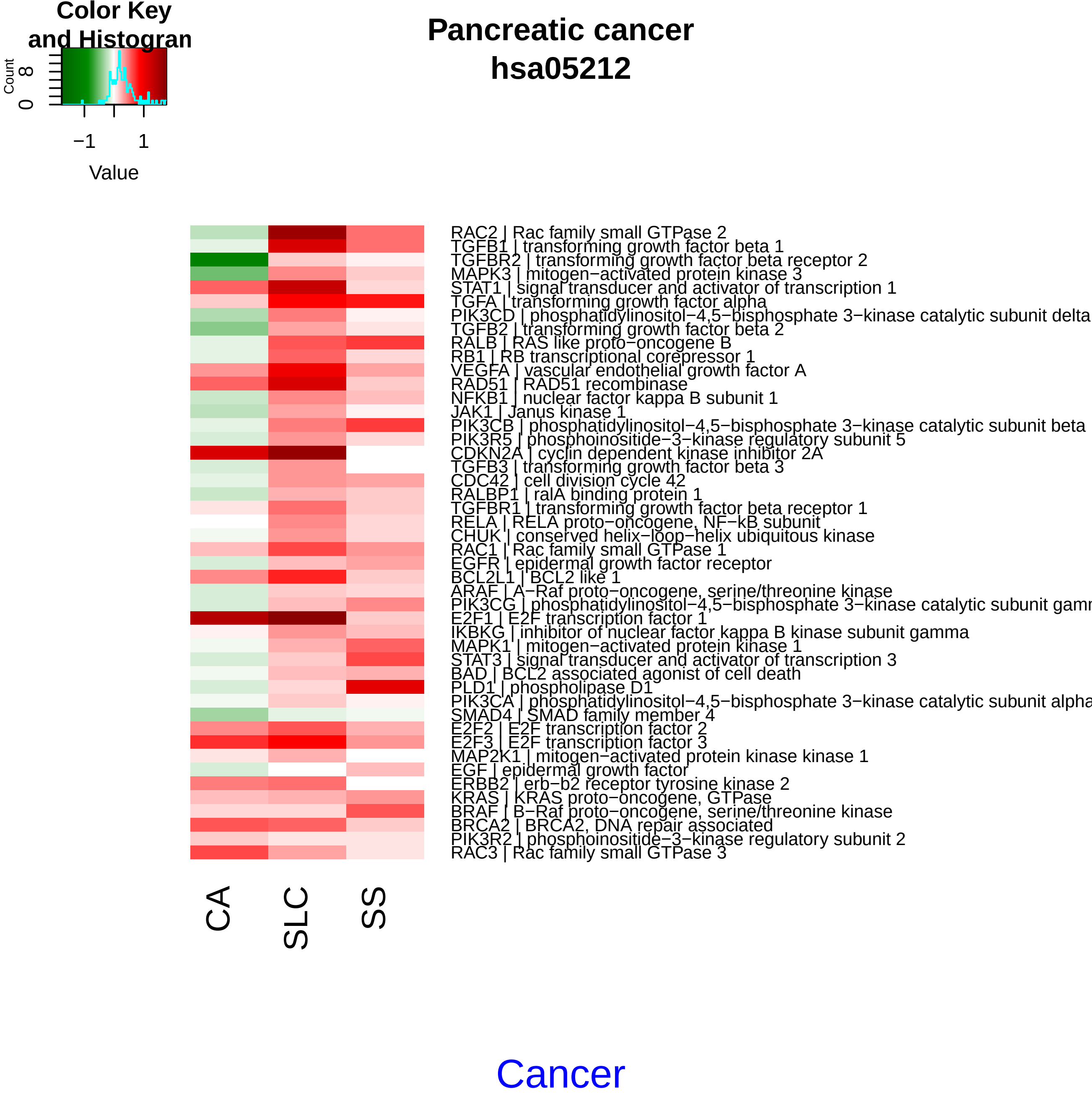

Color Key
and Histogran
Count
0
8
−1
1
Value
Pancreatic cancer
hsa05212
RAC2 | Rac family small GTPase 2
TGFB1 | transforming growth factor beta 1
TGFBR2 | transforming growth factor beta receptor 2
MAPK3 | mitogen−activated protein kinase 3
STAT1 | signal transducer and activator of transcription 1
TGFA | transforming growth factor alpha
PIK3CD | phosphatidylinositol−4,5−bisphosphate 3−kinase catalytic subunit delta
TGFB2 | transforming growth factor beta 2
RALB | RAS like proto−oncogene B
RB1 | RB transcriptional corepressor 1
VEGFA | vascular endothelial growth factor A
RAD51 | RAD51 recombinase
NFKB1 | nuclear factor kappa B subunit 1
JAK1 | Janus kinase 1
PIK3CB | phosphatidylinositol−4,5−bisphosphate 3−kinase catalytic subunit beta
PIK3R5 | phosphoinositide−3−kinase regulatory subunit 5
CDKN2A | cyclin dependent kinase inhibitor 2A
TGFB3 | transforming growth factor beta 3
CDC42 | cell division cycle 42
RALBP1 | ralA binding protein 1
TGFBR1 | transforming growth factor beta receptor 1
RELA | RELA proto−oncogene, NF−kB subunit
CHUK | conserved helix−loop−helix ubiquitous kinase
RAC1 | Rac family small GTPase 1
EGFR | epidermal growth factor receptor
BCL2L1 | BCL2 like 1
ARAF | A−Raf proto−oncogene, serine/threonine kinase
PIK3CG | phosphatidylinositol−4,5−bisphosphate 3−kinase catalytic subunit gamr
E2F1 | E2F transcription factor 1
IKBKG | inhibitor of nuclear factor kappa B kinase subunit gamma
MAPK1 | mitogen−activated protein kinase 1
STAT3 | signal transducer and activator of transcription 3
BAD | BCL2 associated agonist of cell death
PLD1 | phospholipase D1
PIK3CA | phosphatidylinositol−4,5−bisphosphate 3−kinase catalytic subunit alpha
SMAD4 | SMAD family member 4
E2F2 | E2F transcription factor 2
E2F3 | E2F transcription factor 3
MAP2K1 | mitogen−activated protein kinase kinase 1
EGF | epidermal growth factor
ERBB2 | erb−b2 receptor tyrosine kinase 2
KRAS | KRAS proto−oncogene, GTPase
BRAF | B−Raf proto−oncogene, serine/threonine kinase
BRCA2 | BRCA2, DNA repair associated
PIK3R2 | phosphoinositide−3−kinase regulatory subunit 2
RAC3 | Rac family small GTPase 3
CA
SLC
SS
Cancer

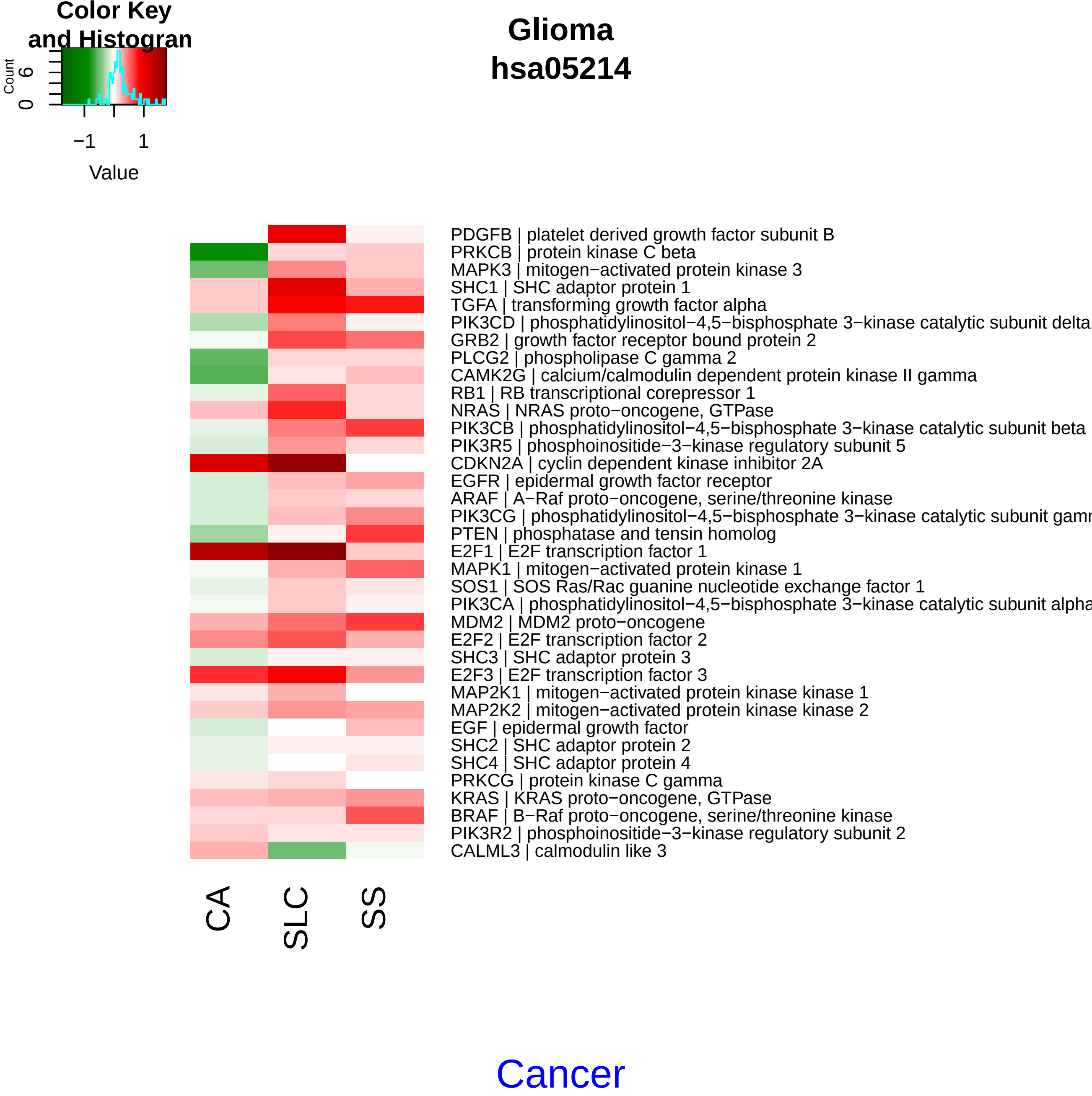
Color Key
and Histogram
Count
0
6
−1
1
Value
Glioma
hsa05214
PDGFB | platelet derived growth factor subunit B
PRKCB | protein kinase C beta
MAPK3 | mitogen−activated protein kinase 3
SHC1 | SHC adaptor protein 1
TGFA | transforming growth factor alpha
PIK3CD | phosphatidylinositol−4,5−bisphosphate 3−kinase catalytic subunit delta
GRB2 | growth factor receptor bound protein 2
PLCG2 | phospholipase C gamma 2
CAMK2G | calcium/calmodulin dependent protein kinase II gamma
RB1 | RB transcriptional corepressor 1
NRAS | NRAS proto−oncogene, GTPase
PIK3CB | phosphatidylinositol−4,5−bisphosphate 3−kinase catalytic subunit beta
PIK3R5 | phosphoinositide−3−kinase regulatory subunit 5
CDKN2A | cyclin dependent kinase inhibitor 2A
EGFR | epidermal growth factor receptor
ARAF | A−Raf proto−oncogene, serine/threonine kinase
PIK3CG | phosphatidylinositol−4,5−bisphosphate 3−kinase catalytic subunit gamr
PTEN | phosphatase and tensin homolog
E2F1 | E2F transcription factor 1
MAPK1 | mitogen−activated protein kinase 1
SOS1 | SOS Ras/Rac guanine nucleotide exchange factor 1
PIK3CA | phosphatidylinositol−4,5−bisphosphate 3−kinase catalytic subunit alpha
MDM2 | MDM2 proto−oncogene
E2F2 | E2F transcription factor 2
SHC3 | SHC adaptor protein 3
E2F3 | E2F transcription factor 3
MAP2K1 | mitogen−activated protein kinase kinase 1
MAP2K2 | mitogen−activated protein kinase kinase 2
EGF | epidermal growth factor
SHC2 | SHC adaptor protein 2
SHC4 | SHC adaptor protein 4
PRKCG | protein kinase C gamma
KRAS | KRAS proto−oncogene, GTPase
BRAF | B−Raf proto−oncogene, serine/threonine kinase
PIK3R2 | phosphoinositide−3−kinase regulatory subunit 2
CALML3 | calmodulin like 3
CA
SLC
SS
Cancer

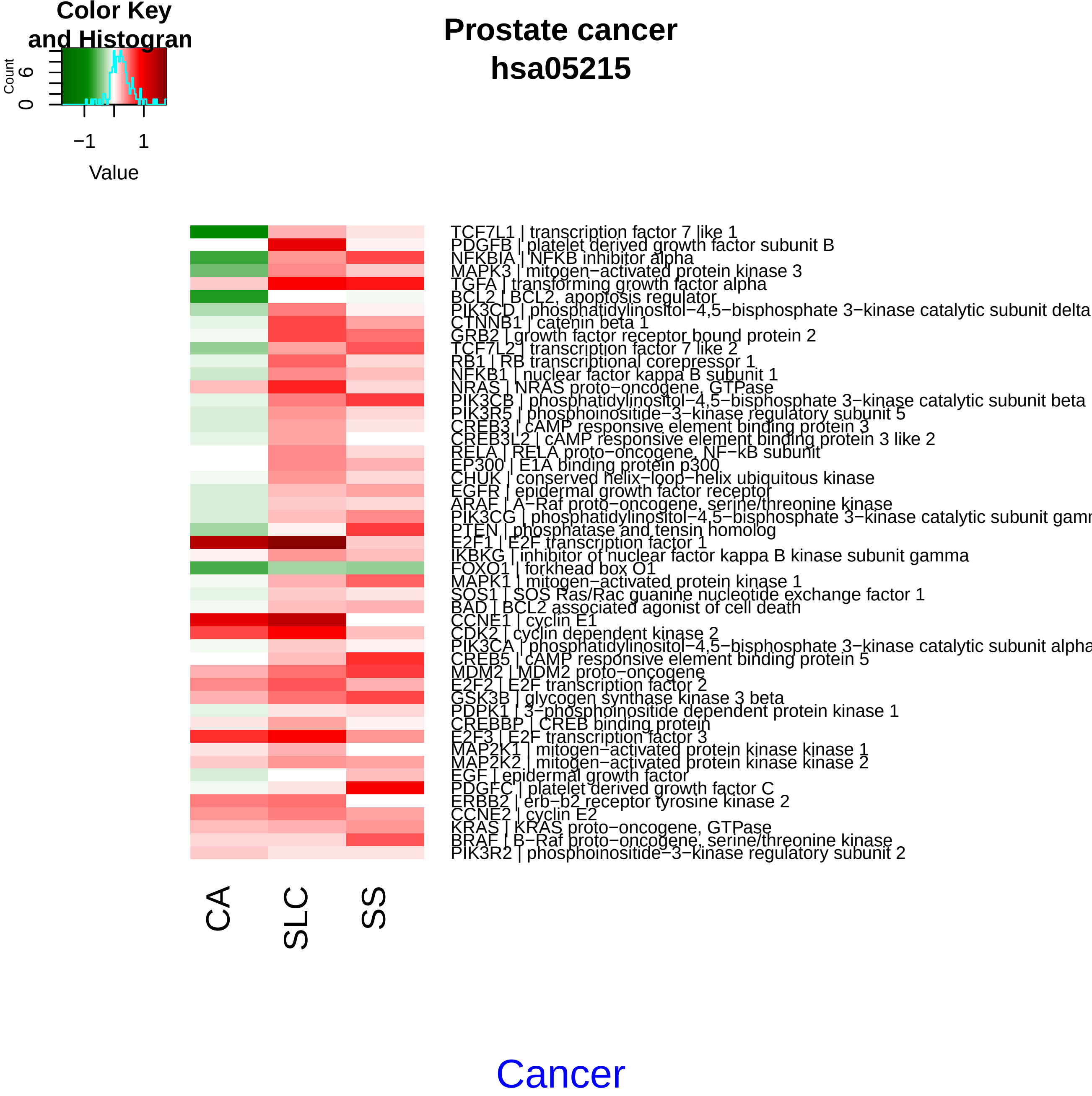

Color Key
and Histogran
Count
0
6
−1
1
Value
Prostate cancer
hsa05215
TCF7L1 | transcription factor 7 like 1
PDGFB | platelet derived growth factor subunit B
NFKBIA | NFKB inhibitor alpha
MAPK3 | mitogen−activated protein kinase 3
TGFA | transforming growth factor alpha
BCL2 | BCL2, apoptosis regulator
PIK3CD | phosphatidylinositol−4,5−bisphosphate 3−kinase catalytic subunit delta
CTNNB1 | catenin beta 1
GRB2 | growth factor receptor bound protein 2
TCF7L2 | transcription factor 7 like 2
RB1 | RB transcriptional corepressor 1
NFKB1 | nuclear factor kappa B subunit 1
NRAS | NRAS proto−oncogene, GTPase
PIK3CB | phosphatidylinositol−4,5−bisphosphate 3−kinase catalytic subunit beta
PIK3R5 | phosphoinositide−3−kinase regulatory subunit 5
CREB3 | cAMP responsive element binding protein 3
CREB3L2 | cAMP responsive element binding protein 3 like 2
RELA | RELA proto−oncogene, NF−kB subunit
EP300 | E1A binding protein p300
CHUK | conserved helix−loop−helix ubiquitous kinase
EGFR | epidermal growth factor receptor
ARAF | A−Raf proto−oncogene, serine/threonine kinase
PIK3CG | phosphatidylinositol−4,5−bisphosphate 3−kinase catalytic subunit gamr
PTEN | phosphatase and tensin homolog
E2F1 | E2F transcription factor 1
IKBKG | inhibitor of nuclear factor kappa B kinase subunit gamma
FOXO1 | forkhead box O1
MAPK1 | mitogen−activated protein kinase 1
SOS1 | SOS Ras/Rac guanine nucleotide exchange factor 1
BAD | BCL2 associated agonist of cell death
CCNE1 | cyclin E1
CDK2 | cyclin dependent kinase 2
PIK3CA | phosphatidylinositol−4,5−bisphosphate 3−kinase catalytic subunit alpha
CREB5 | cAMP responsive element binding protein 5
MDM2 | MDM2 proto−oncogene
E2F2 | E2F transcription factor 2
GSK3B | glycogen synthase kinase 3 beta
PDPK1 | 3−phosphoinositide dependent protein kinase 1
CREBBP | CREB binding protein
E2F3 | E2F transcription factor 3
MAP2K1 | mitogen−activated protein kinase kinase 1
MAP2K2 | mitogen−activated protein kinase kinase 2
EGF | epidermal growth factor
PDGFC | platelet derived growth factor C
ERBB2 | erb−b2 receptor tyrosine kinase 2
CCNE2 | cyclin E2
KRAS | KRAS proto−oncogene, GTPase
BRAF | B−Raf proto−oncogene, serine/threonine kinase
PIK3R2 | phosphoinositide−3−kinase regulatory subunit 2
CA
SLC
SS
Cancer

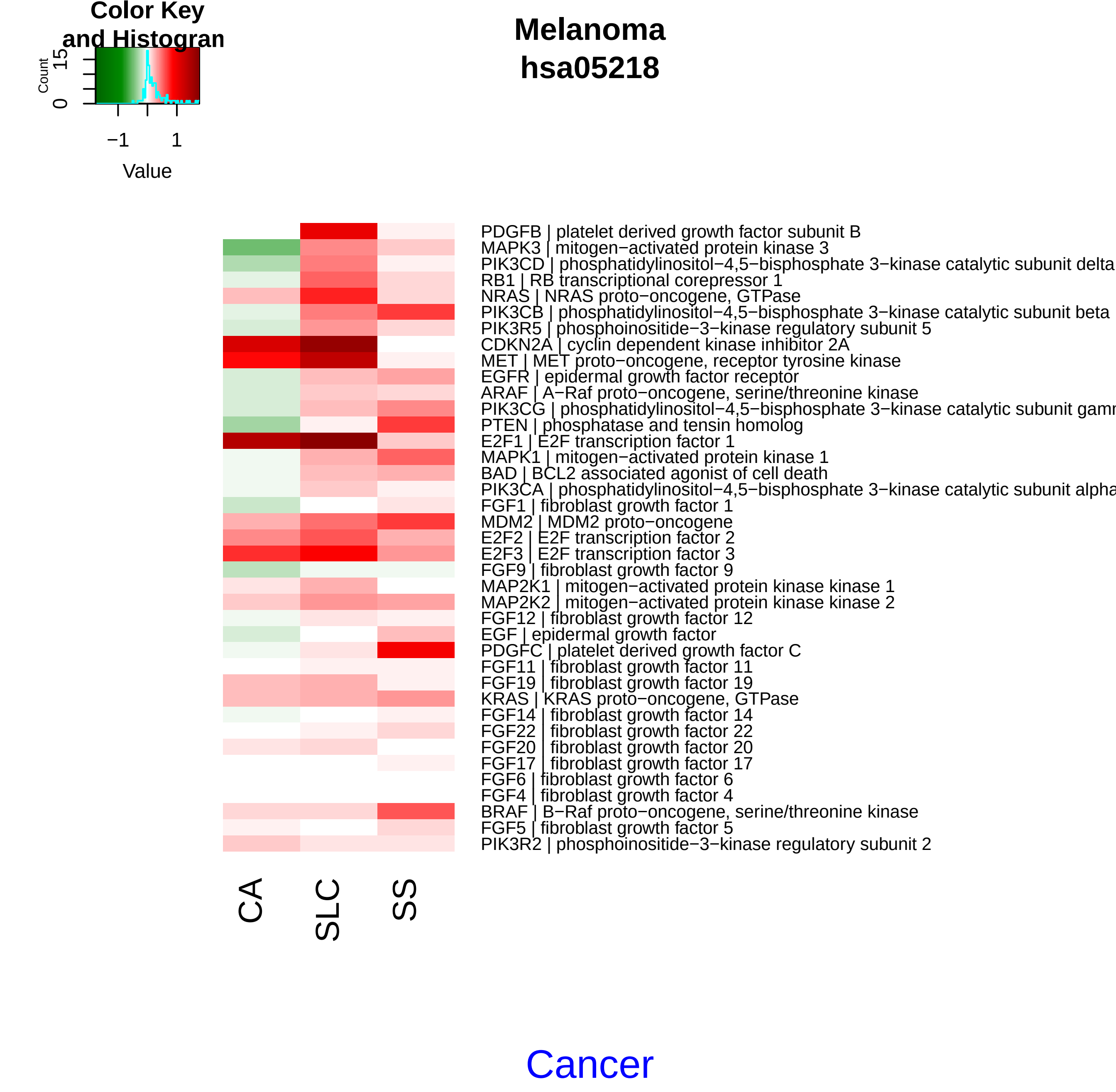
Color Key
and Histogran
Count
0
15
−1
1
Value
Melanoma
hsa05218
PDGFB | platelet derived growth factor subunit B
MAPK3 | mitogen−activated protein kinase 3
PIK3CD | phosphatidylinositol−4,5−bisphosphate 3−kinase catalytic subunit delta
RB1 | RB transcriptional corepressor 1
NRAS | NRAS proto−oncogene, GTPase
PIK3CB | phosphatidylinositol−4,5−bisphosphate 3−kinase catalytic subunit beta
PIK3R5 | phosphoinositide−3−kinase regulatory subunit 5
CDKN2A | cyclin dependent kinase inhibitor 2A
MET | MET proto−oncogene, receptor tyrosine kinase
EGFR | epidermal growth factor receptor
ARAF | A−Raf proto−oncogene, serine/threonine kinase
PIK3CG | phosphatidylinositol−4,5−bisphosphate 3−kinase catalytic subunit gamr
PTEN | phosphatase and tensin homolog
E2F1 | E2F transcription factor 1
MAPK1 | mitogen−activated protein kinase 1
BAD | BCL2 associated agonist of cell death
PIK3CA | phosphatidylinositol−4,5−bisphosphate 3−kinase catalytic subunit alpha
FGF1 | fibroblast growth factor 1
MDM2 | MDM2 proto−oncogene
E2F2 | E2F transcription factor 2
E2F3 | E2F transcription factor 3
FGF9 | fibroblast growth factor 9
MAP2K1 | mitogen−activated protein kinase kinase 1
MAP2K2 | mitogen−activated protein kinase kinase 2
FGF12 | fibroblast growth factor 12
EGF | epidermal growth factor
PDGFC | platelet derived growth factor C
FGF11 | fibroblast growth factor 11
FGF19 | fibroblast growth factor 19
KRAS | KRAS proto−oncogene, GTPase
FGF14 | fibroblast growth factor 14
FGF22 | fibroblast growth factor 22
FGF20 | fibroblast growth factor 20
FGF17 | fibroblast growth factor 17
FGF6 | fibroblast growth factor 6
FGF4 | fibroblast growth factor 4
BRAF | B−Raf proto−oncogene, serine/threonine kinase
FGF5 | fibroblast growth factor 5
PIK3R2 | phosphoinositide−3−kinase regulatory subunit 2
CA
SLC
SS
Cancer

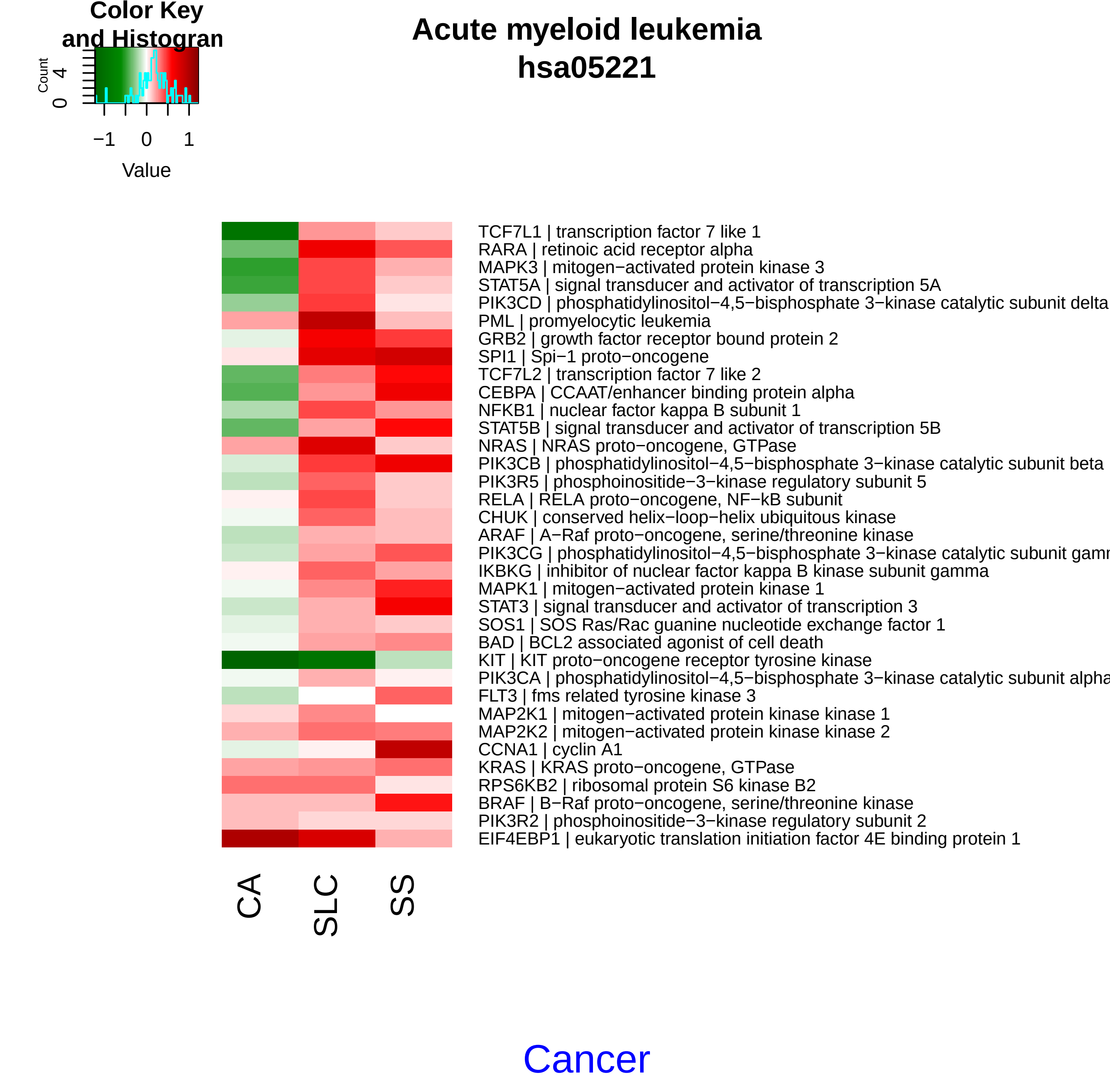

Color Key
and Histogran
Count
0
4
−1
0
1
Value
Acute myeloid leukemia
hsa05221
TCF7L1 | transcription factor 7 like 1
RARA | retinoic acid receptor alpha
MAPK3 | mitogen−activated protein kinase 3
STAT5A | signal transducer and activator of transcription 5A
PIK3CD | phosphatidylinositol−4,5−bisphosphate 3−kinase catalytic subunit delta
PML | promyelocytic leukemia
GRB2 | growth factor receptor bound protein 2
SPI1 | Spi−1 proto−oncogene
TCF7L2 | transcription factor 7 like 2
CEBPA | CCAAT/enhancer binding protein alpha
NFKB1 | nuclear factor kappa B subunit 1
STAT5B | signal transducer and activator of transcription 5B
NRAS | NRAS proto−oncogene, GTPase
PIK3CB | phosphatidylinositol−4,5−bisphosphate 3−kinase catalytic subunit beta
PIK3R5 | phosphoinositide−3−kinase regulatory subunit 5
RELA | RELA proto−oncogene, NF−kB subunit
CHUK | conserved helix−loop−helix ubiquitous kinase
ARAF | A−Raf proto−oncogene, serine/threonine kinase
PIK3CG | phosphatidylinositol−4,5−bisphosphate 3−kinase catalytic subunit gamr
IKBKG | inhibitor of nuclear factor kappa B kinase subunit gamma
MAPK1 | mitogen−activated protein kinase 1
STAT3 | signal transducer and activator of transcription 3
SOS1 | SOS Ras/Rac guanine nucleotide exchange factor 1
BAD | BCL2 associated agonist of cell death
KIT | KIT proto−oncogene receptor tyrosine kinase
PIK3CA | phosphatidylinositol−4,5−bisphosphate 3−kinase catalytic subunit alpha
FLT3 | fms related tyrosine kinase 3
MAP2K1 | mitogen−activated protein kinase kinase 1
MAP2K2 | mitogen−activated protein kinase kinase 2
CCNA1 | cyclin A1
KRAS | KRAS proto−oncogene, GTPase
RPS6KB2 | ribosomal protein S6 kinase B2
BRAF | B−Raf proto−oncogene, serine/threonine kinase
PIK3R2 | phosphoinositide−3−kinase regulatory subunit 2
EIF4EBP1 | eukaryotic translation initiation factor 4E binding protein 1
CA
SLC
SS
Cancer

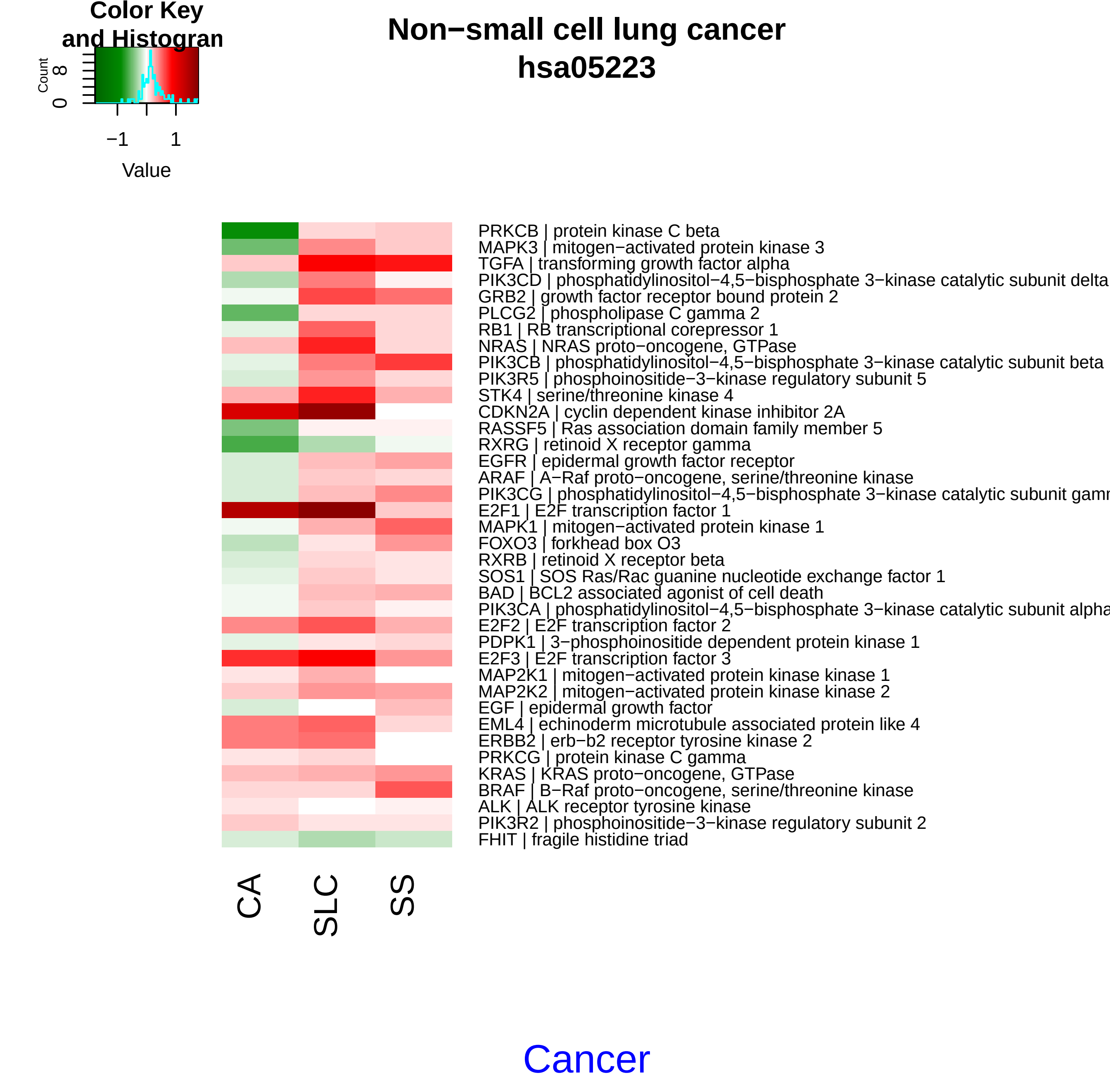
Color Key and Histogram
Count
0
8
−1
1
Value
Non−small cell lung cancer
hsa05223
PRKCB | protein kinase C beta
MAPK3 | mitogen−activated protein kinase 3
TGFA | transforming growth factor alpha
PIK3CD | phosphatidylinositol−4,5−bisphosphate 3−kinase catalytic subunit delta
GRB2 | growth factor receptor bound protein 2
PLCG2 | phospholipase C gamma 2
RB1 | RB transcriptional corepressor 1
NRAS | NRAS proto−oncogene, GTPase
PIK3CB | phosphatidylinositol−4,5−bisphosphate 3−kinase catalytic subunit beta
PIK3R5 | phosphoinositide−3−kinase regulatory subunit 5
STK4 | serine/threonine kinase 4
CDKN2A | cyclin dependent kinase inhibitor 2A
RASSF5 | Ras association domain family member 5
RXRG | retinoid X receptor gamma
EGFR | epidermal growth factor receptor
ARAF | A−Raf proto−oncogene, serine/threonine kinase
PIK3CG | phosphatidylinositol−4,5−bisphosphate 3−kinase catalytic subunit gamm
E2F1 | E2F transcription factor 1
MAPK1 | mitogen−activated protein kinase 1
FOXO3 | forkhead box O3
RXRB | retinoid X receptor beta
SOS1 | SOS Ras/Rac guanine nucleotide exchange factor 1
BAD | BCL2 associated agonist of cell death
PIK3CA | phosphatidylinositol−4,5−bisphosphate 3−kinase catalytic subunit alpha
E2F2 | E2F transcription factor 2
PDPK1 | 3−phosphoinositide dependent protein kinase 1
E2F3 | E2F transcription factor 3
MAP2K1 | mitogen−activated protein kinase kinase 1
MAP2K2 | mitogen−activated protein kinase kinase 2
EGF | epidermal growth factor
EML4 | echinoderm microtubule associated protein like 4
ERBB2 | erb−b2 receptor tyrosine kinase 2
PRKCG | protein kinase C gamma
KRAS | KRAS proto−oncogene, GTPase
BRAF | B−Raf proto−oncogene, serine/threonine kinase
ALK | ALK receptor tyrosine kinase
PIK3R2 | phosphoinositide−3−kinase regulatory subunit 2
FHIT | fragile histidine triad
CA
SLC
SS
Cancer

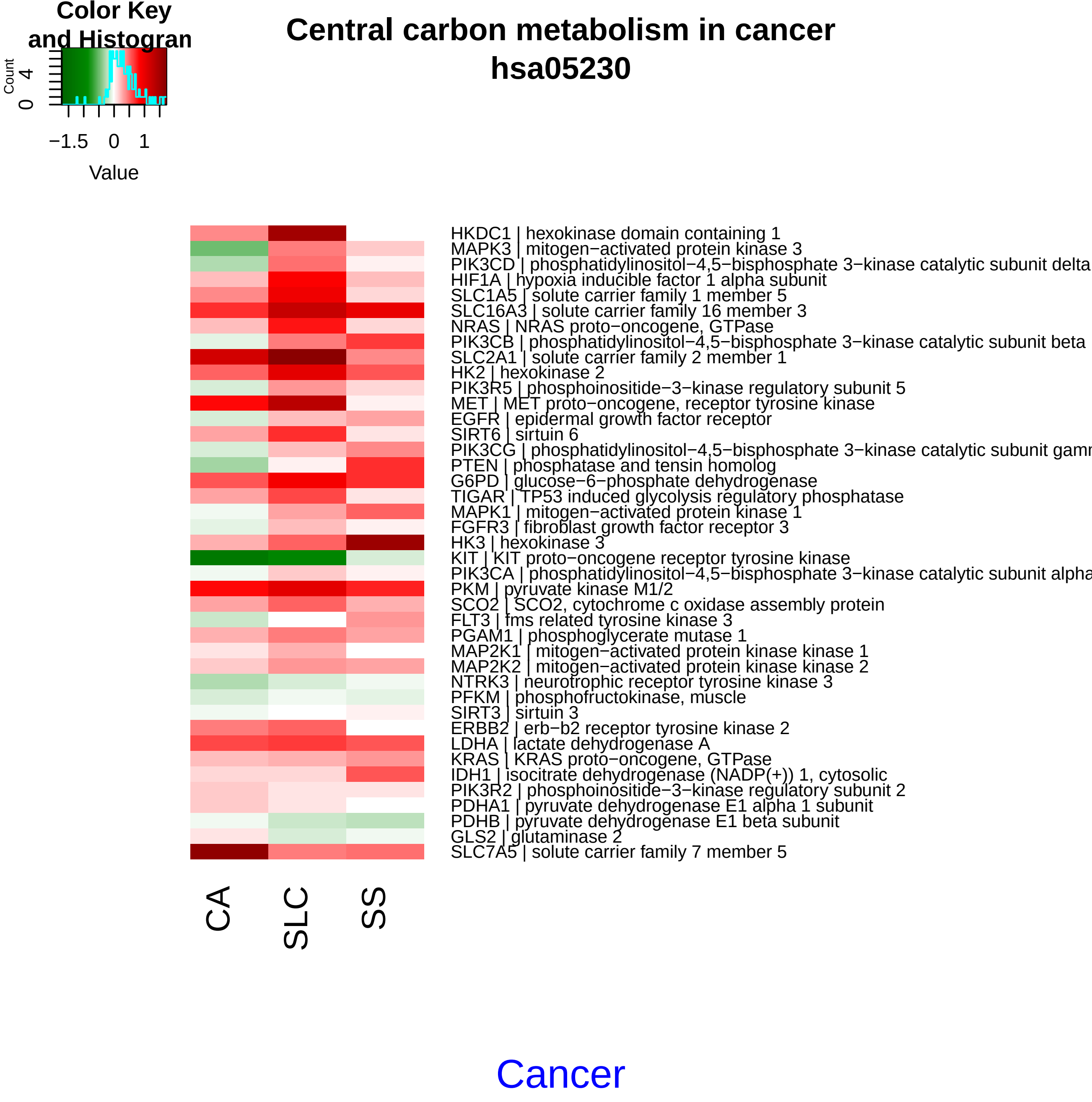

Central carbon metabolism in cancer
hsa05230
Color Key
and Histogran
Count
0
4
−1.5
0
1
Value
HKDC1 | hexokinase domain containing 1
MAPK3 | mitogen−activated protein kinase 3
PIK3CD | phosphatidylinositol−4,5−bisphosphate 3−kinase catalytic subunit delta
HIF1A | hypoxia inducible factor 1 alpha subunit
SLC1A5 | solute carrier family 1 member 5
SLC16A3 | solute carrier family 16 member 3
NRAS | NRAS proto−oncogene, GTPase
PIK3CB | phosphatidylinositol−4,5−bisphosphate 3−kinase catalytic subunit beta
SLC2A1 | solute carrier family 2 member 1
HK2 | hexokinase 2
PIK3R5 | phosphoinositide−3−kinase regulatory subunit 5
MET | MET proto−oncogene, receptor tyrosine kinase
EGFR | epidermal growth factor receptor
SIRT6 | sirtuin 6
PIK3CG | phosphatidylinositol−4,5−bisphosphate 3−kinase catalytic subunit gamr
PTEN | phosphatase and tensin homolog
G6PD | glucose−6−phosphate dehydrogenase
TIGAR | TP53 induced glycolysis regulatory phosphatase
MAPK1 | mitogen−activated protein kinase 1
FGFR3 | fibroblast growth factor receptor 3
HK3 | hexokinase 3
KIT | KIT proto−oncogene receptor tyrosine kinase
PIK3CA | phosphatidylinositol−4,5−bisphosphate 3−kinase catalytic subunit alpha
PKM | pyruvate kinase M1/2
SCO2 | SCO2, cytochrome c oxidase assembly protein
FLT3 | fms related tyrosine kinase 3
PGAM1 | phosphoglycerate mutase 1
MAP2K1 | mitogen−activated protein kinase kinase 1
MAP2K2 | mitogen−activated protein kinase kinase 2
NTRK3 | neurotrophic receptor tyrosine kinase 3
PFKM | phosphofructokinase, muscle
SIRT3 | sirtuin 3
ERBB2 | erb−b2 receptor tyrosine kinase 2
LDHA | lactate dehydrogenase A
KRAS | KRAS proto−oncogene, GTPase
IDH1 | isocitrate dehydrogenase (NADP(+)) 1, cytosolic
PIK3R2 | phosphoinositide−3−kinase regulatory subunit 2
PDHA1 | pyruvate dehydrogenase E1 alpha 1 subunit
PDHB | pyruvate dehydrogenase E1 beta subunit
GLS2 | glutaminase 2
SLC7A5 | solute carrier family 7 member 5
CA
SLC
SS
Cancer

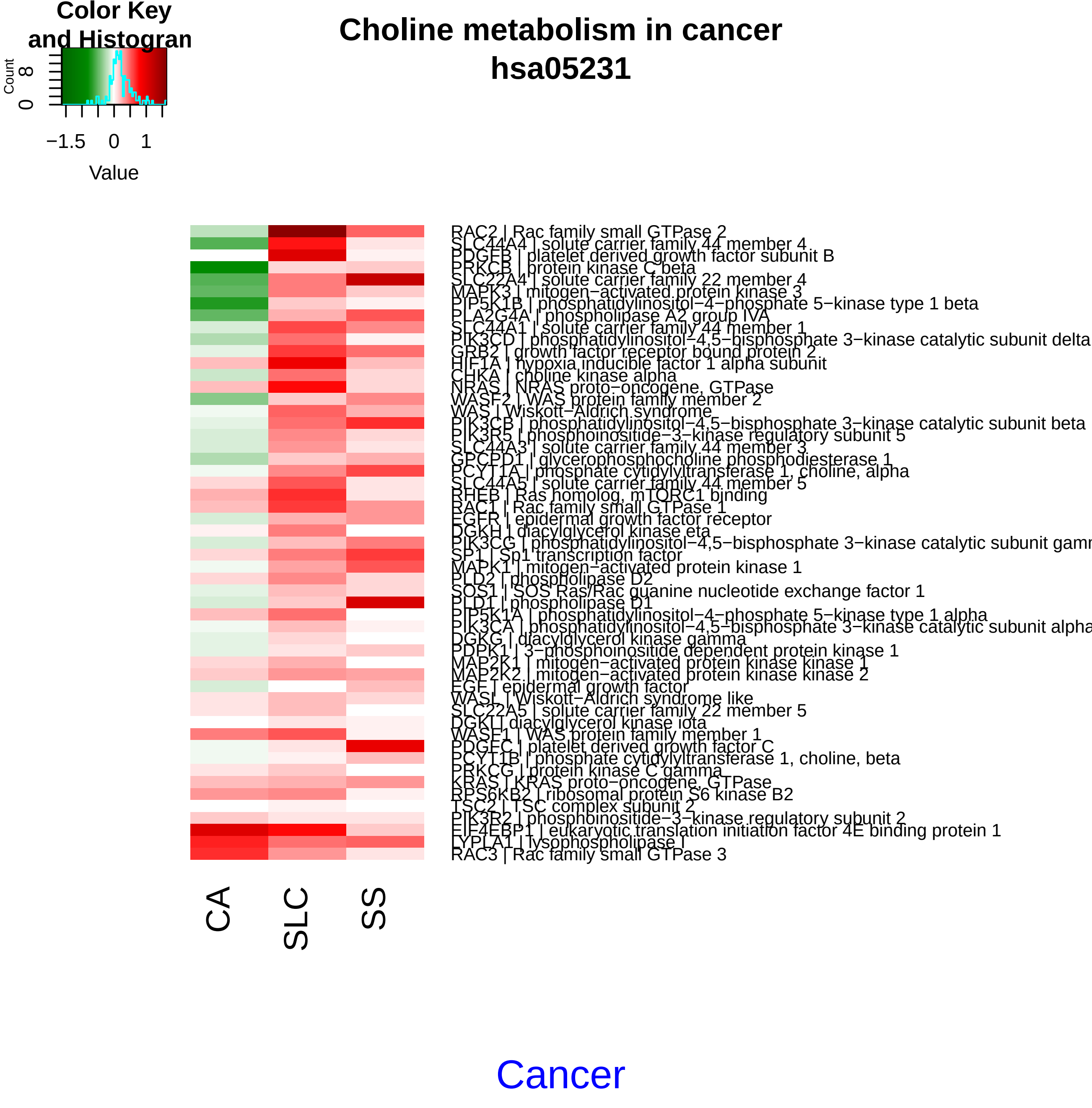
Color Key
and Histogran
Count
0
8
−1.5
0
1
Value
Choline metabolism in cancer
hsa05231
RAC2 | Rac family small GTPase 2
SLC44A4 | solute carrier family 44 member 4
PDGFB | platelet derived growth factor subunit B
PRKCB | protein kinase C beta
SLC22A4 | solute carrier family 22 member 4
MAPK3 | mitogen−activated protein kinase 3
PIP5K1B | phosphatidylinositol−4−phosphate 5−kinase type 1 beta
PLA2G4A | phospholipase A2 group IVA
SLC44A1 | solute carrier family 44 member 1
PIK3CD | phosphatidylinositol−4,5−bisphosphate 3−kinase catalytic subunit delta
GRB2 | growth factor receptor bound protein 2
HIF1A | hypoxia inducible factor 1 alpha subunit
CHKA | choline kinase alpha
NRAS | NRAS proto−oncogene, GTPase
WASF2 | WAS protein family member 2
WAS | Wiskott−Aldrich syndrome
PIK3CB | phosphatidylinositol−4,5−bisphosphate 3−kinase catalytic subunit beta
PIK3R5 | phosphoinositide−3−kinase regulatory subunit 5
SLC44A3 | solute carrier family 44 member 3
GPCPD1 | glycerophosphocholine phosphodiesterase 1
PCYT1A | phosphate cytidylyltransferase 1, choline, alpha
SLC44A5 | solute carrier family 44 member 5
RHEB | Ras homolog, mTORC1 binding
RAC1 | Rac family small GTPase 1
EGFR | epidermal growth factor receptor
DGKH | diacylglycerol kinase eta
PIK3CG | phosphatidylinositol−4,5−bisphosphate 3−kinase catalytic subunit gamr
SP1 | Sp1 transcription factor
MAPK1 | mitogen−activated protein kinase 1
PLD2 | phospholipase D2
SOS1 | SOS Ras/Rac guanine nucleotide exchange factor 1
PLD1 | phospholipase D1
PIP5K1A | phosphatidylinositol−4−phosphate 5−kinase type 1 alpha
PIK3CA | phosphatidylinositol−4,5−bisphosphate 3−kinase catalytic subunit alpha
DGKG | diacylglycerol kinase gamma
PDPK1 | 3−phosphoinositide dependent protein kinase 1
MAP2K1 | mitogen−activated protein kinase kinase 1
MAP2K2 | mitogen−activated protein kinase kinase 2
EGF | epidermal growth factor
WASL | Wiskott−Aldrich syndrome like
SLC22A5 | solute carrier family 22 member 5
DGKI | diacylglycerol kinase iota
WASF1 | WAS protein family member 1
PDGFC | platelet derived growth factor C
PCYT1B | phosphate cytidylyltransferase 1, choline, beta
PRKCG | protein kinase C gamma
KRAS | KRAS proto−oncogene, GTPase
RPS6KB2 | ribosomal protein S6 kinase B2
TSC2 | TSC complex subunit 2
PIK3R2 | phosphoinositide−3−kinase regulatory subunit 2
EIF4EBP1 | eukaryotic translation initiation factor 4E binding protein 1
LYPLA1 | lysophospholipase I
RAC3 | Rac family small GTPase 3
CA
SLC
SS
Cancer

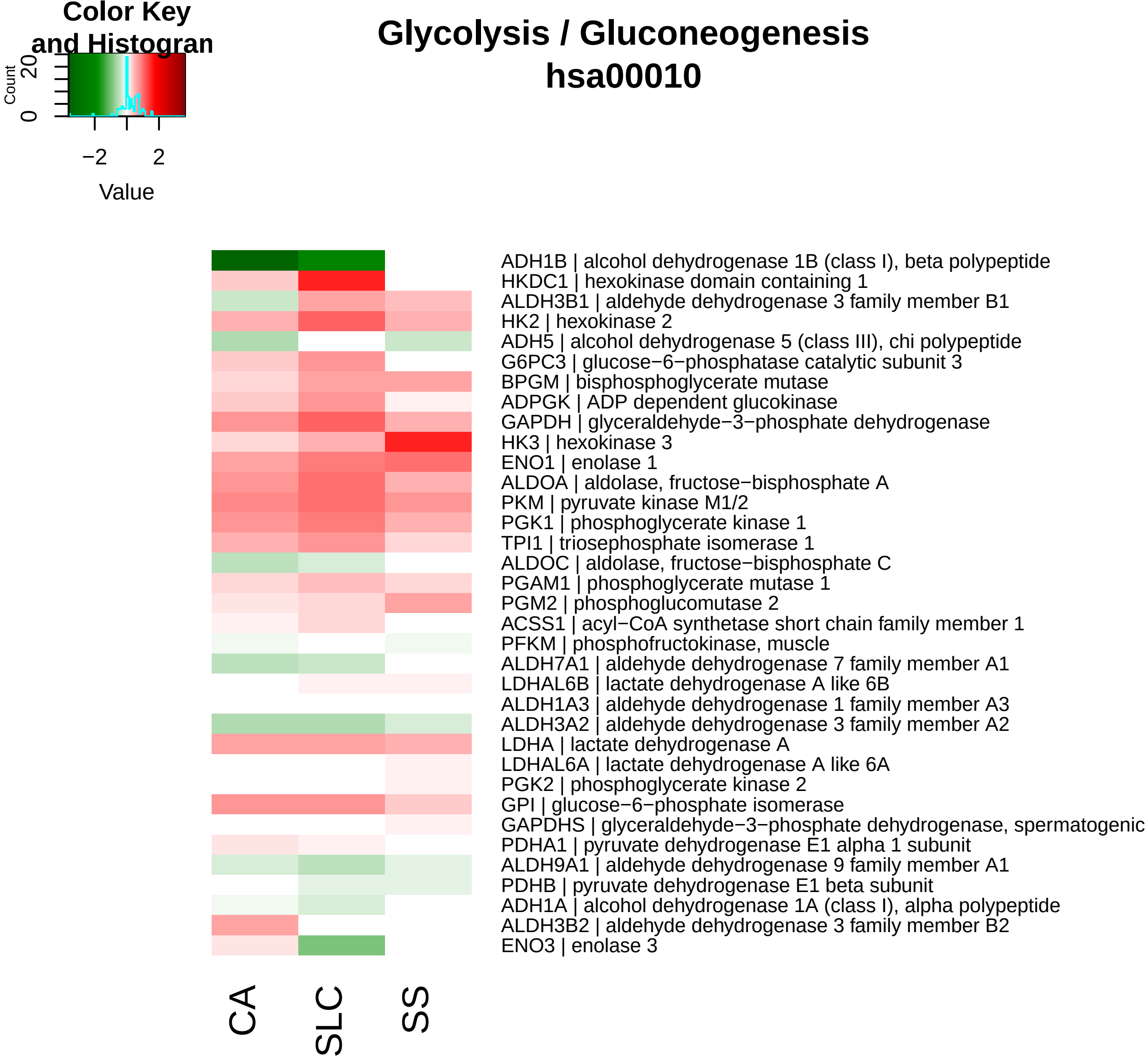

Carbohydrate metabolism

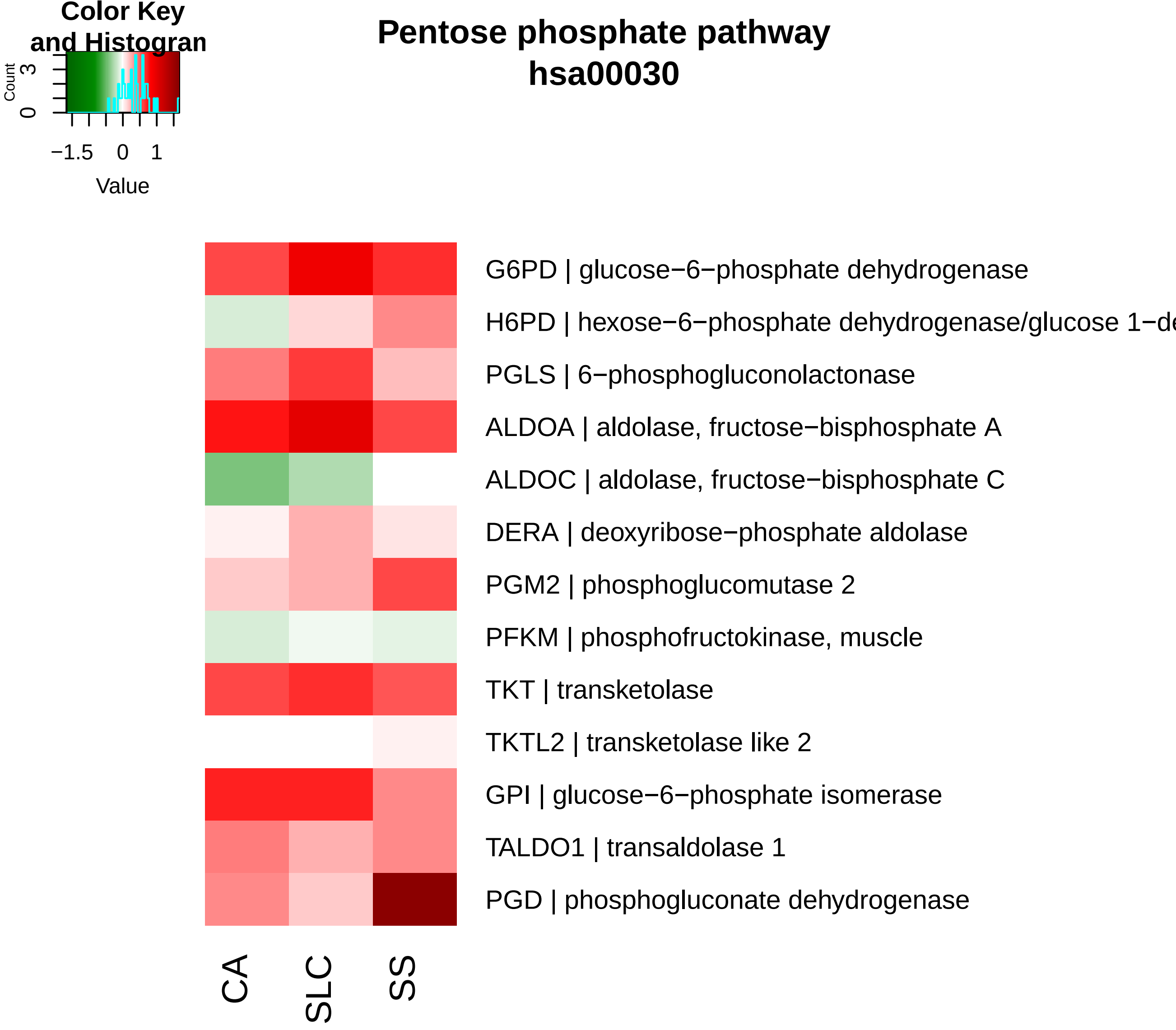

Carbohydrate metabolism

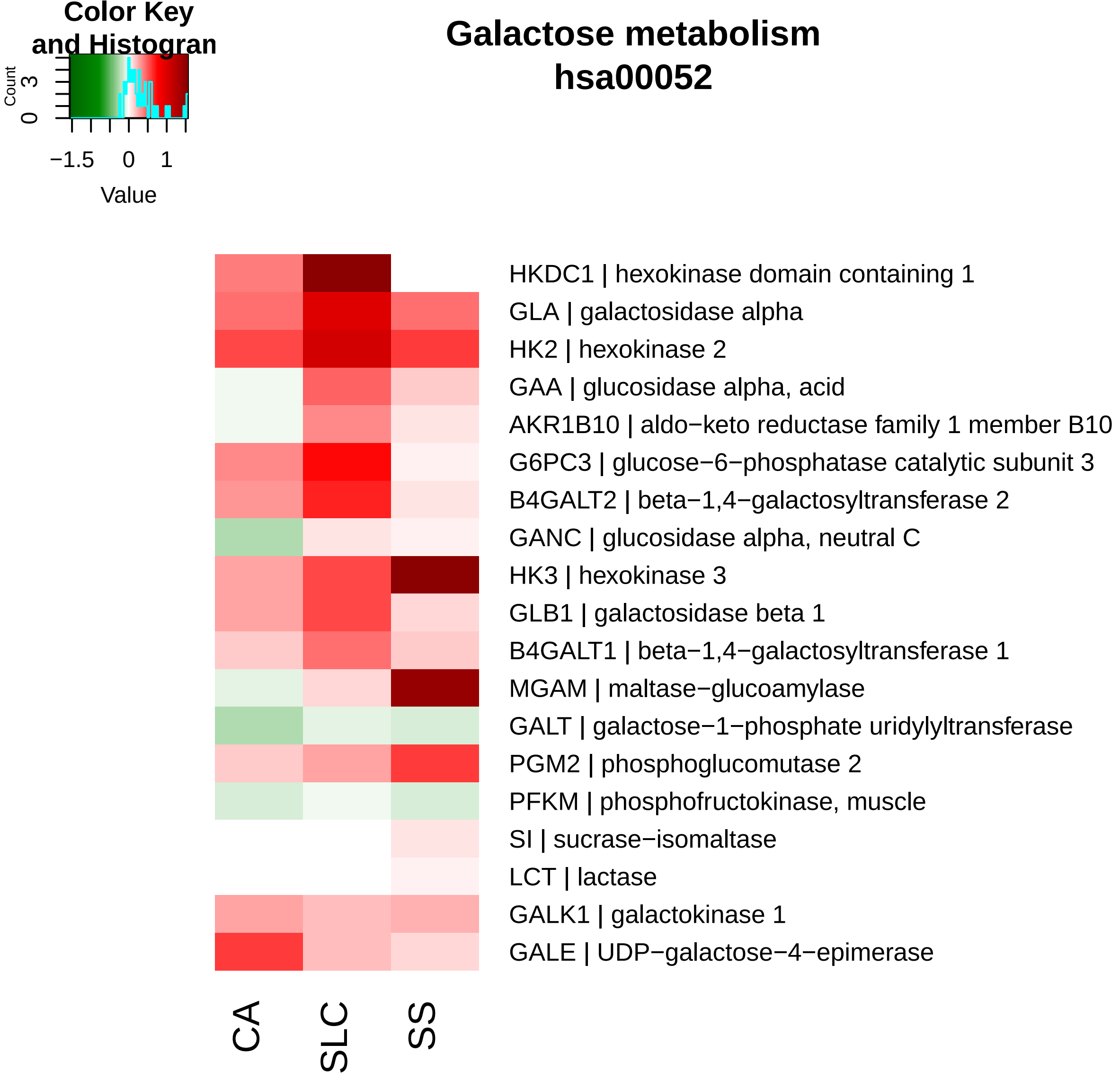

Carbohydrate metabolism

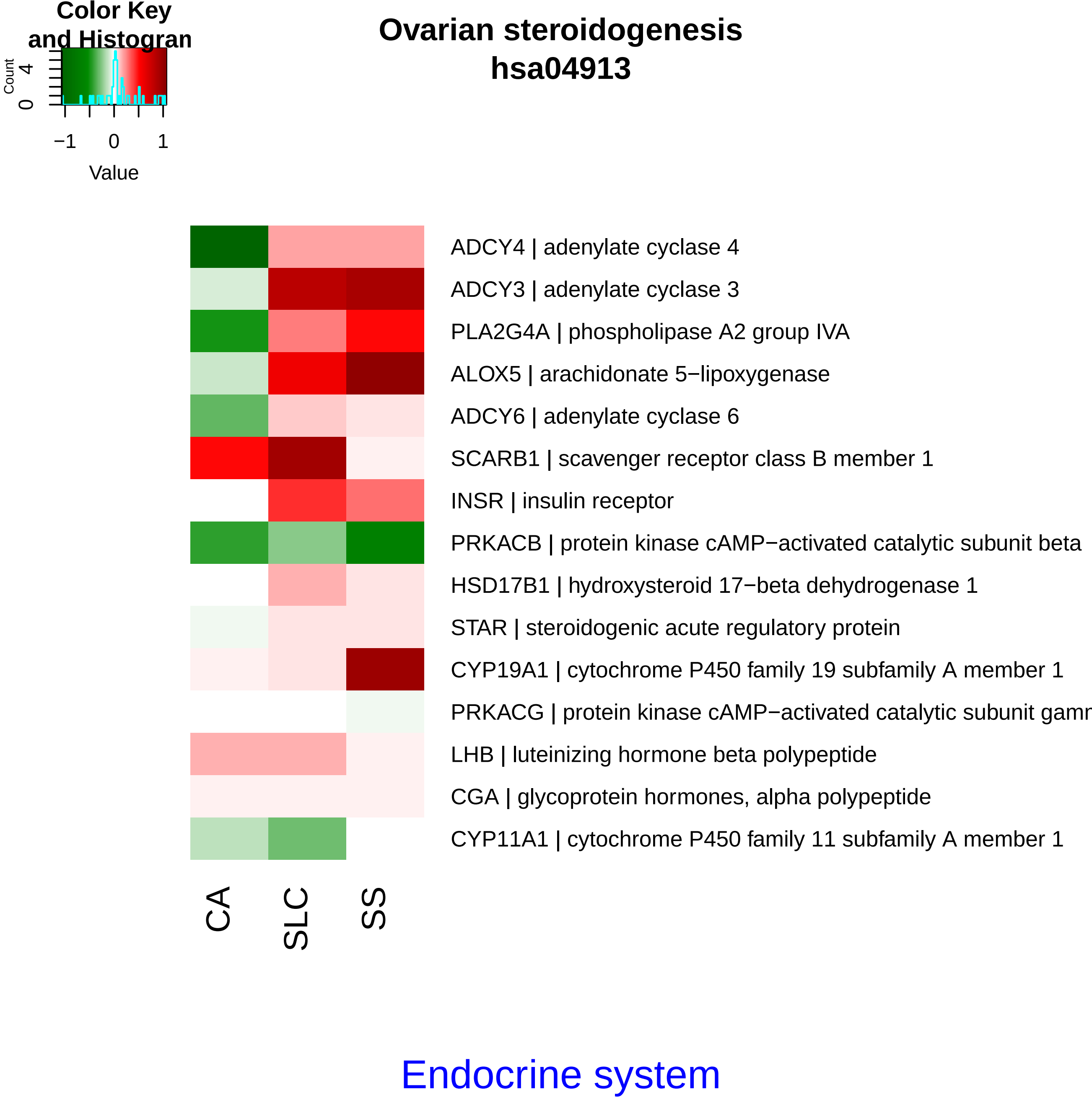
Color Key
and Histogran
Count
0
4
−1
0
1
Value
Ovarian steroidogenesis
hsa04913
ADCY4 | adenylate cyclase 4
ADCY3 | adenylate cyclase 3
PLA2G4A | phospholipase A2 group IVA
ALOX5 | arachidonate 5−lipoxygenase
ADCY6 | adenylate cyclase 6
SCARB1 | scavenger receptor class B member 1
INSR | insulin receptor
PRKACB | protein kinase cAMP−activated catalytic subunit beta
HSD17B1 | hydroxysteroid 17−beta dehydrogenase 1
STAR | steroidogenic acute regulatory protein
CYP19A1 | cytochrome P450 family 19 subfamily A member 1
PRKACG | protein kinase cAMP−activated catalytic subunit gamr
LHB | luteinizing hormone beta polypeptide
CGA | glycoprotein hormones, alpha polypeptide
CYP11A1 | cytochrome P450 family 11 subfamily A member 1
CA
SLC
SS
Endocrine system

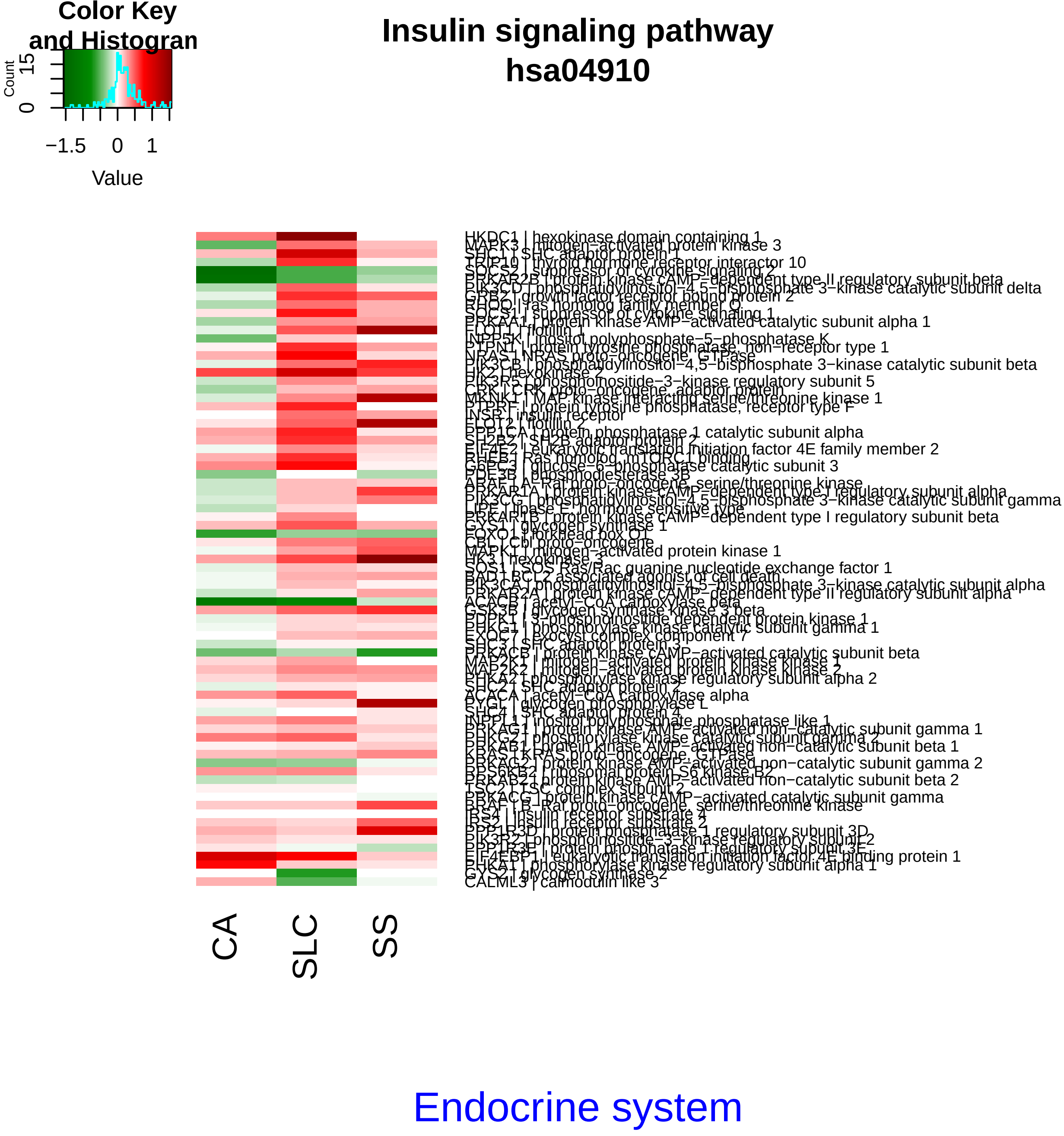
Color Key
and Histogran
Count
0
15
−1.5
0
1
Value
Insulin signaling pathway
hsa04910
HKDC1 | hexokinase domain containing 1
MAPK3 | mitogen−activated protein kinase 3
SHC1 | SHC adaptor protein 1
TRIP10 | thyroid hormone receptor interactor 10
SOCS2 | suppressor of cytokine signaling 2
PRKAR2B | protein kinase cAMP−dependent type II regulatory subunit beta
PIK3CD | phosphatidylinositol−4,5−bisphosphate 3−kinase catalytic subunit delta
GRB2 | growth factor receptor bound protein 2
RHOQ | ras homolog family member Q
SOCS1 | suppressor of cytokine signaling 1
PRKAA1 | protein kinase AMP−activated catalytic subunit alpha 1
FLOT1 | flotillin 1
INPP5K | inositol polyphosphate−5−phosphatase K
PTPN1 | protein tyrosine phosphatase, non−receptor type 1
NRAS | NRAS proto−oncogene, GTPase
PIK3CB | phosphatidylinositol−4,5−bisphosphate 3−kinase catalytic subunit beta
HK2 | hexokinase 2
PIK3R5 | phosphoinositide−3−kinase regulatory subunit 5
CRK | CRK proto−oncogene, adaptor protein
MKNK1 | MAP kinase interacting serine/threonine kinase 1
PTPRF | protein tyrosine phosphatase, receptor type F
INSR | insulin receptor
FLOT2 | flotillin 2
PPP1CA | protein phosphatase 1 catalytic subunit alpha
SH2B2 | SH2B adaptor protein 2
EIF4E2 | eukaryotic translation initiation factor 4E family member 2
RHEB | Ras homolog, mTORC1 binding
G6PC3 | glucose−6−phosphatase catalytic subunit 3
PDE3B | phosphodiesterase 3B
ARAF | A−Raf proto−oncogene, serine/threonine kinase
PRKAR1A | protein kinase cAMP−dependent type I regulatory subunit alpha
PIK3CG | phosphatidylinositol−4,5−bisphosphate 3−kinase catalytic subunit gamma
LIPE | lipase E, hormone sensitive type
PRKAR1B | protein kinase cAMP−dependent type I regulatory subunit beta
GYS1 | glycogen synthase 1
FOXO1 | forkhead box O1
CBL | Cbl proto−oncogene
MAPK1 | mitogen−activated protein kinase 1
HK3 | hexokinase 3
SOS1 | SOS Ras/Rac guanine nucleotide exchange factor 1
BAD | BCL2 associated agonist of cell death
PIK3CA | phosphatidylinositol−4,5−bisphosphate 3−kinase catalytic subunit alpha
PRKAR2A | protein kinase cAMP−dependent type II regulatory subunit alpha
ACACB | acetyl−CoA carboxylase beta
GSK3B | glycogen synthase kinase 3 beta
PDPK1 | 3−phosphoinositide dependent protein kinase 1
PHKG1 | phosphorylase kinase catalytic subunit gamma 1
EXOC7 | exocyst complex component 7
SHC3 | SHC adaptor protein 3
PRKACB | protein kinase cAMP−activated catalytic subunit beta
MAP2K1 | mitogen−activated protein kinase kinase 1
MAP2K2 | mitogen−activated protein kinase kinase 2
PHKA2 | phosphorylase kinase regulatory subunit alpha 2
SHC2 | SHC adaptor protein 2
ACACA | acetyl−CoA carboxylase alpha
PYGL | glycogen phosphorylase L
SHC4 | SHC adaptor protein 4
INPPL1 | inositol polyphosphate phosphatase like 1
PRKAG1 | protein kinase AMP−activated non−catalytic subunit gamma 1
PHKG2 | phosphorylase kinase catalytic subunit gamma 2
PRKAB1 | protein kinase AMP−activated non−catalytic subunit beta 1
KRAS | KRAS proto−oncogene, GTPase
PRKAG2 | protein kinase AMP−activated non−catalytic subunit gamma 2
RPS6KB2 | ribosomal protein S6 kinase B2
PRKAB2 | protein kinase AMP−activated non−catalytic subunit beta 2
TSC2 | TSC complex subunit 2
PRKACG | protein kinase cAMP−activated catalytic subunit gamma
BRAF | B−Raf proto−oncogene, serine/threonine kinase
IRS4 | insulin receptor substrate 4
IRS2 | insulin receptor substrate 2
PPP1R3D | protein phosphatase 1 regulatory subunit 3D
PIK3R2 | phosphoinositide−3−kinase regulatory subunit 2
PPP1R3E | protein phosphatase 1 regulatory subunit 3E
EIF4EBP1 | eukaryotic translation initiation factor 4E binding protein 1
PHKA1 | phosphorylase kinase regulatory subunit alpha 1
GYS2 | glycogen synthase 2
CALML3 | calmodulin like 3
CA
SLC
SS
Endocrine system

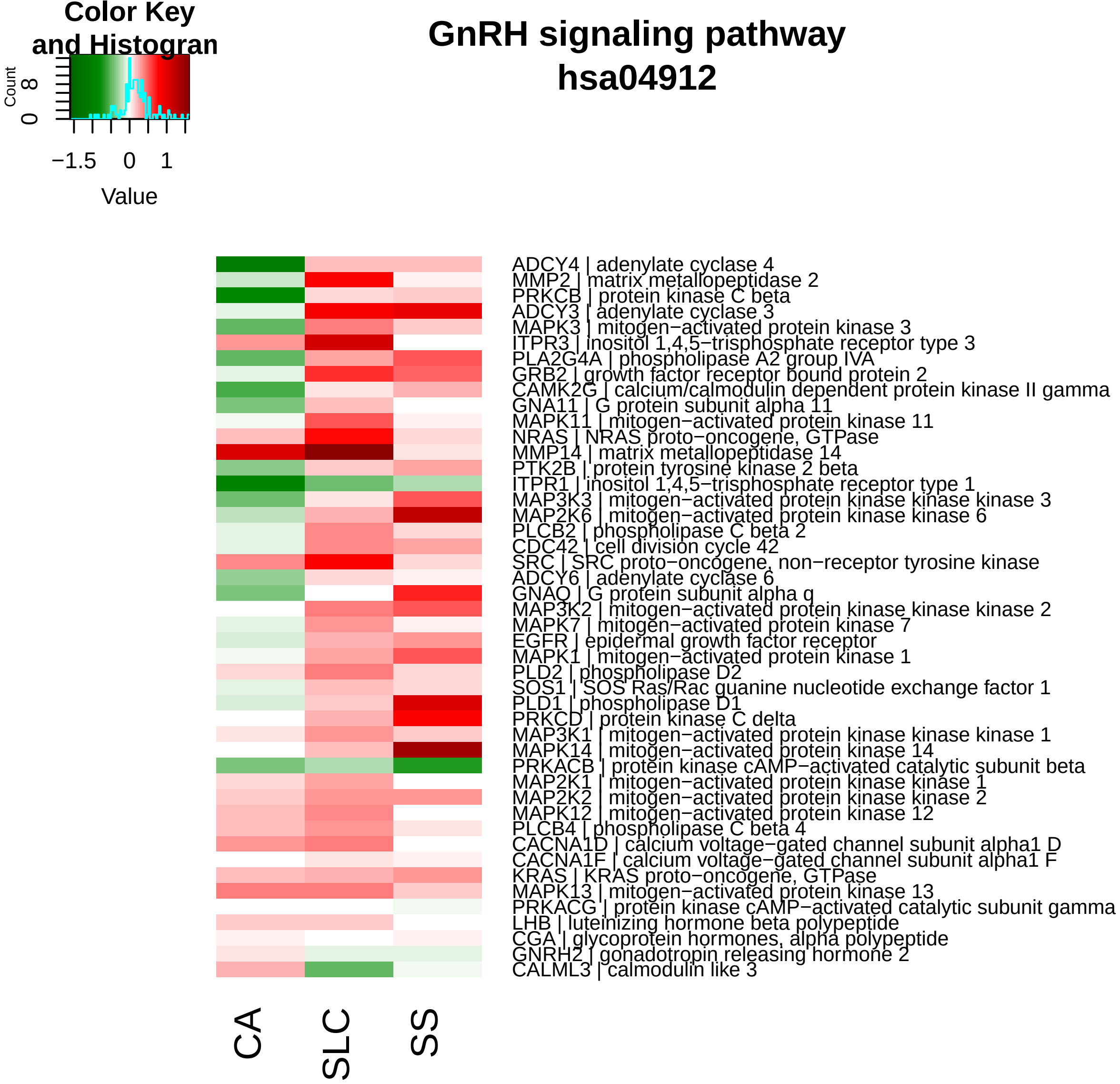

Endocrine system

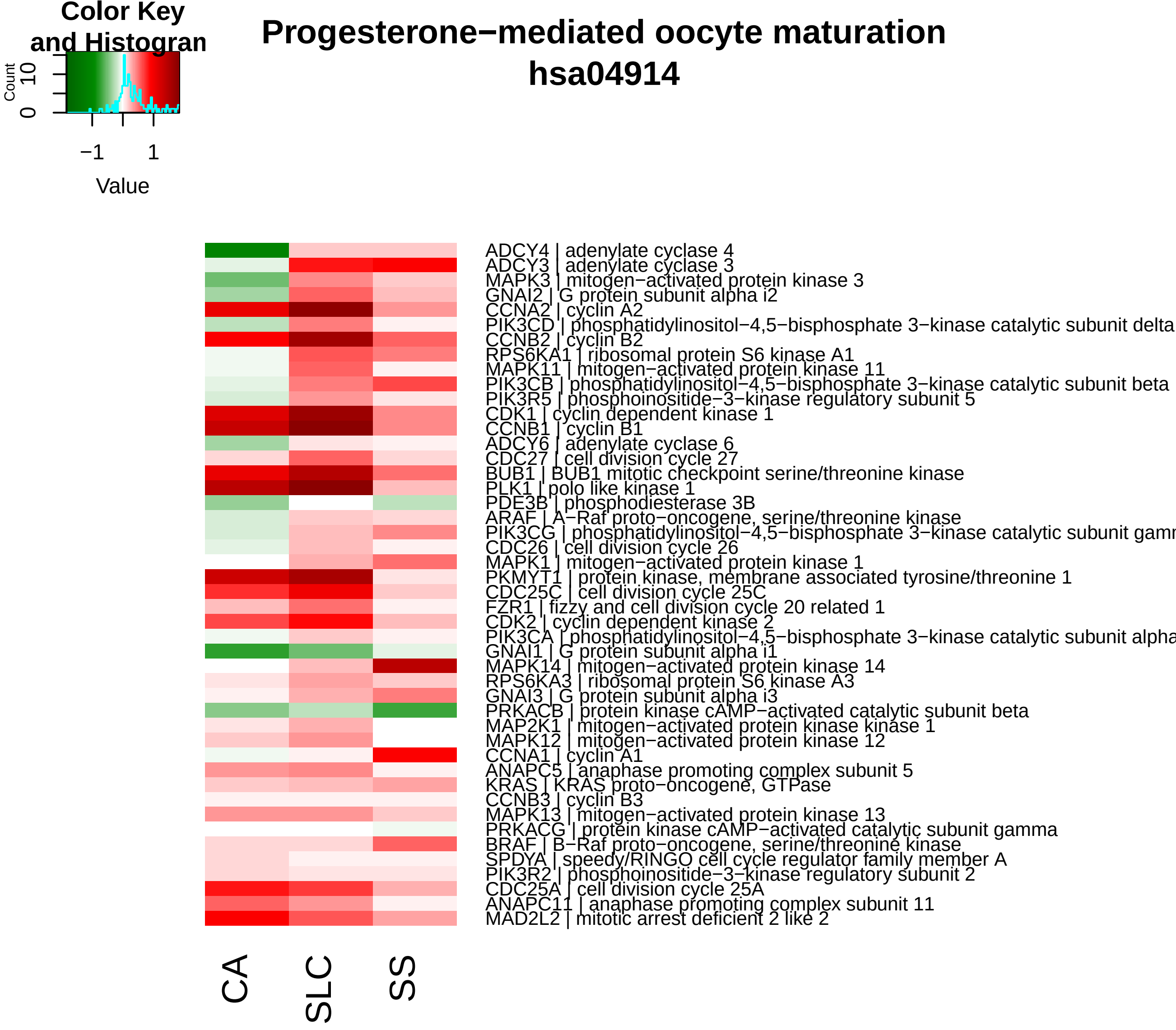

Endocrine system

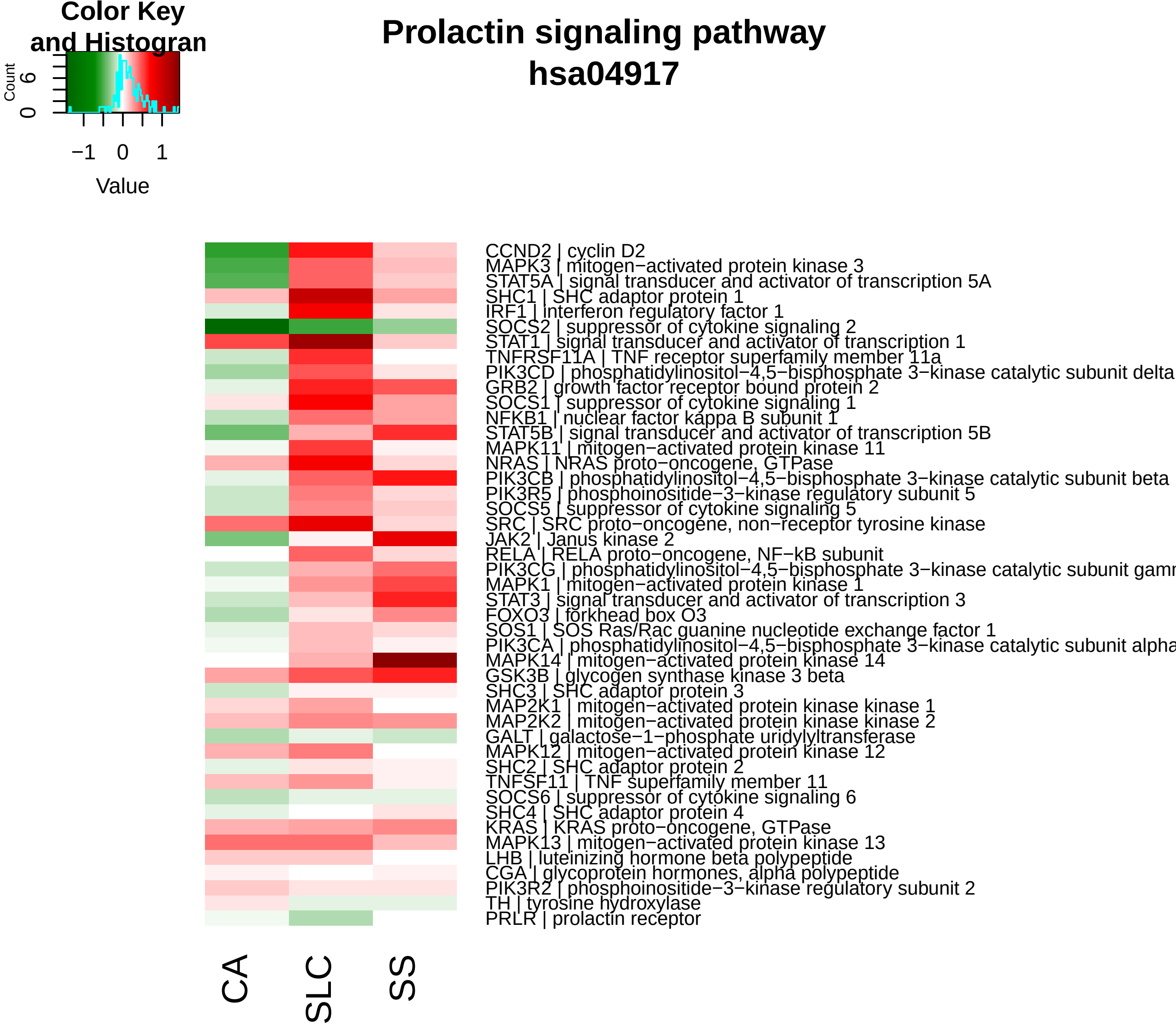

Endocrine system

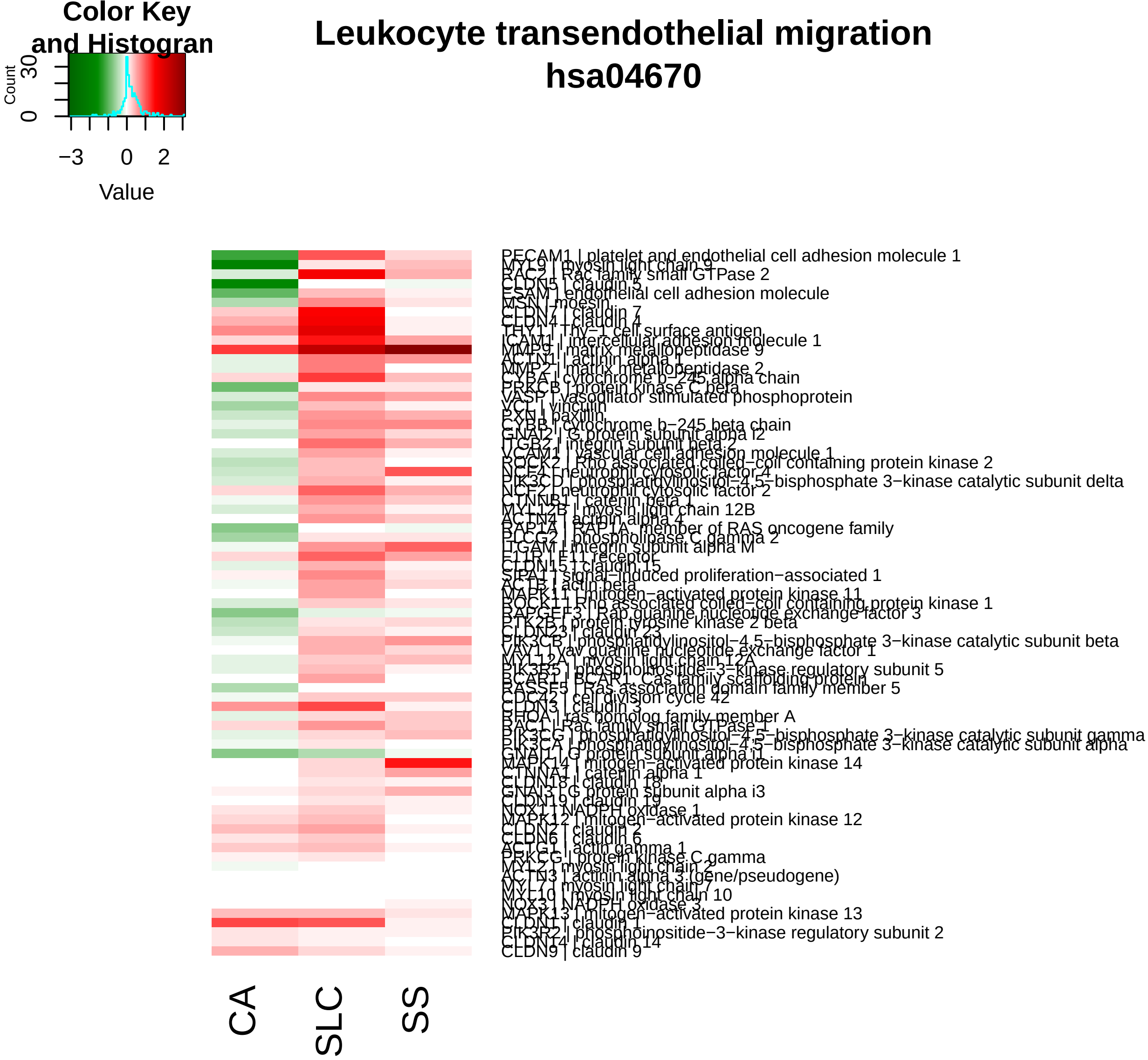

Immune system

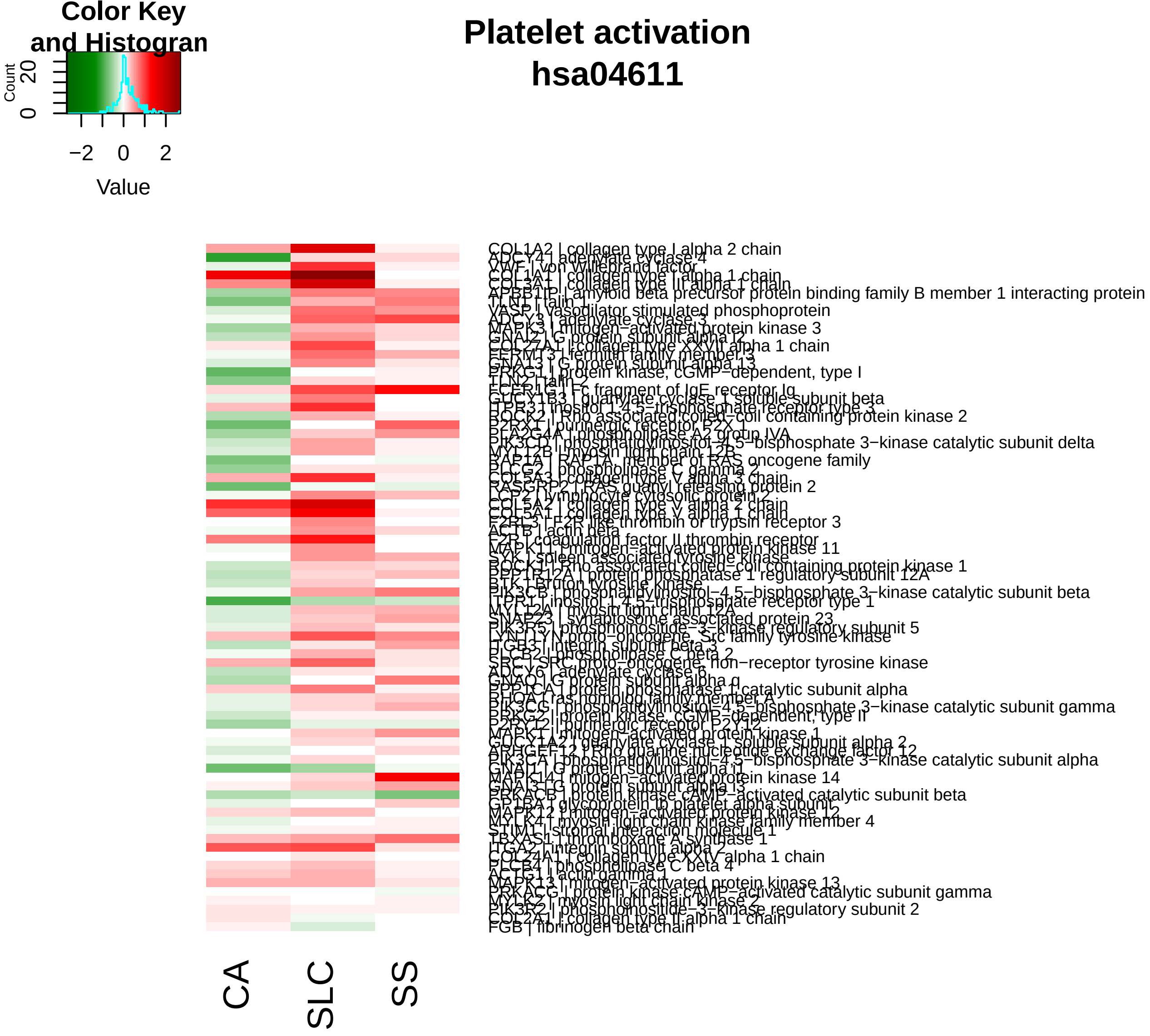

Immune system

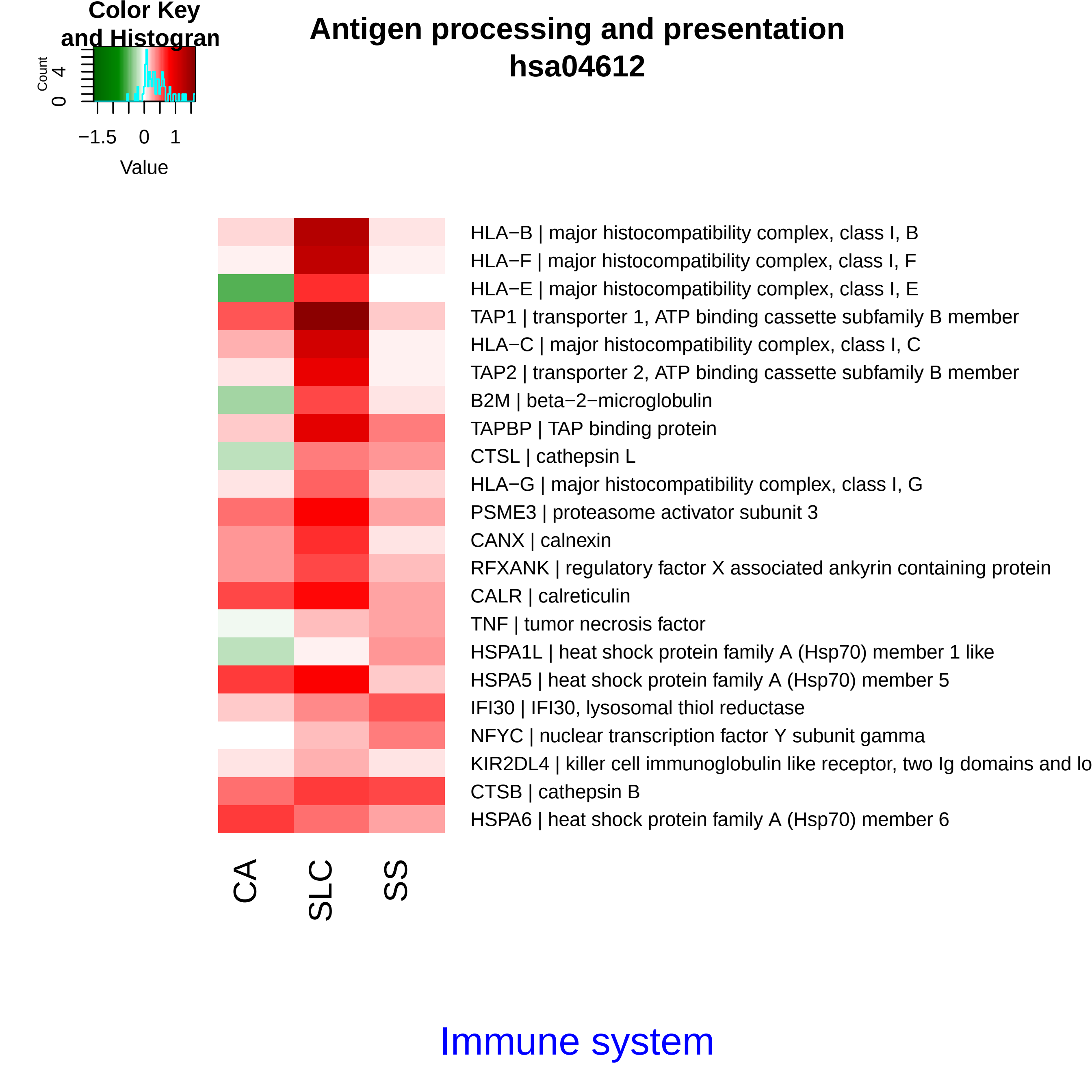
Color Key
and Histogran
Count
0
4
−1.5
0
1
Value
Antigen processing and presentation
hsa04612
HLA−B | major histocompatibility complex, class I, B
HLA−F | major histocompatibility complex, class I, F
HLA−E | major histocompatibility complex, class I, E
TAP1 | transporter 1, ATP binding cassette subfamily B member
HLA−C | major histocompatibility complex, class I, C
TAP2 | transporter 2, ATP binding cassette subfamily B member
B2M | beta−2−microglobulin
TAPBP | TAP binding protein
CTSL | cathepsin L
HLA−G | major histocompatibility complex, class I, G
PSME3 | proteasome activator subunit 3
CANX | calnexin
RFXANK | regulatory factor X associated ankyrin containing protein
CALR | calreticulin
TNF | tumor necrosis factor
HSPA1L | heat shock protein family A (Hsp70) member 1 like
HSPA5 | heat shock protein family A (Hsp70) member 5
IFI30 | IFI30, lysosomal thiol reductase
NFYC | nuclear transcription factor Y subunit gamma
KIR2DL4 | killer cell immunoglobulin like receptor, two Ig domains and lo
CTSB | cathepsin B
HSPA6 | heat shock protein family A (Hsp70) member 6
CA
SLC
SS
Immune system

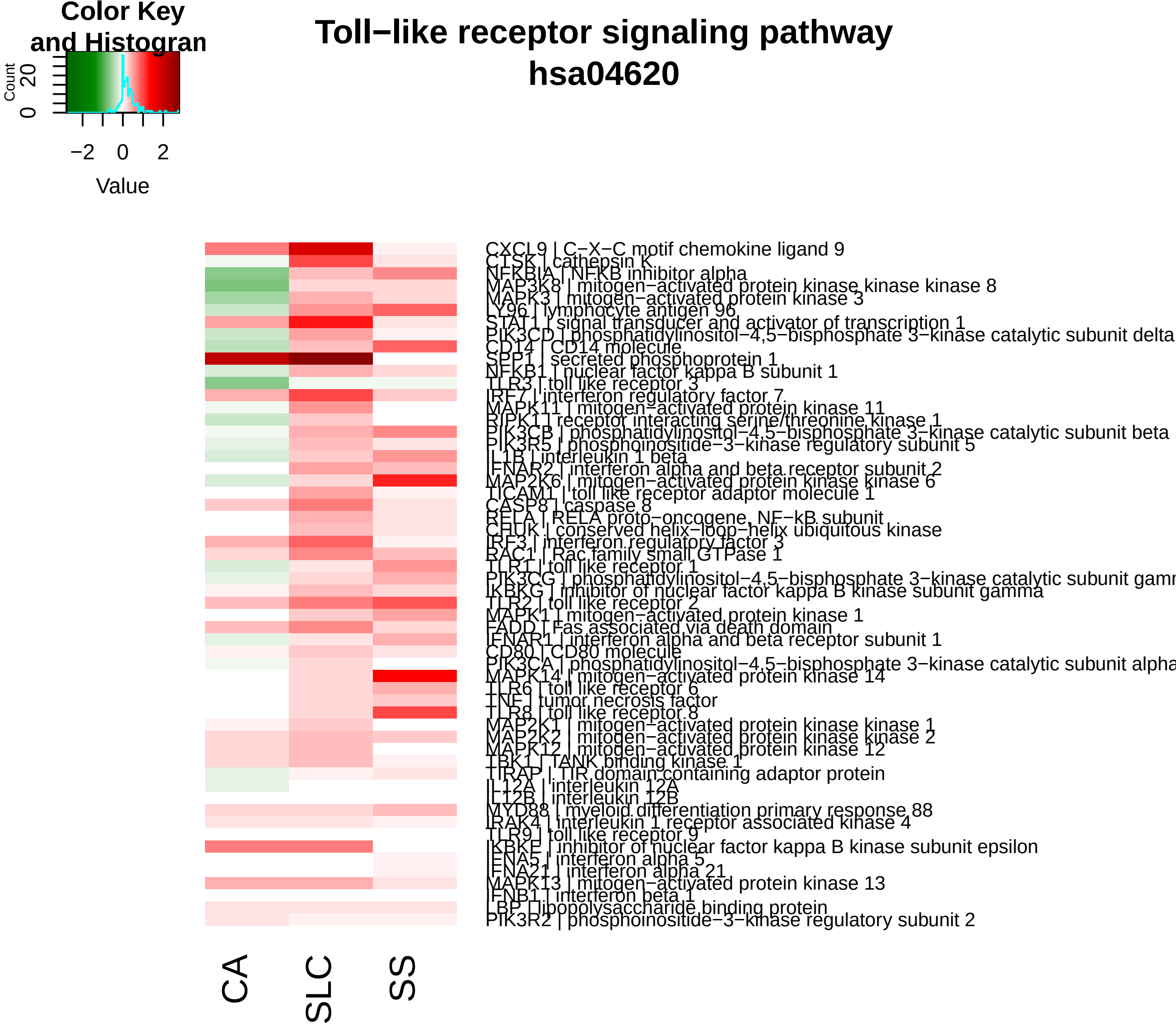

Immune system

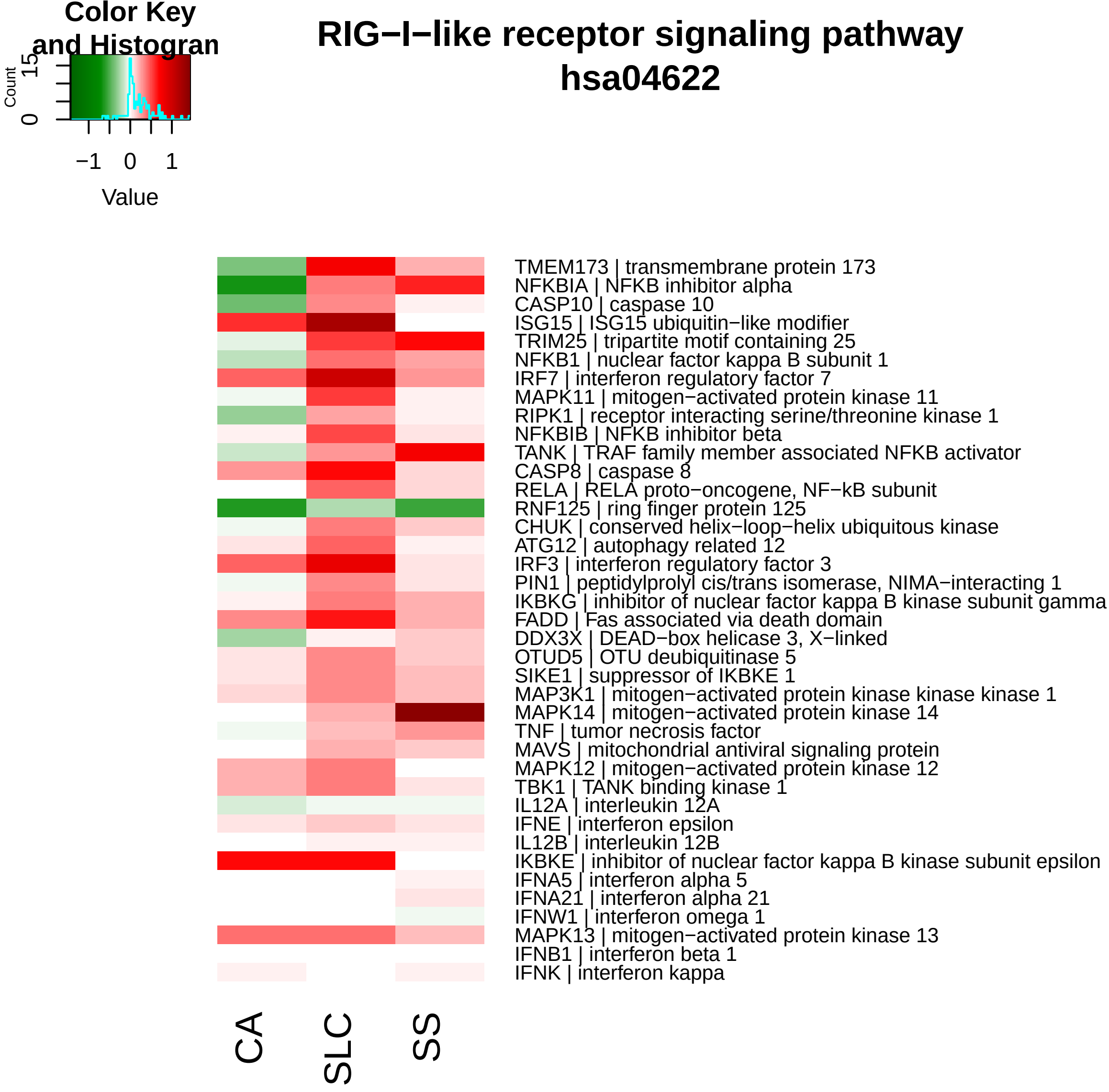

Immune system

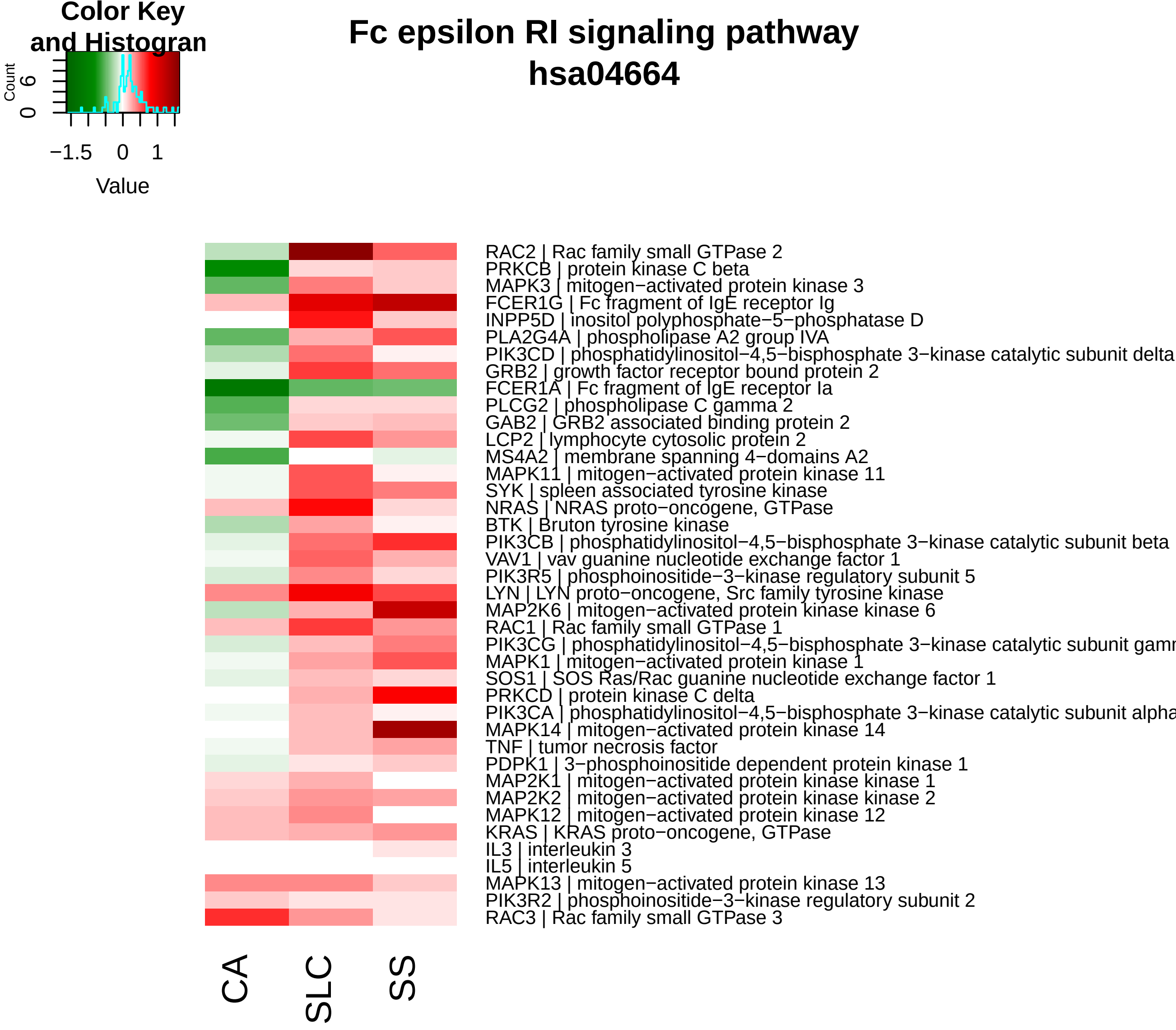

Immune system

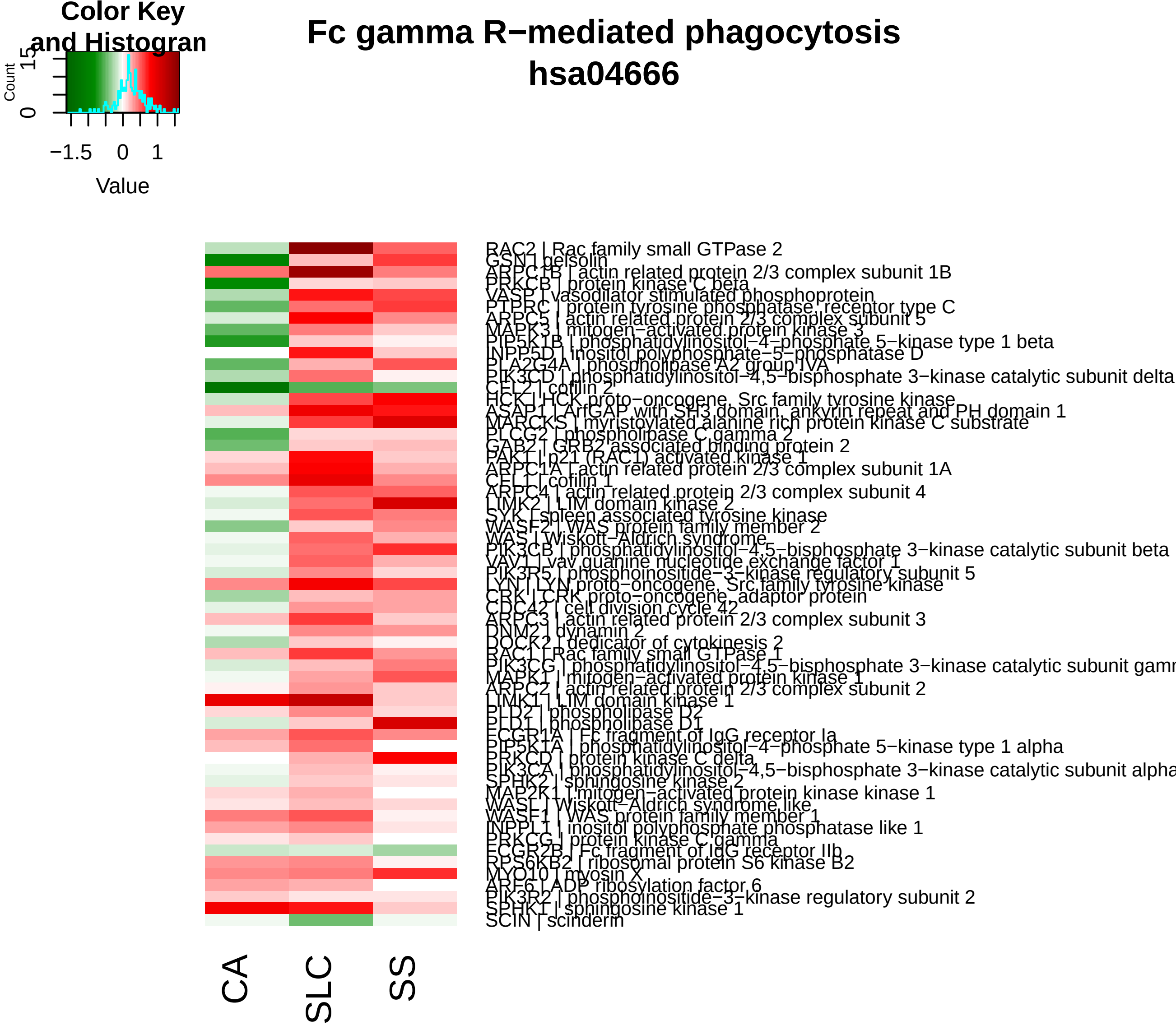

Immune system

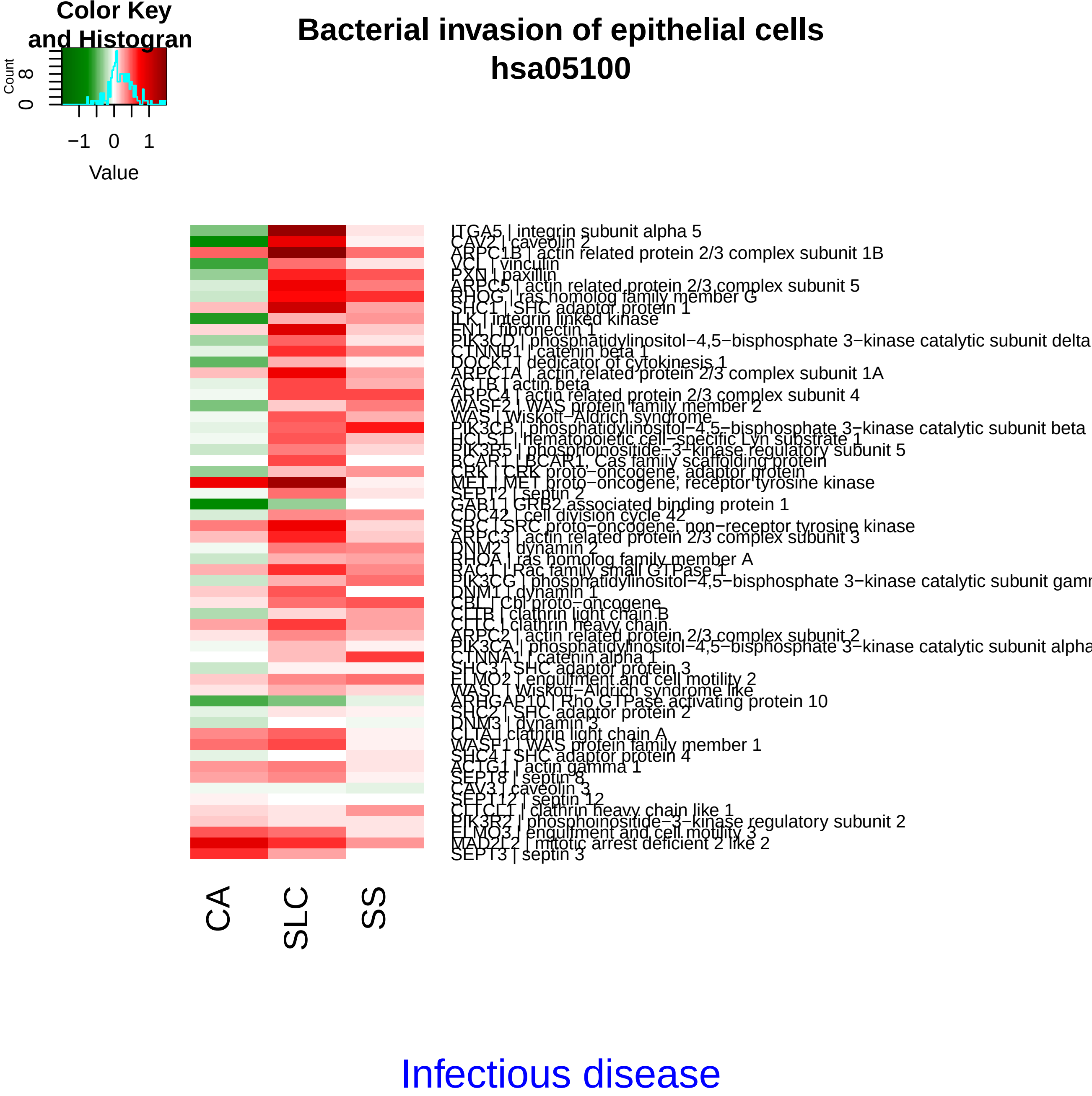
Color Key and Histogran
Count
0
8
−1
0
1
Value
Bacterial invasion of epithelial cells
hsa05100
ITGA5 | integrin subunit alpha 5
CAV2 | caveolin 2
ARPC1B | actin related protein 2/3 complex subunit 1B
VCL | vinculin
PXN | paxillin
ARPC5 | actin related protein 2/3 complex subunit 5
RHOG | ras homolog family member G
SHC1 | SHC adaptor protein 1
ILK | integrin linked kinase
FN1 | fibronectin 1
PIK3CD | phosphatidylinositol−4,5−bisphosphate 3−kinase catalytic subunit delta
CTNNB1 | catenin beta 1
DOCK1 | dedicator of cytokinesis 1
ARPC1A | actin related protein 2/3 complex subunit 1A
ACTB | actin beta
ARPC4 | actin related protein 2/3 complex subunit 4
WASF2 | WAS protein family member 2
WAS | Wiskott−Aldrich syndrome
PIK3CB | phosphatidylinositol−4,5−bisphosphate 3−kinase catalytic subunit beta
HCLS1 | hematopoietic cell−specific Lyn substrate 1
PIK3R5 | phosphoinositide−3−kinase regulatory subunit 5
BCAR1 | BCAR1, Cas family scaffolding protein
CRK | CRK proto−oncogene, adaptor protein
MET | MET proto−oncogene, receptor tyrosine kinase
SEPT2 | septin 2
GAB1 | GRB2 associated binding protein 1
CDC42 | cell division cycle 42
SRC | SRC proto−oncogene, non−receptor tyrosine kinase
ARPC3 | actin related protein 2/3 complex subunit 3
DNM2 | dynamin 2
RHOA | ras homolog family member A
RAC1 | Rac family small GTPase 1
PIK3CG | phosphatidylinositol−4,5−bisphosphate 3−kinase catalytic subunit gamr
DNM1 | dynamin 1
CBL | Cbl proto−oncogene
CLTB | clathrin light chain B
CLTC | clathrin heavy chain
ARPC2 | actin related protein 2/3 complex subunit 2
PIK3CA | phosphatidylinositol−4,5−bisphosphate 3−kinase catalytic subunit alpha
CTNNA1 | catenin alpha 1
SHC3 | SHC adaptor protein 3
ELMO2 | engulfment and cell motility 2
WASL | Wiskott−Aldrich syndrome like
ARHGAP10 | Rho GTPase activating protein 10
SHC2 | SHC adaptor protein 2
DNM3 | dynamin 3
CLTA | clathrin light chain A
WASF1 | WAS protein family member 1
SHC4 | SHC adaptor protein 4
ACTG1 | actin gamma 1
SEPT8 | septin 8
CAV3 | caveolin 3
SEPT12 | septin 12
CLTCL1 | clathrin heavy chain like 1
PIK3R2 | phosphoinositide−3−kinase regulatory subunit 2
ELMO3 | engulfment and cell motility 3
MAD2L2 | mitotic arrest deficient 2 like 2
SEPT3 | septin 3
CA
SLC
SS
Infectious disease

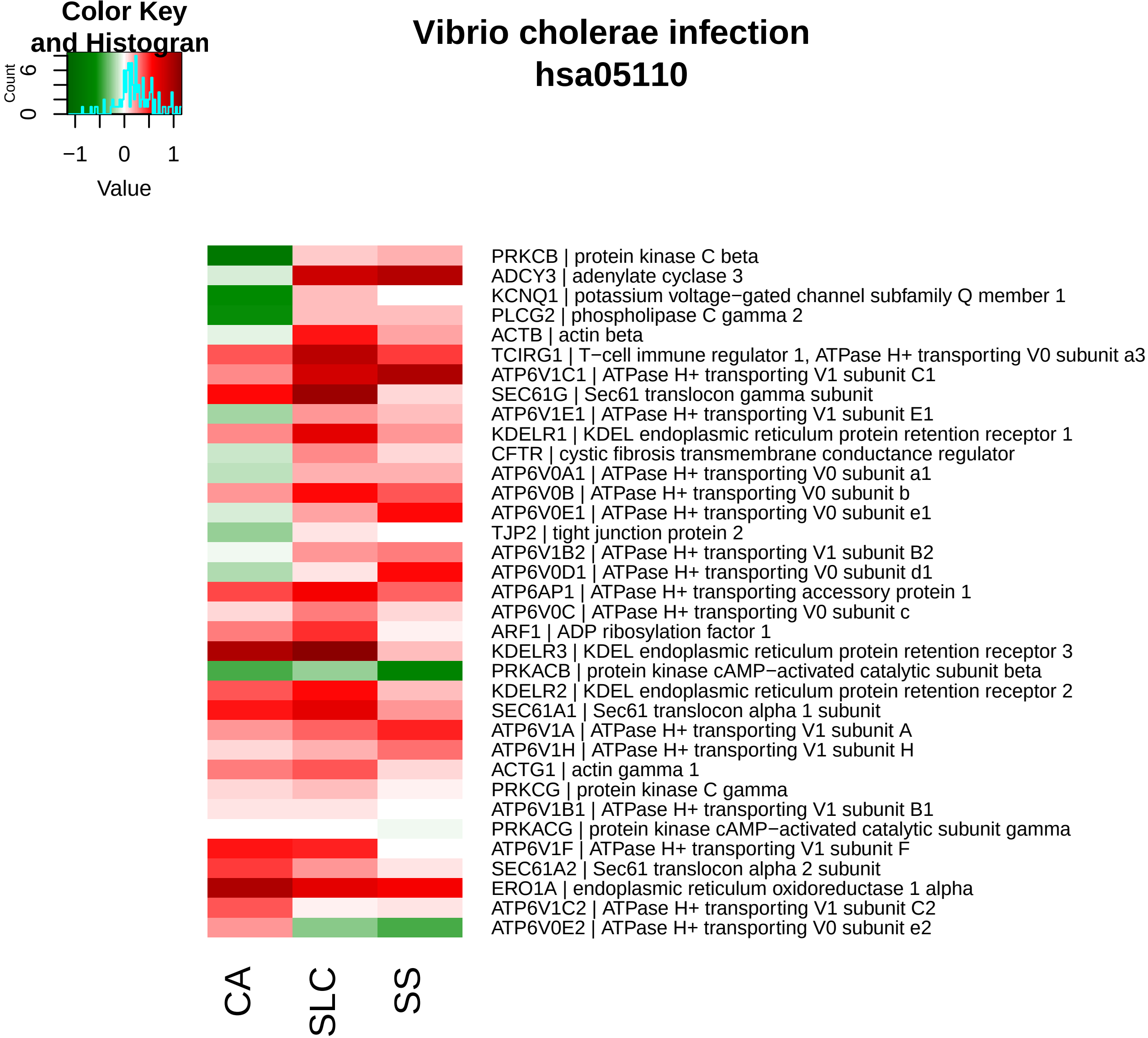

Infectious disease

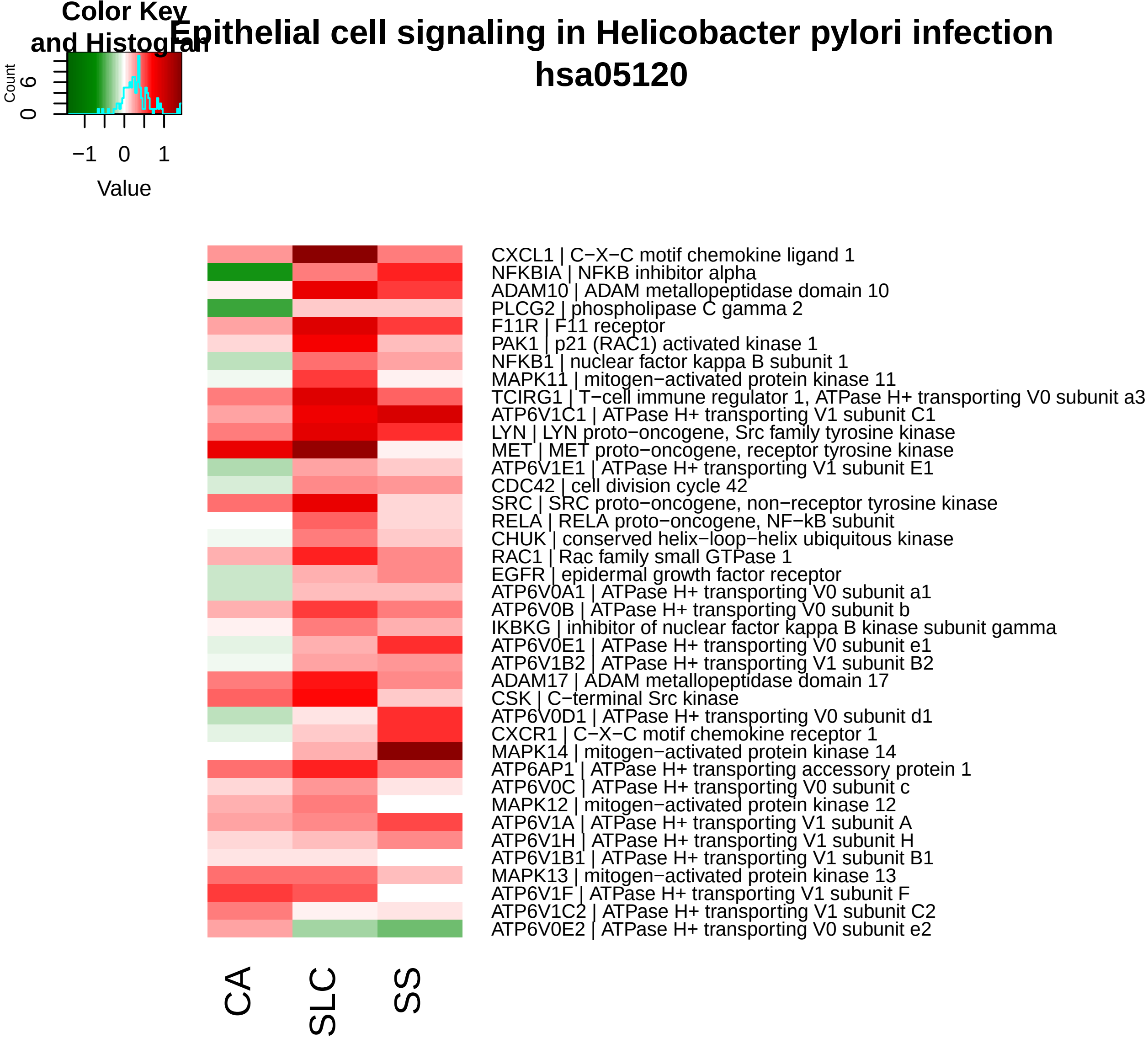

Infectious disease

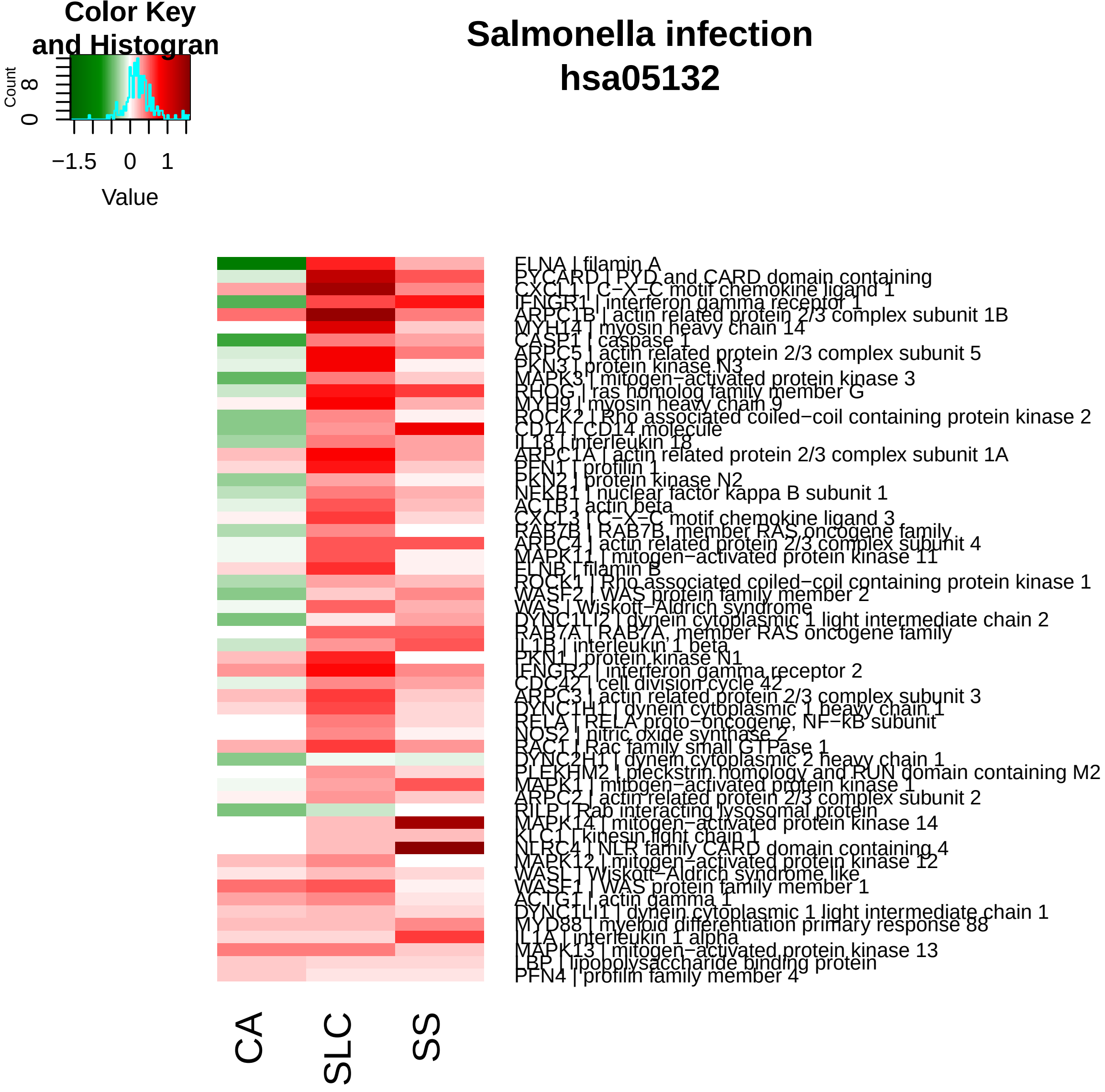

Infectious disease

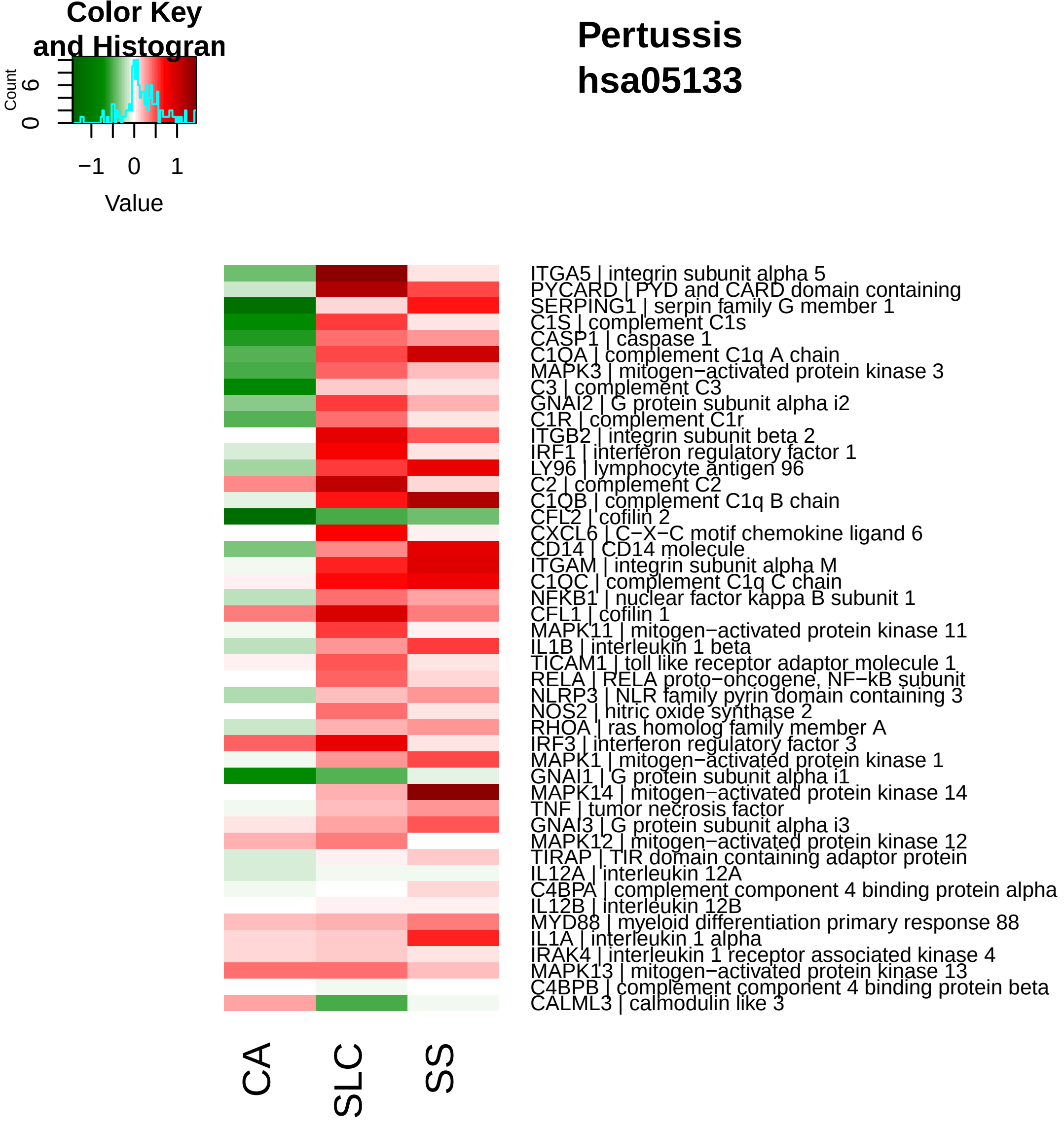

Infectious disease

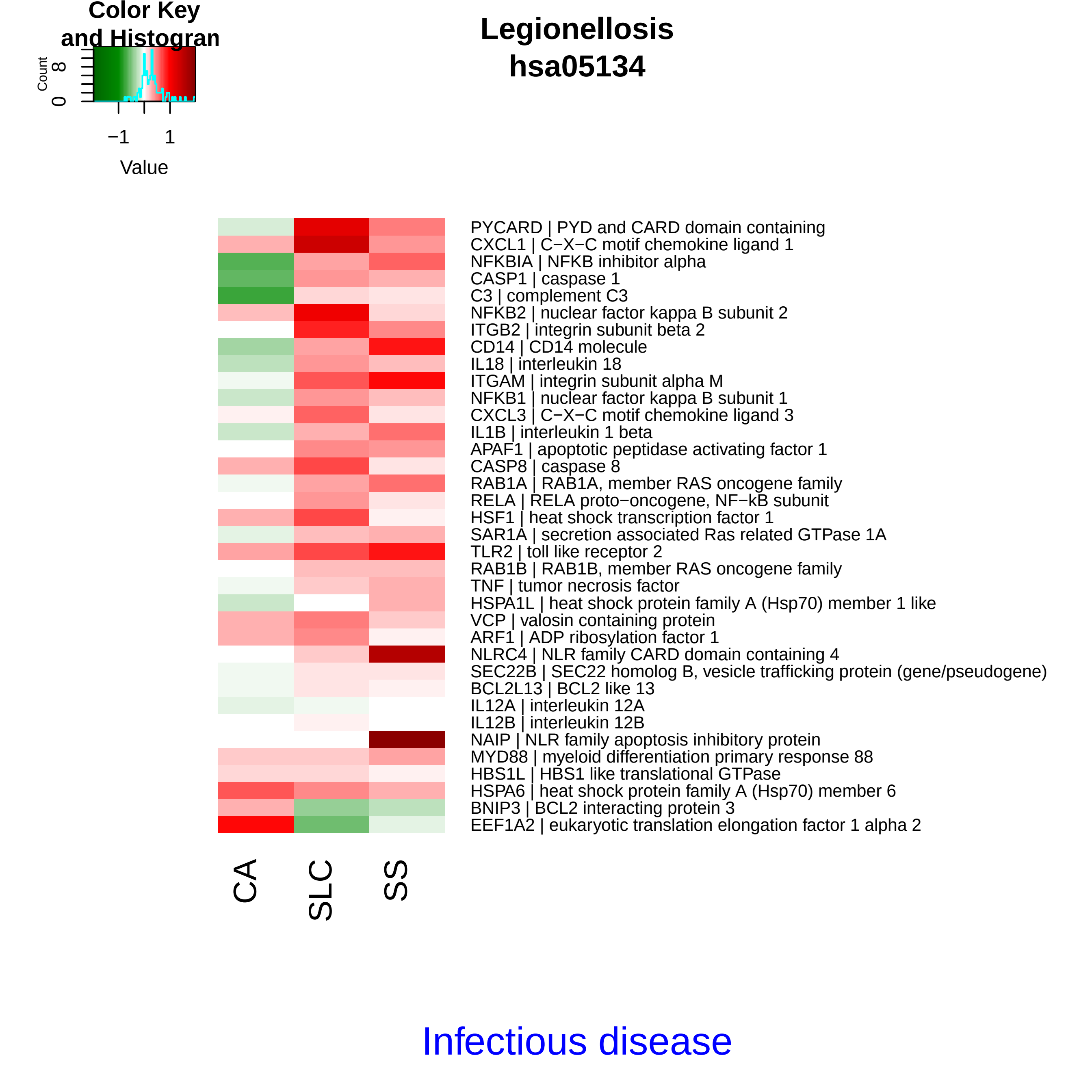
Color Key and Histogram
Count
0
8
−1
1
Value
Legionellosis
hsa05134
PYCARD | PYD and CARD domain containing
CXCL1 | C−X−C motif chemokine ligand 1
NFKBIA | NFKB inhibitor alpha
CASP1 | caspase 1
C3 | complement C3
NFKB2 | nuclear factor kappa B subunit 2
ITGB2 | integrin subunit beta 2
CD14 | CD14 molecule
IL18 | interleukin 18
ITGAM | integrin subunit alpha M
NFKB1 | nuclear factor kappa B subunit 1
CXCL3 | C−X−C motif chemokine ligand 3
IL1B | interleukin 1 beta
APAF1 | apoptotic peptidase activating factor 1
CASP8 | caspase 8
RAB1A | RAB1A, member RAS oncogene family
RELA | RELA proto−oncogene, NF−kB subunit
HSF1 | heat shock transcription factor 1
SAR1A | secretion associated Ras related GTPase 1A
TLR2 | toll like receptor 2
RAB1B | RAB1B, member RAS oncogene family
TNF | tumor necrosis factor
HSPA1L | heat shock protein family A (Hsp70) member 1 like
VCP | valosin containing protein
ARF1 | ADP ribosylation factor 1
NLRC4 | NLR family CARD domain containing 4
SEC22B | SEC22 homolog B, vesicle trafficking protein (gene/pseudogene)
BCL2L13 | BCL2 like 13
IL12A | interleukin 12A
IL12B | interleukin 12B
NAIP | NLR family apoptosis inhibitory protein
MYD88 | myeloid differentiation primary response 88
HBS1L | HBS1 like translational GTPase
HSPA6 | heat shock protein family A (Hsp70) member 6
BNIP3 | BCL2 interacting protein 3
EEF1A2 | eukaryotic translation elongation factor 1 alpha 2
CA
SLC
SS
Infectious disease

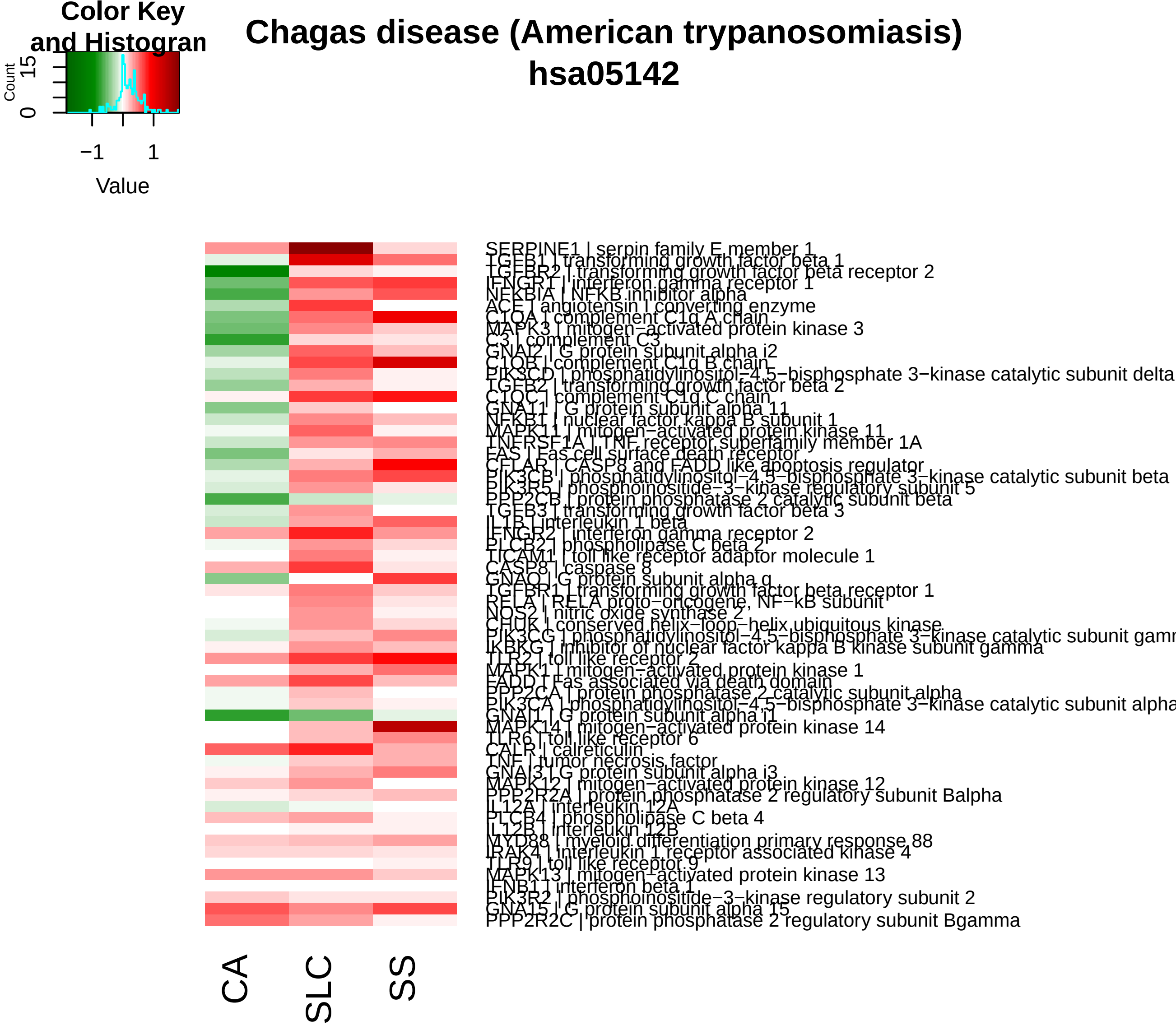

Infectious disease

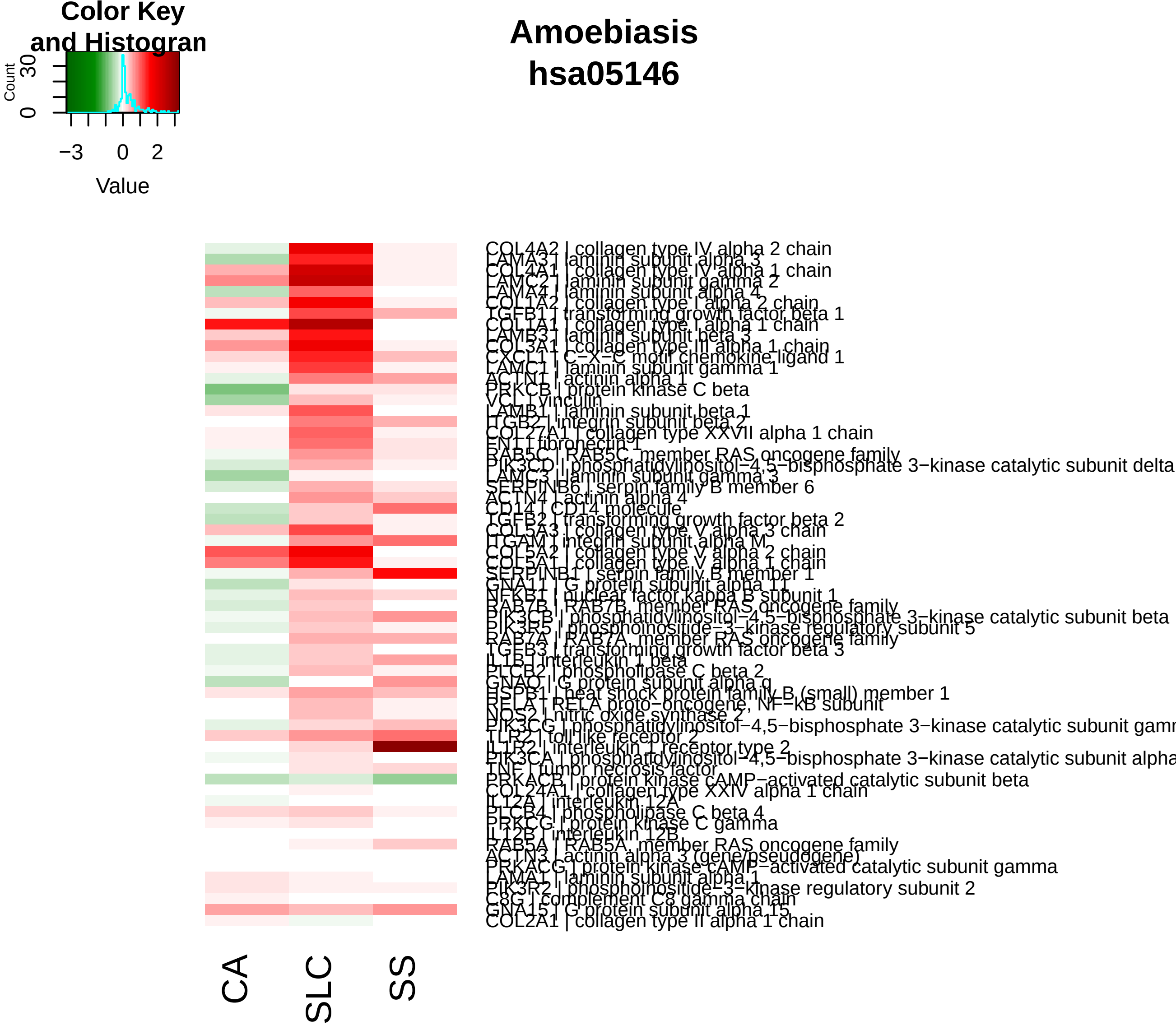

Infectious disease

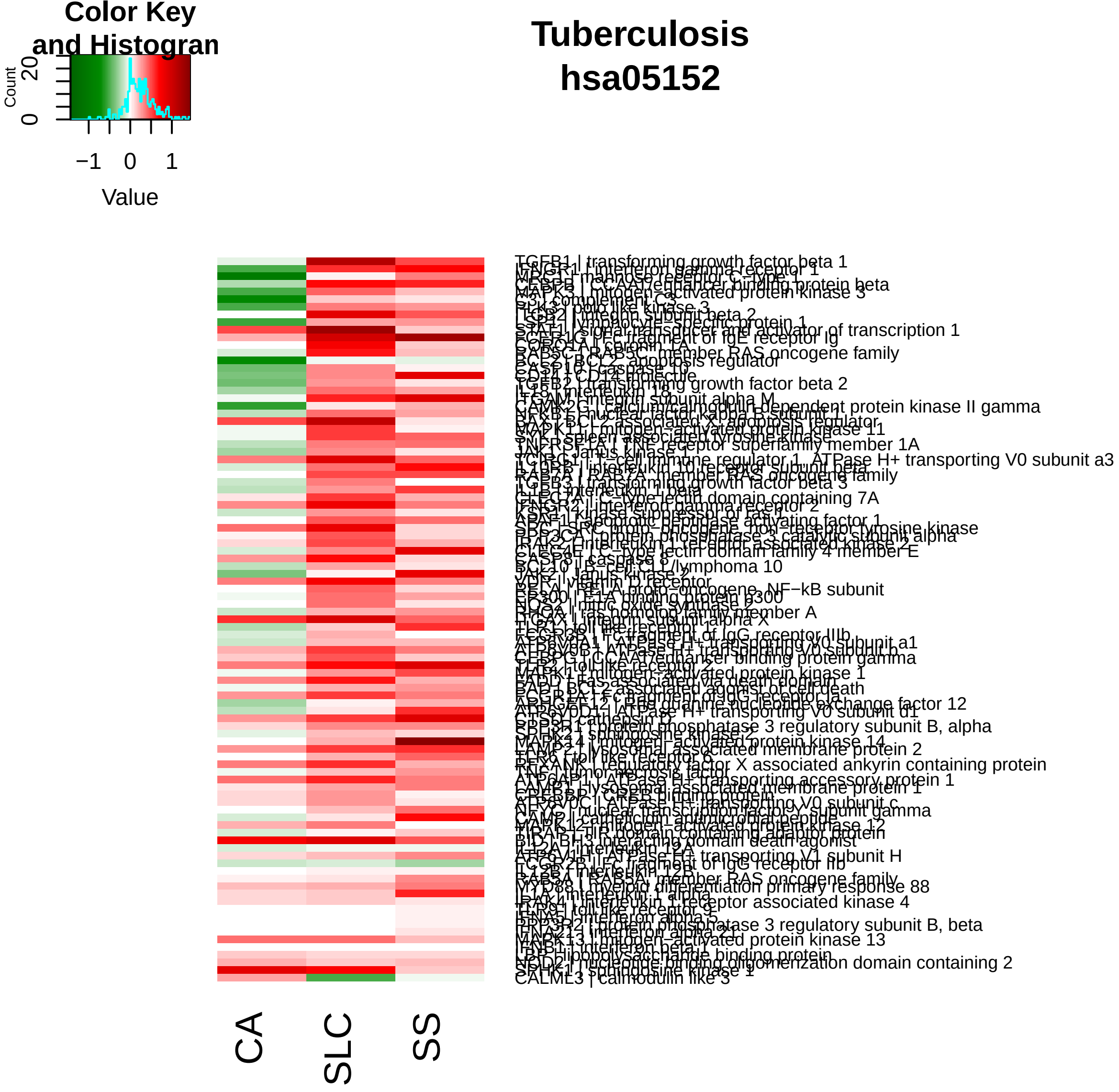

Infectious disease

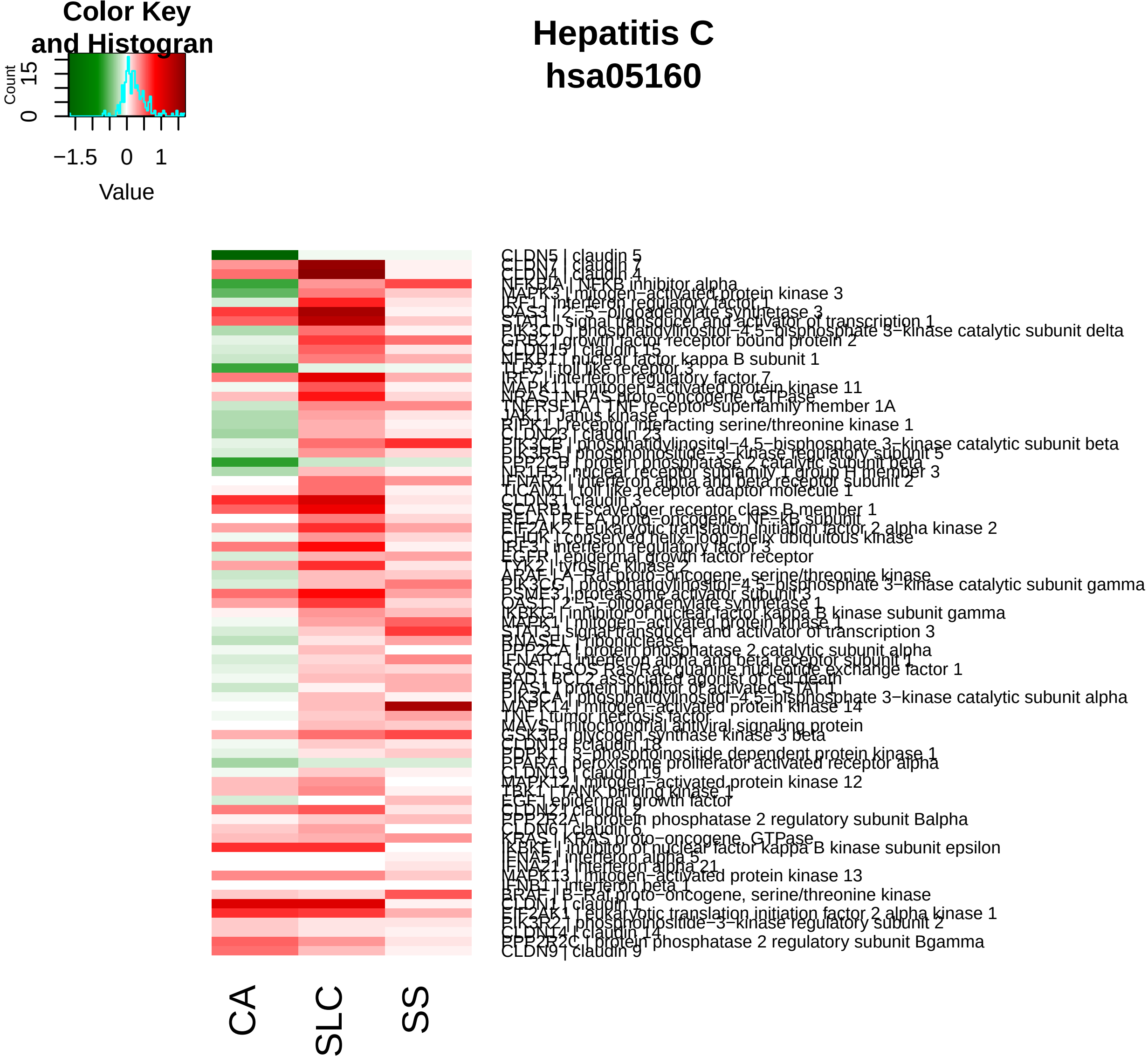

Infectious disease

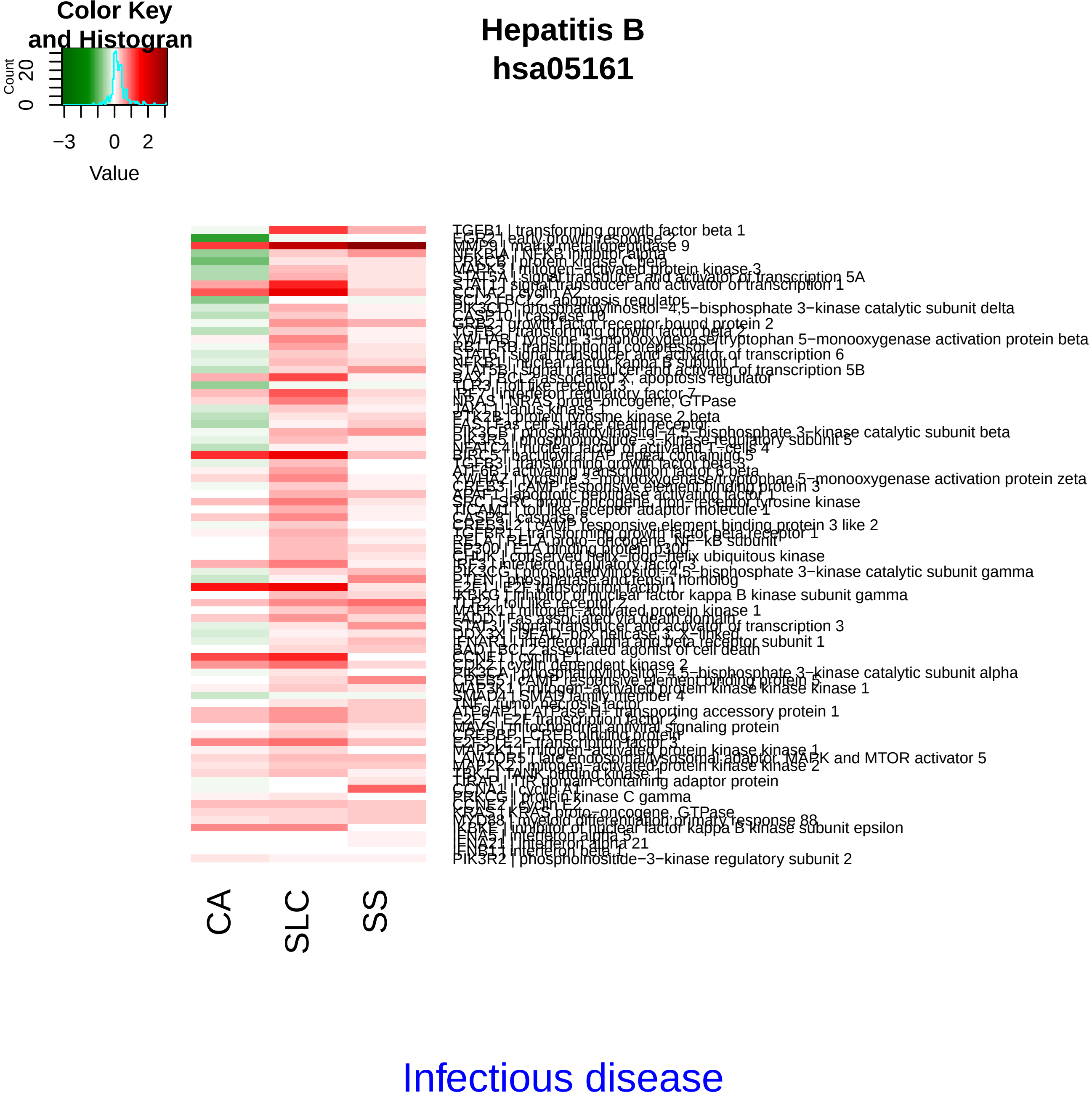
Color Key
and Histogram
Count
0
20
−3
0
2
Value
Hepatitis B
hsa05161
TGFB1 | transforming growth factor beta 1
EGR2 | early growth response 2
MMP9 | matrix metallopeptidase 9
NFKBIA | NFKB inhibitor alpha
PRKCB | protein kinase C beta
MAPK3 | mitogen−activated protein kinase 3
STAT5A | signal transducer and activator of transcription 5A
STAT1 | signal transducer and activator of transcription 1
CCNA2 | cyclin A2
BCL2 | BCL2, apoptosis regulator
PIK3CD | phosphatidylinositol−4,5−bisphosphate 3−kinase catalytic subunit delta
CASP10 | caspase 10
GRB2 | growth factor receptor bound protein 2
TGFB2 | transforming growth factor beta 2
YWHAB | tyrosine 3−monooxygenase/tryptophan 5−monooxygenase activation protein beta
RB1 | RB transcriptional corepressor 1
STAT6 | signal transducer and activator of transcription 6
NFKB1 | nuclear factor kappa B subunit 1
STAT5B | signal transducer and activator of transcription 5B
BAX | BCL2 associated X, apoptosis regulator
TLR3 | toll like receptor 3
IRF7 | interferon regulatory factor 7
NRAS | NRAS proto−oncogene, GTPase
JAK1 | Janus kinase 1
PTK2B | protein tyrosine kinase 2 beta
FAS | Fas cell surface death receptor
PIK3CB | phosphatidylinositol−4,5−bisphosphate 3−kinase catalytic subunit beta
PIK3R5 | phosphoinositide−3−kinase regulatory subunit 5
NFATC4 | nuclear factor of activated T−cells 4
BIRC5 | baculoviral IAP repeat containing 5
TGFB3 | transforming growth factor beta 3
ATF6B | activating transcription factor 6 beta
YWHAZ | tyrosine 3−monooxygenase/tryptophan 5−monooxygenase activation protein zeta
CREB3 | cAMP responsive element binding protein 3
APAF1 | apoptotic peptidase activating factor 1
SRC | SRC proto−oncogene, non−receptor tyrosine kinase
TICAM1 | toll like receptor adaptor molecule 1
CASP8 | caspase 8
CREB3L2 | cAMP responsive element binding protein 3 like 2
TGFBR1 | transforming growth factor beta receptor 1
RELA | RELA proto−oncogene, NF−kB subunit
EP300 | E1A binding protein p300
CHUK | conserved helix−loop−helix ubiquitous kinase
IRF3 | interferon regulatory factor 3
PIK3CG | phosphatidylinositol−4,5−bisphosphate 3−kinase catalytic subunit gamma
PTEN | phosphatase and tensin homolog
E2F1 | E2F transcription factor 1
IKBKG | inhibitor of nuclear factor kappa B kinase subunit gamma
TLR2 | toll like receptor 2
MAPK1 | mitogen−activated protein kinase 1
FADD | Fas associated via death domain
STAT3 | signal transducer and activator of transcription 3
DDX3X | DEAD−box helicase 3, X−linked
IFNAR1 | interferon alpha and beta receptor subunit 1
BAD | BCL2 associated agonist of cell death
CCNE1 | cyclin E1
CDK2 | cyclin dependent kinase 2
PIK3CA | phosphatidylinositol−4,5−bisphosphate 3−kinase catalytic subunit alpha
CREB5 | cAMP responsive element binding protein 5
MAP3K1 | mitogen−activated protein kinase kinase kinase 1
SMAD4 | SMAD family member 4
TNF | tumor necrosis factor
ATP6AP1 | ATPase H+ transporting accessory protein 1
E2F2 | E2F transcription factor 2
MAVS | mitochondrial antiviral signaling protein
CREBBP | CREB binding protein
E2F3 | E2F transcription factor 3
MAP2K1 | mitogen−activated protein kinase kinase 1
LAMTOR5 | late endosomal/lysosomal adaptor, MAPK and MTOR activator 5
MAP2K2 | mitogen−activated protein kinase kinase 2
TBK1 | TANK binding kinase 1
TIRAP | TIR domain containing adaptor protein
CCNA1 | cyclin A1
PRKCG | protein kinase C gamma
CCNE2 | cyclin E2
KRAS | KRAS proto−oncogene, GTPase
MYD88 | myeloid differentiation primary response 88
IKBKE | inhibitor of nuclear factor kappa B kinase subunit epsilon
IFNA5 | interferon alpha 5
IFNA21 | interferon alpha 21
IFNB1 | interferon beta 1
PIK3R2 | phosphoinositide−3−kinase regulatory subunit 2
CA
SLC
SS
Infectious disease

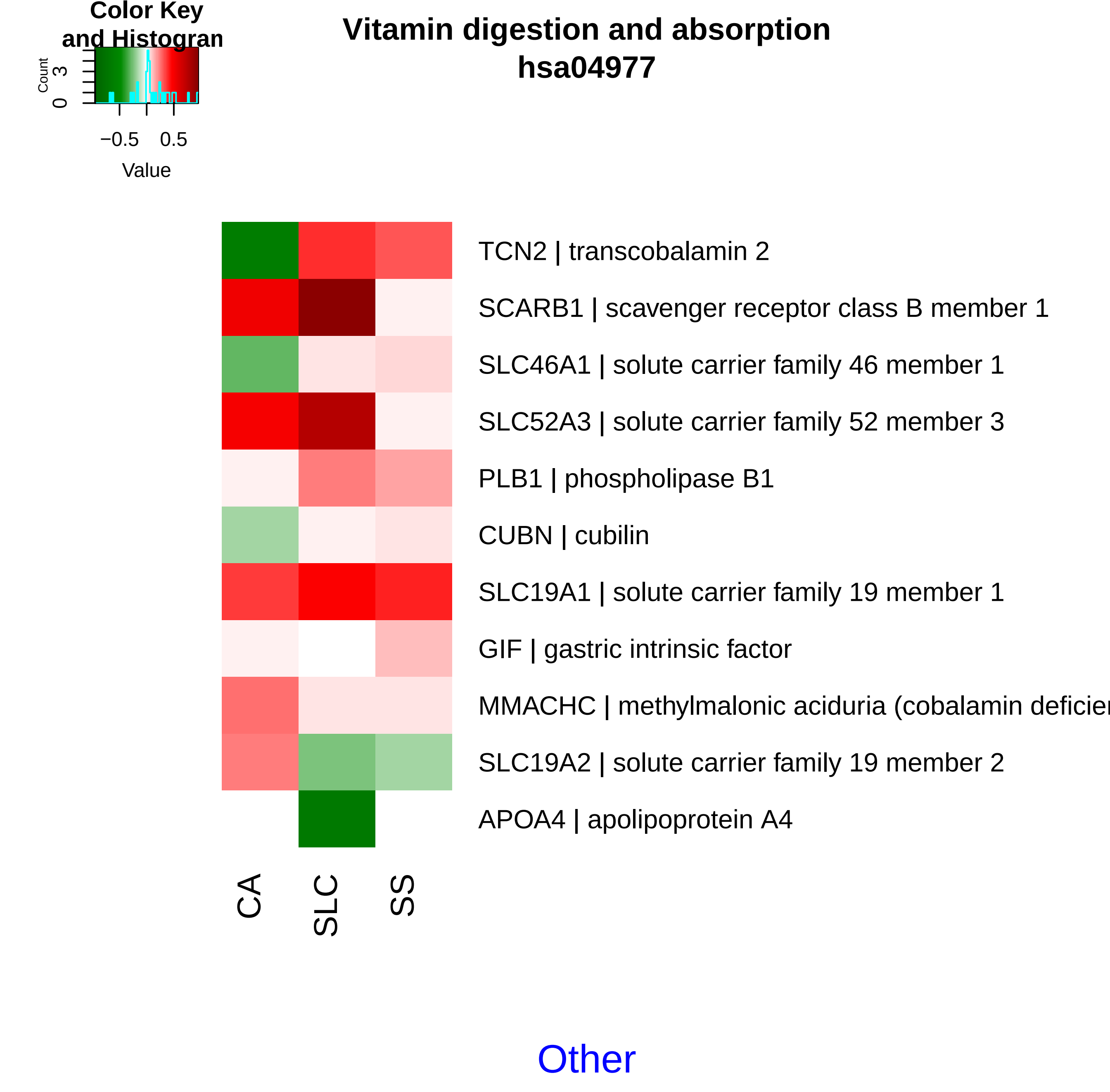
Color Key
and Histogran
Count
0
3
−0.5
0.5
Value
Vitamin digestion and absorption
hsa04977
TCN2 | transcobalamin 2
SCARB1 | scavenger receptor class B member 1
SLC46A1 | solute carrier family 46 member 1
SLC52A3 | solute carrier family 52 member 3
PLB1 | phospholipase B1
CUBN | cubilin
SLC19A1 | solute carrier family 19 member 1
GIF | gastric intrinsic factor
MMACHC | methylmalonic aciduria (cobalamin deficie
SLC19A2 | solute carrier family 19 member 2
APOA4 | apolipoprotein A4
CA
SLC
SS
Other

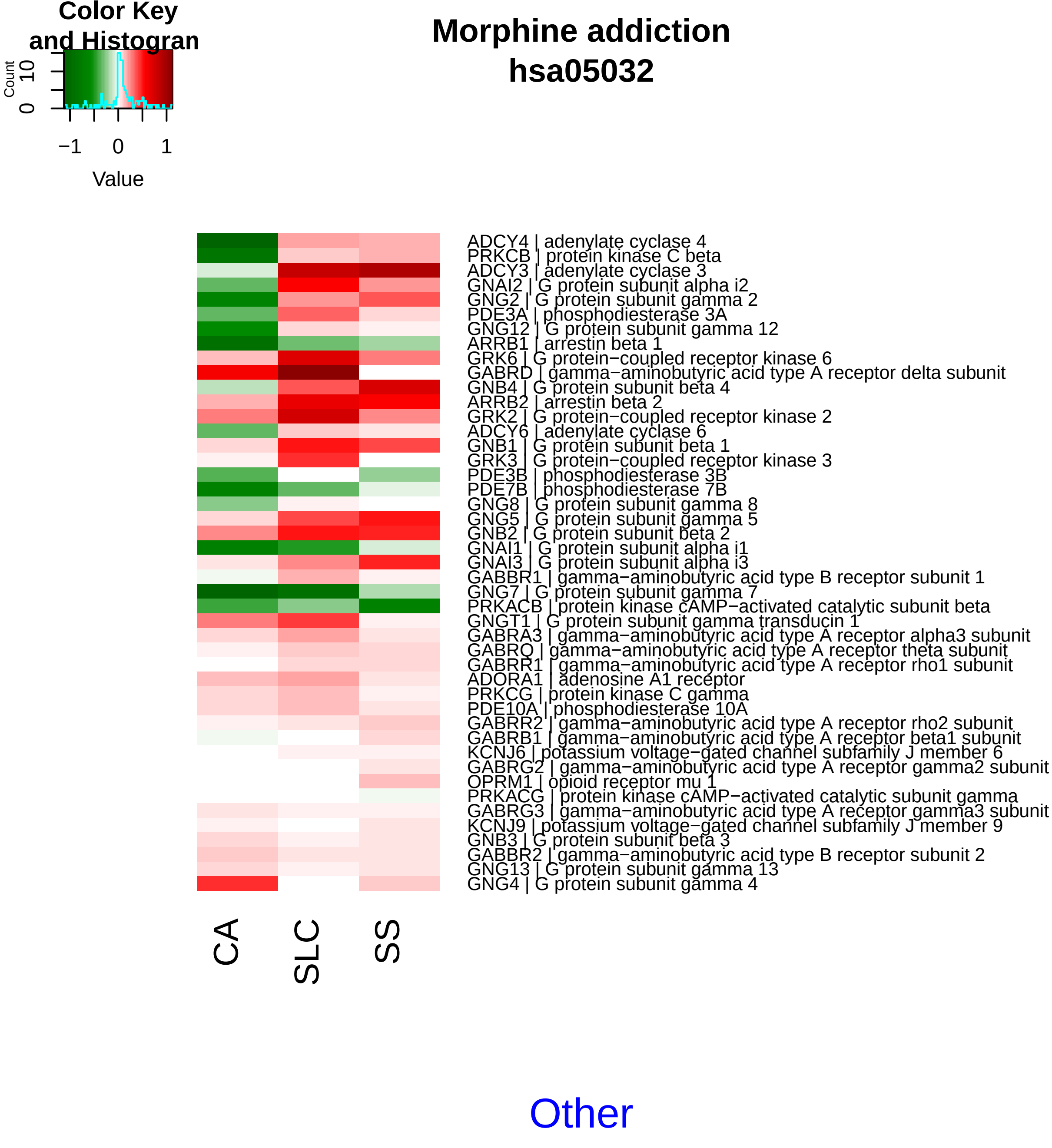
Morphine addiction
hsa05032
Color Key
and Histogran
Count
0
10
−1
0
1
Value
ADCY4 | adenylate cyclase 4
PRKCB | protein kinase C beta
ADCY3 | adenylate cyclase 3
GNAI2 | G protein subunit alpha i2
GNG2 | G protein subunit gamma 2
PDE3A | phosphodiesterase 3A
GNG12 | G protein subunit gamma 12
ARRB1 | arrestin beta 1
GRK6 | G protein−coupled receptor kinase 6
GABRD | gamma−aminobutyric acid type A receptor delta subunit
GNB4 | G protein subunit beta 4
ARRB2 | arrestin beta 2
GRK2 | G protein−coupled receptor kinase 2
ADCY6 | adenylate cyclase 6
GNB1 | G protein subunit beta 1
GRK3 | G protein−coupled receptor kinase 3
PDE3B | phosphodiesterase 3B
PDE7B | phosphodiesterase 7B
GNG8 | G protein subunit gamma 8
GNG5 | G protein subunit gamma 5
GNB2 | G protein subunit beta 2
GNAI1 | G protein subunit alpha i1
GNAI3 | G protein subunit alpha i3
GABBR1 | gamma−aminobutyric acid type B receptor subunit 1
GNG7 | G protein subunit gamma 7
PRKACB | protein kinase cAMP−activated catalytic subunit beta
GNGT1 | G protein subunit gamma transducin 1
GABRA3 | gamma−aminobutyric acid type A receptor alpha3 subunit
GABRQ | gamma−aminobutyric acid type A receptor theta subunit
GABRR1 | gamma−aminobutyric acid type A receptor rho1 subunit
ADORA1 | adenosine A1 receptor
PRKCG | protein kinase C gamma
PDE10A | phosphodiesterase 10A
GABRR2 | gamma−aminobutyric acid type A receptor rho2 subunit
GABRB1 | gamma−aminobutyric acid type A receptor beta1 subunit
KCNJ6 | potassium voltage−gated channel subfamily J member 6
GABRG2 | gamma−aminobutyric acid type A receptor gamma2 subunit
OPRM1 | opioid receptor mu 1
PRKACG | protein kinase cAMP−activated catalytic subunit gamma
GABRG3 | gamma−aminobutyric acid type A receptor gamma3 subunit
KCNJ9 | potassium voltage−gated channel subfamily J member 9
GNB3 | G protein subunit beta 3
GABBR2 | gamma−aminobutyric acid type B receptor subunit 2
GNG13 | G protein subunit gamma 13
GNG4 | G protein subunit gamma 4
CA
SLC
SS
Other

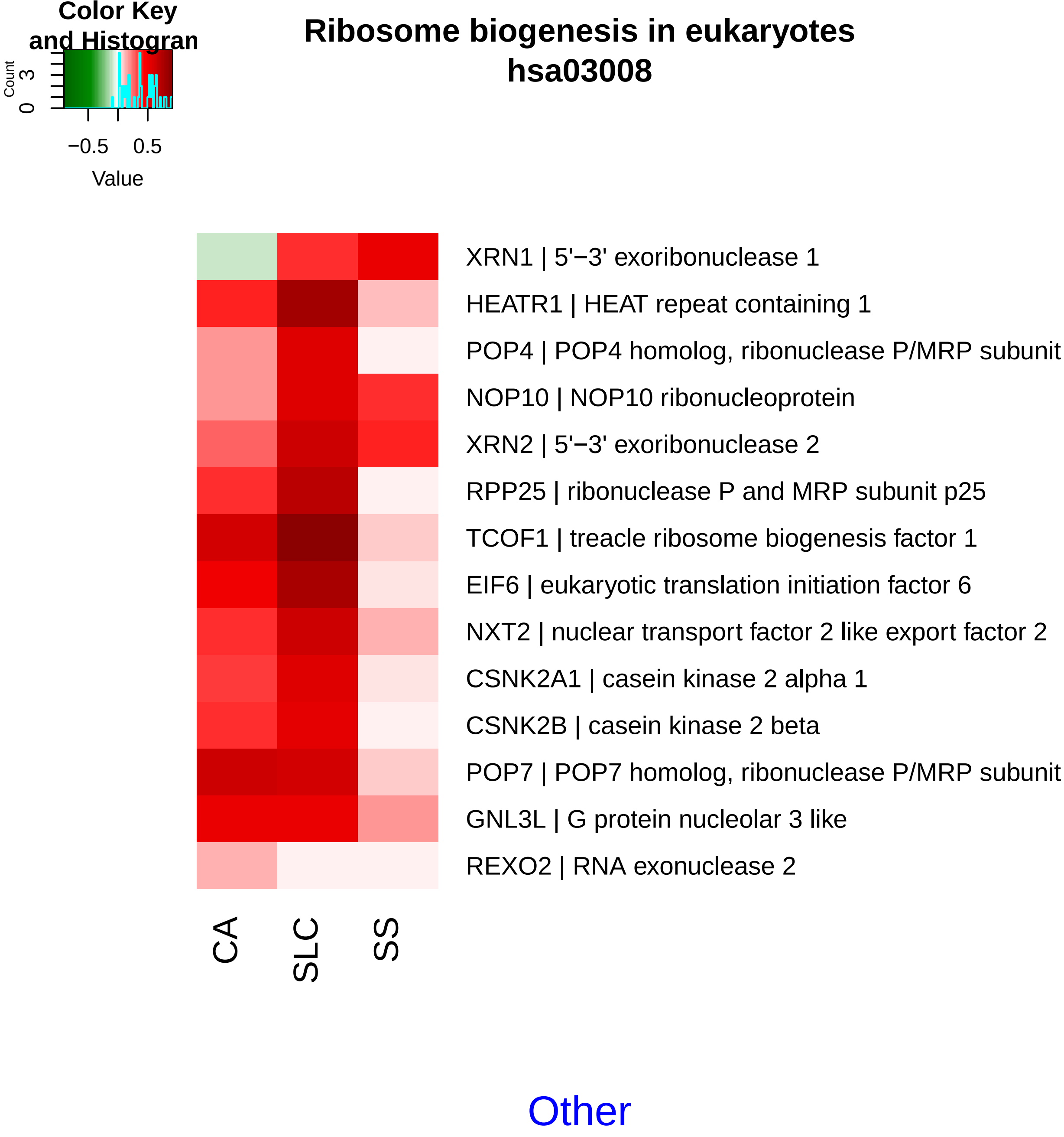
Color Key
and Histogram
Count
0
3
−0.5
0.5
Value
Ribosome biogenesis in eukaryotes
hsa03008
XRN1 | 5'−3' exoribonuclease 1
HEATR1 | HEAT repeat containing 1
POP4 | POP4 homolog, ribonuclease P/MRP subunit
NOP10 | NOP10 ribonucleoprotein
XRN2 | 5'−3' exoribonuclease 2
RPP25 | ribonuclease P and MRP subunit p25
TCOF1 | treacle ribosome biogenesis factor 1
EIF6 | eukaryotic translation initiation factor 6
NXT2 | nuclear transport factor 2 like export factor 2
CSNK2A1 | casein kinase 2 alpha 1
CSNK2B | casein kinase 2 beta
POP7 | POP7 homolog, ribonuclease P/MRP subunit
GNL3L | G protein nucleolar 3 like
REXO2 | RNA exonuclease 2
CA
SLC
SS
Other

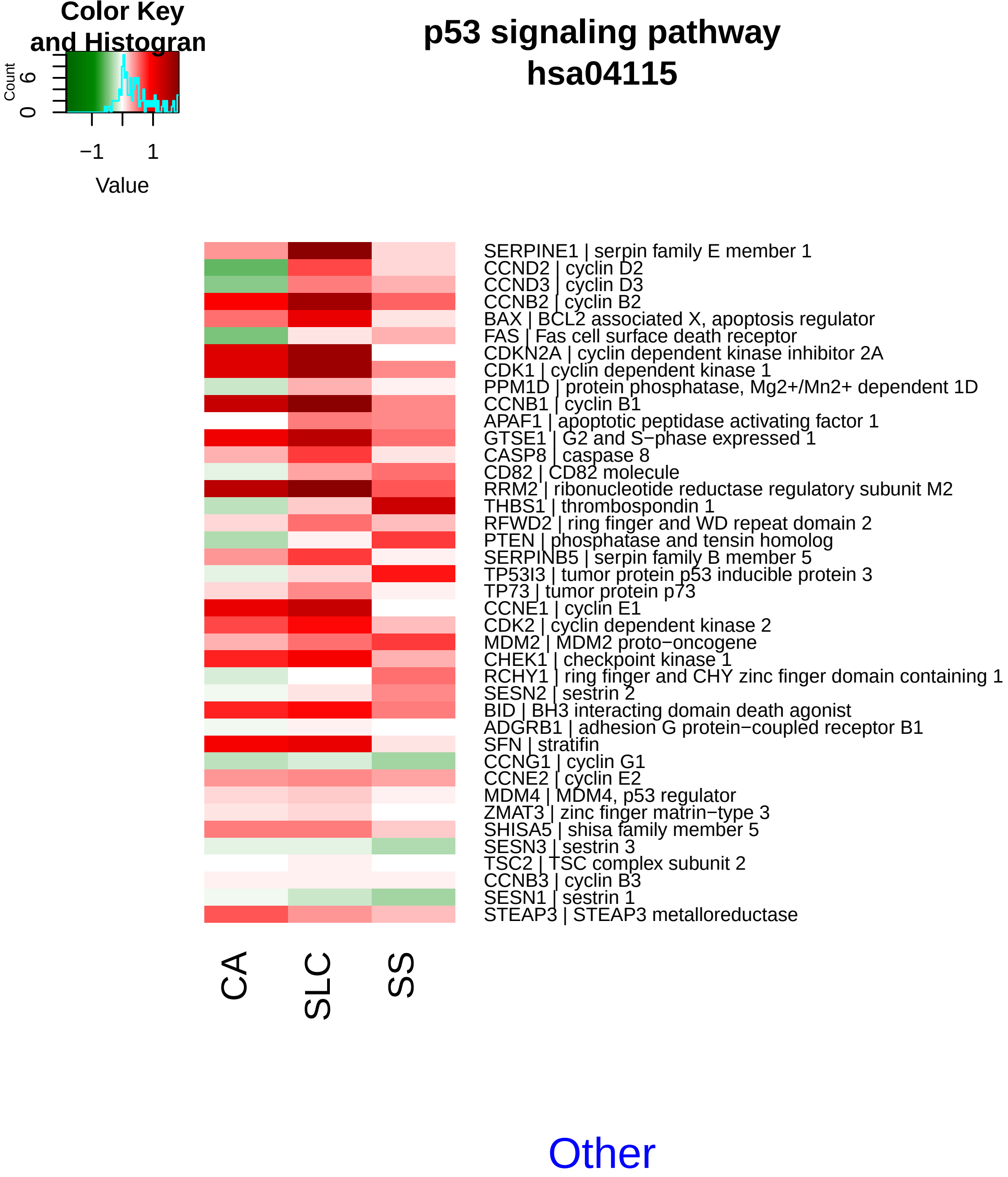

Color Key
and Histogram
Count
0
6
−1
1
Value
p53 signaling pathway
hsa04115
SERPINE1 | serpin family E member 1
CCND2 | cyclin D2
CCND3 | cyclin D3
CCNB2 | cyclin B2
BAX | BCL2 associated X, apoptosis regulator
FAS | Fas cell surface death receptor
CDKN2A | cyclin dependent kinase inhibitor 2A
CDK1 | cyclin dependent kinase 1
PPM1D | protein phosphatase, Mg2+/Mn2+ dependent 1D
CCNB1 | cyclin B1
APAF1 | apoptotic peptidase activating factor 1
GTSE1 | G2 and S−phase expressed 1
CASP8 | caspase 8
CD82 | CD82 molecule
RRM2 | ribonucleotide reductase regulatory subunit M2
THBS1 | thrombospondin 1
RFWD2 | ring finger and WD repeat domain 2
PTEN | phosphatase and tensin homolog
SERPINB5 | serpin family B member 5
TP53I3 | tumor protein p53 inducible protein 3
TP73 | tumor protein p73
CCNE1 | cyclin E1
CDK2 | cyclin dependent kinase 2
MDM2 | MDM2 proto−oncogene
CHEK1 | checkpoint kinase 1
RCHY1 | ring finger and CHY zinc finger domain containing 1
SESN2 | sestrin 2
BID | BH3 interacting domain death agonist
ADGRB1 | adhesion G protein−coupled receptor B1
SFN | stratifin
CCNG1 | cyclin G1
CCNE2 | cyclin E2
MDM4 | MDM4, p53 regulator
ZMAT3 | zinc finger matrin−type 3
SHISA5 | shisa family member 5
SESN3 | sestrin 3
TSC2 | TSC complex subunit 2
CCNB3 | cyclin B3
SESN1 | sestrin 1
STEAP3 | STEAP3 metalloreductase
CA
SLC
SS
Other

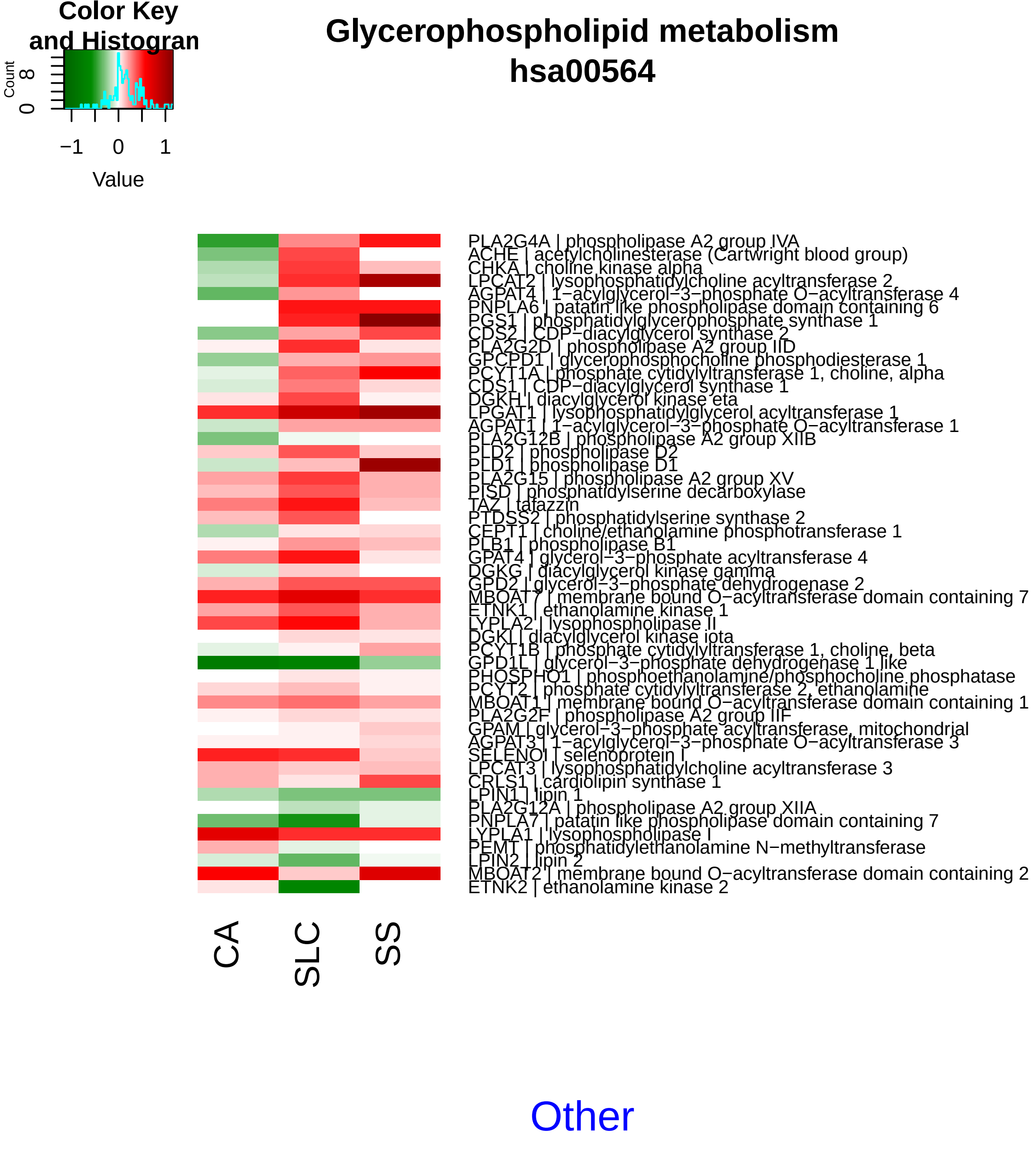
Color Key
and Histogran
Count
0
8
−1
0
1
Value
Glycerophospholipid metabolism
hsa00564
PLA2G4A | phospholipase A2 group IVA
ACHE | acetylcholinesterase (Cartwright blood group)
CHKA | choline kinase alpha
LPCAT2 | lysophosphatidylcholine acyltransferase 2
AGPAT4 | 1−acylglycerol−3−phosphate O−acyltransferase 4
PNPLA6 | patatin like phospholipase domain containing 6
PGS1 | phosphatidylglycerophosphate synthase 1
CDS2 | CDP−diacylglycerol synthase 2
PLA2G2D | phospholipase A2 group IID
GPCPD1 | glycerophosphocholine phosphodiesterase 1
PCYT1A | phosphate cytidylyltransferase 1, choline, alpha
CDS1 | CDP−diacylglycerol synthase 1
DGKH | diacylglycerol kinase eta
LPGAT1 | lysophosphatidylglycerol acyltransferase 1
AGPAT1 | 1−acylglycerol−3−phosphate O−acyltransferase 1
PLA2G12B | phospholipase A2 group XIIB
PLD2 | phospholipase D2
PLD1 | phospholipase D1
PLA2G15 | phospholipase A2 group XV
PISD | phosphatidylserine decarboxylase
TAZ | tafazzin
PTDSS2 | phosphatidylserine synthase 2
CEPT1 | choline/ethanolamine phosphotransferase 1
PLB1 | phospholipase B1
GPAT4 | glycerol−3−phosphate acyltransferase 4
DGKG | diacylglycerol kinase gamma
GPD2 | glycerol−3−phosphate dehydrogenase 2
MBOAT7 | membrane bound O−acyltransferase domain containing 7
ETNK1 | ethanolamine kinase 1
LYPLA2 | lysophospholipase II
DGKI | diacylglycerol kinase iota
PCYT1B | phosphate cytidylyltransferase 1, choline, beta
GPD1L | glycerol−3−phosphate dehydrogenase 1 like
PHOSPHO1 | phosphoethanolamine/phosphocholine phosphatase
PCYT2 | phosphate cytidylyltransferase 2, ethanolamine
MBOAT1 | membrane bound O−acyltransferase domain containing 1
PLA2G2F | phospholipase A2 group IIF
GPAM | glycerol−3−phosphate acyltransferase, mitochondrial
AGPAT3 | 1−acylglycerol−3−phosphate O−acyltransferase 3
SELENOI | selenoprotein I
LPCAT3 | lysophosphatidylcholine acyltransferase 3
CRLS1 | cardiolipin synthase 1
LPIN1 | lipin 1
PLA2G12A | phospholipase A2 group XIIA
PNPLA7 | patatin like phospholipase domain containing 7
LYPLA1 | lysophospholipase I
PEMT | phosphatidylethanolamine N−methyltransferase
LPIN2 | lipin 2
MBOAT2 | membrane bound O−acyltransferase domain containing 2
ETNK2 | ethanolamine kinase 2
CA
SLC
SS
Other

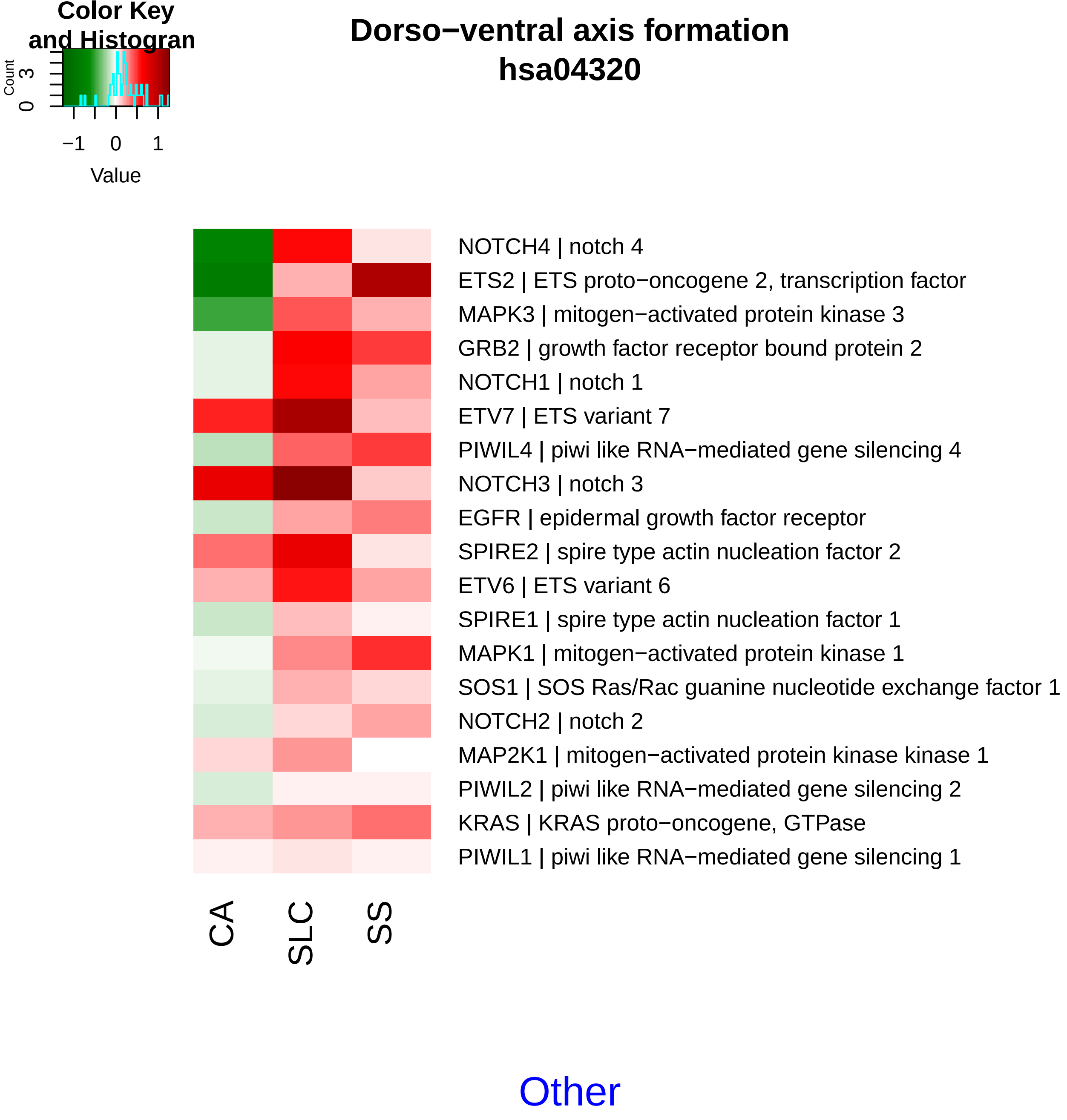

Color Key
and Histogran
Count
0
3
−1
0
1
Value
Dorso−ventral axis formation
hsa04320
NOTCH4 | notch 4
ETS2 | ETS proto−oncogene 2, transcription factor
MAPK3 | mitogen−activated protein kinase 3
GRB2 | growth factor receptor bound protein 2
NOTCH1 | notch 1
ETV7 | ETS variant 7
PIWIL4 | piwi like RNA−mediated gene silencing 4
NOTCH3 | notch 3
EGFR | epidermal growth factor receptor
SPIRE2 | spire type actin nucleation factor 2
ETV6 | ETS variant 6
SPIRE1 | spire type actin nucleation factor 1
MAPK1 | mitogen−activated protein kinase 1
SOS1 | SOS Ras/Rac guanine nucleotide exchange factor 1
NOTCH2 | notch 2
MAP2K1 | mitogen−activated protein kinase kinase 1
PIWIL2 | piwi like RNA−mediated gene silencing 2
KRAS | KRAS proto−oncogene, GTPase
PIWIL1 | piwi like RNA−mediated gene silencing 1
CA
SLC
SS
Other

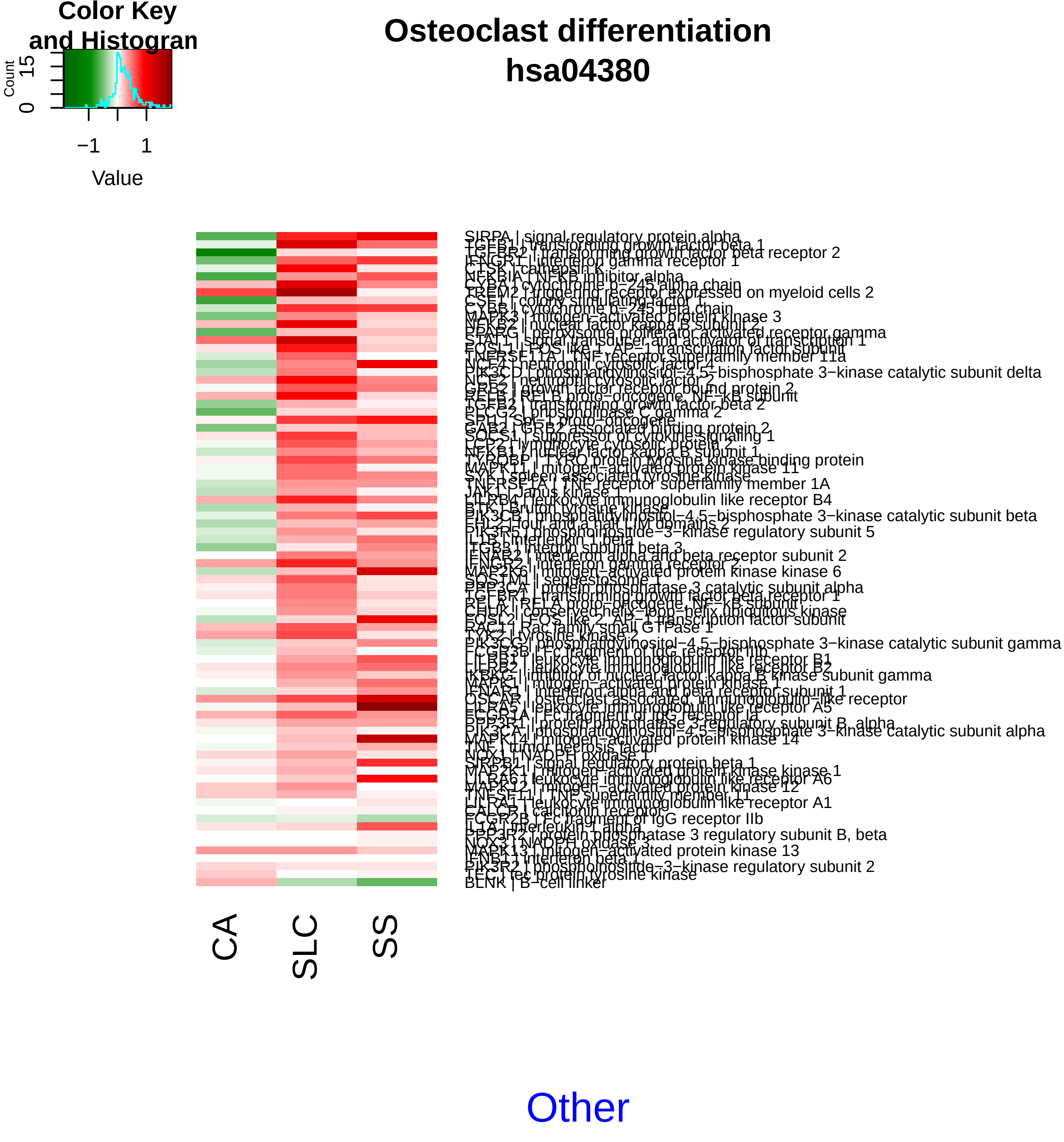
Color Key
and Histogram
Count
0
15
−1
1
Value
Osteoclast differentiation
hsa04380
SIRPA | signal regulatory protein alpha
TGFB1 | transforming growth factor beta 1
TGFBR2 | transforming growth factor beta receptor 2
IFNGR1 | interferon gamma receptor 1
CTSK | cathepsin K
NFKBIA | NFKB inhibitor alpha
CYBA | cytochrome b−245 alpha chain
TREM2 | triggering receptor expressed on myeloid cells 2
CSF1 | colony stimulating factor 1
CYBB | cytochrome b−245 beta chain
MAPK3 | mitogen−activated protein kinase 3
NFKB2 | nuclear factor kappa B subunit 2
PPARG | peroxisome proliferator activated receptor gamma
STAT1 | signal transducer and activator of transcription 1
FOSL1 | FOS like 1, AP−1 transcription factor subunit
TNFRSF11A | TNF receptor superfamily member 11a
NCF4 | neutrophil cytosolic factor 4
PIK3CD | phosphatidylinositol−4,5−bisphosphate 3−kinase catalytic subunit delta
NCF2 | neutrophil cytosolic factor 2
GRB2 | growth factor receptor bound protein 2
RELB | RELB proto−oncogene, NF−kB subunit
TGFB2 | transforming growth factor beta 2
PLCG2 | phospholipase C gamma 2
SPI1 | Spi−1 proto−oncogene
GAB2 | GRB2 associated binding protein 2
SOCS1 | suppressor of cytokine signaling 1
LCP2 | lymphocyte cytosolic protein 2
NFKB1 | nuclear factor kappa B subunit 1
TYROBP | TYRO protein tyrosine kinase binding protein
MAPK11 | mitogen−activated protein kinase 11
SYK | spleen associated tyrosine kinase
TNFRSF1A | TNF receptor superfamily member 1A
JAK1 | Janus kinase 1
LILRB4 | leukocyte immunoglobulin like receptor B4
BTK | Bruton tyrosine kinase
PIK3CB | phosphatidylinositol−4,5−bisphosphate 3−kinase catalytic subunit beta
FHL2 | four and a half LIM domains 2
PIK3R5 | phosphoinositide−3−kinase regulatory subunit 5
IL1B | interleukin 1 beta
ITGB3 | integrin subunit beta 3
IFNAR2 | interferon alpha and beta receptor subunit 2
IFNGR2 | interferon gamma receptor 2
MAP2K6 | mitogen−activated protein kinase kinase 6
SQSTM1 | sequestosome 1
PPP3CA | protein phosphatase 3 catalytic subunit alpha
TGFBR1 | transforming growth factor beta receptor 1
RELA | RELA proto−oncogene, NF−kB subunit
CHUK | conserved helix−loop−helix ubiquitous kinase
FOSL2 | FOS like 2, AP−1 transcription factor subunit
RAC1 | Rac family small GTPase 1
TYK2 | tyrosine kinase 2
PIK3CG | phosphatidylinositol−4,5−bisphosphate 3−kinase catalytic subunit gamma
FCGR3B | Fc fragment of IgG receptor IIIb
LILRB1 | leukocyte immunoglobulin like receptor B1
LILRB2 | leukocyte immunoglobulin like receptor B2
IKBKG | inhibitor of nuclear factor kappa B kinase subunit gamma
MAPK1 | mitogen−activated protein kinase 1
IFNAR1 | interferon alpha and beta receptor subunit 1
OSCAR | osteoclast associated, immunoglobulin−like receptor
LILRA5 | leukocyte immunoglobulin like receptor A5
FCGR2A | Fc fragment of IgG receptor IIa
PPP3R1 | protein phosphatase 3 regulatory subunit B, alpha
PIK3CA | phosphatidylinositol−4,5−bisphosphate 3−kinase catalytic subunit alpha
MAPK14 | mitogen−activated protein kinase 14
TNF | tumor necrosis factor
NOX1 | NADPH oxidase 1
SIRPB1 | signal regulatory protein beta 1
MAP2K1 | mitogen−activated protein kinase kinase 1
LILRA6 | leukocyte immunoglobulin like receptor A6
MAPK12 | mitogen−activated protein kinase 12
TNFSF11 | TNF superfamily member 11
LILRA1 | leukocyte immunoglobulin like receptor A1
CALCR | calcitonin receptor
FCGR2B | Fc fragment of IgG receptor IIb
IL1A | interleukin 1 alpha
PPP3R2 | protein phosphatase 3 regulatory subunit B, beta
NOX3 | NADPH oxidase 3
MAPK13 | mitogen−activated protein kinase 13
IFNB1 | interferon beta 1
PIK3R2 | phosphoinositide−3−kinase regulatory subunit 2
TEC | tec protein tyrosine kinase
BLNK | B−cell linker
CA
SLC
SS
Other

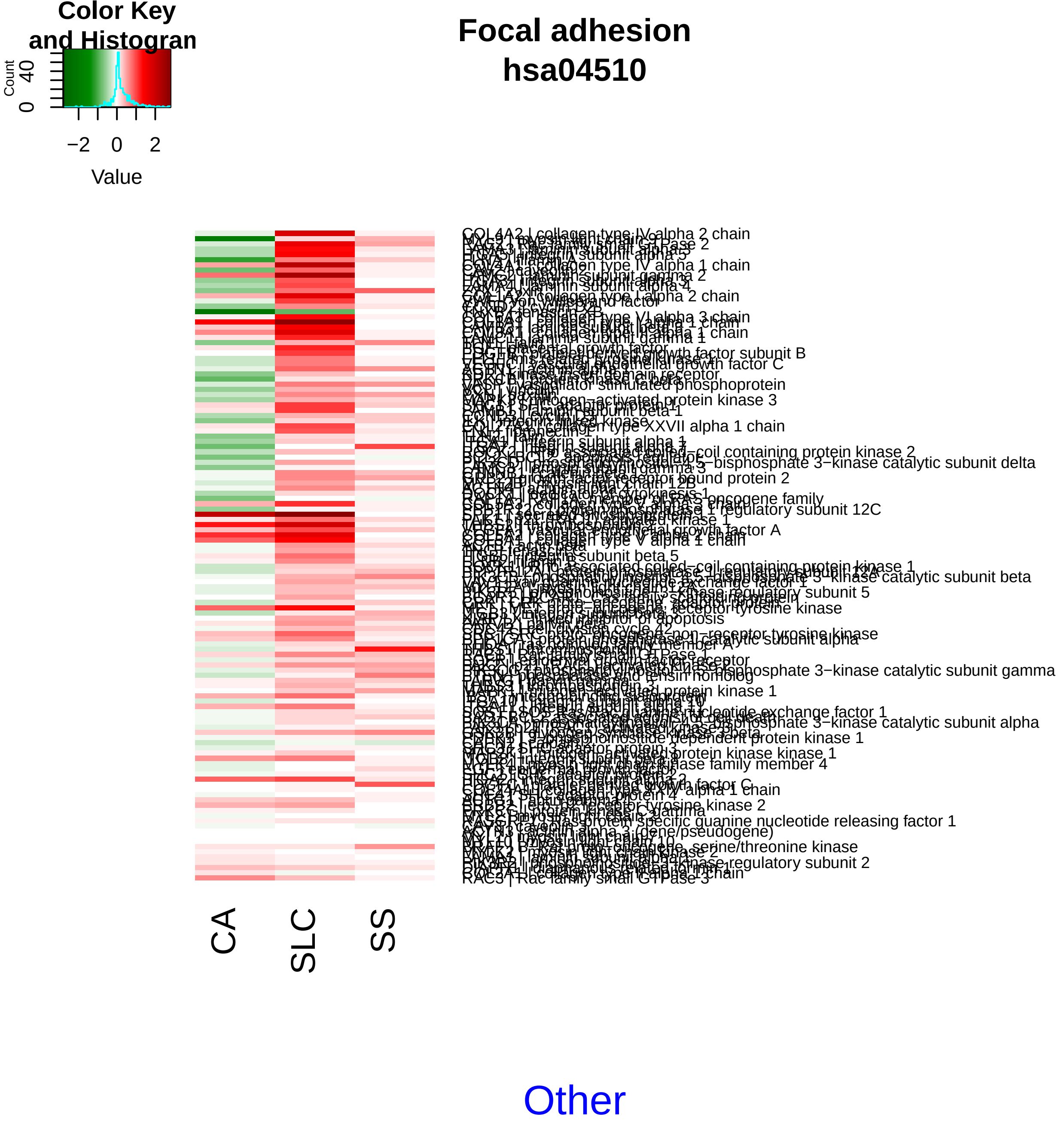
Color Key and Histogran
Count
0
40
−2
0
2
Value
Focal adhesion
hsa04510
COL4A2 | collagen type IV alpha 2 chain
RAC3 | Rac family small GTPase 3
CA
SLC
SS
Other

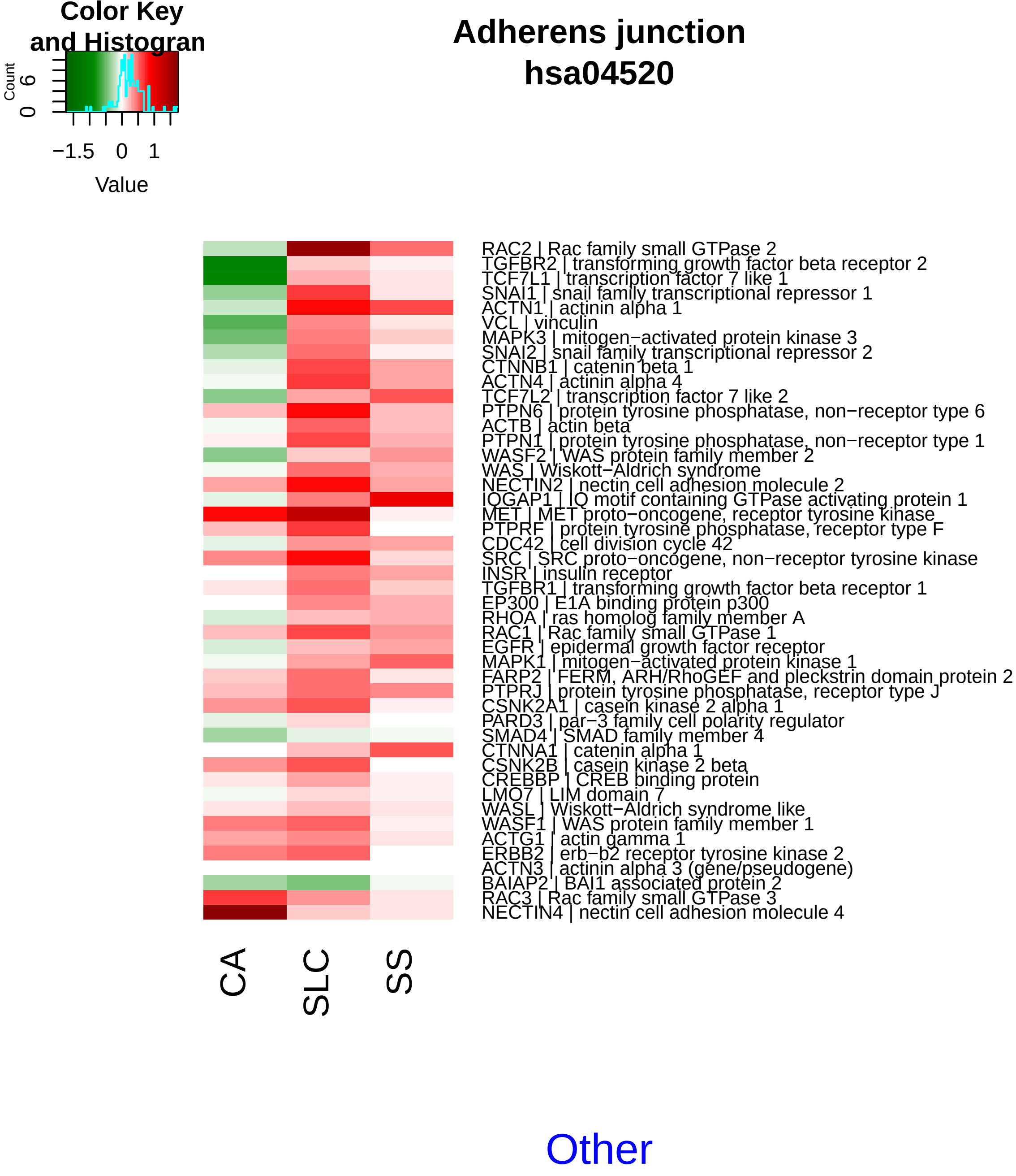
Color Key
and Histogran
Count
0
6
−1.5
0
1
Value
Adherens junction
hsa04520
RAC2 | Rac family small GTPase 2
TGFBR2 | transforming growth factor beta receptor 2
TCF7L1 | transcription factor 7 like 1
SNAI1 | snail family transcriptional repressor 1
ACTN1 | actinin alpha 1
VCL | vinculin
MAPK3 | mitogen−activated protein kinase 3
SNAI2 | snail family transcriptional repressor 2
CTNNB1 | catenin beta 1
ACTN4 | actinin alpha 4
TCF7L2 | transcription factor 7 like 2
PTPN6 | protein tyrosine phosphatase, non−receptor type 6
ACTB | actin beta
PTPN1 | protein tyrosine phosphatase, non−receptor type 1
WASF2 | WAS protein family member 2
WAS | Wiskott−Aldrich syndrome
NECTIN2 | nectin cell adhesion molecule 2
IQGAP1 | IQ motif containing GTPase activating protein 1
MET | MET proto−oncogene, receptor tyrosine kinase
PTPRF | protein tyrosine phosphatase, receptor type F
CDC42 | cell division cycle 42
SRC | SRC proto−oncogene, non−receptor tyrosine kinase
INSR | insulin receptor
TGFBR1 | transforming growth factor beta receptor 1
EP300 | E1A binding protein p300
RHOA | ras homolog family member A
RAC1 | Rac family small GTPase 1
EGFR | epidermal growth factor receptor
MAPK1 | mitogen−activated protein kinase 1
FARP2 | FERM, ARH/RhoGEF and pleckstrin domain protein 2
PTPRJ | protein tyrosine phosphatase, receptor type J
CSNK2A1 | casein kinase 2 alpha 1
PARD3 | par−3 family cell polarity regulator
SMAD4 | SMAD family member 4
CTNNA1 | catenin alpha 1
CSNK2B | casein kinase 2 beta
CREBBP | CREB binding protein
LMO7 | LIM domain 7
WASL | Wiskott−Aldrich syndrome like
WASF1 | WAS protein family member 1
ACTG1 | actin gamma 1
ERBB2 | erb−b2 receptor tyrosine kinase 2
ACTN3 | actinin alpha 3 (gene/pseudogene)
BAIAP2 | BAI1 associated protein 2
RAC3 | Rac family small GTPase 3
NECTIN4 | nectin cell adhesion molecule 4
CA
SLC
SS
Other

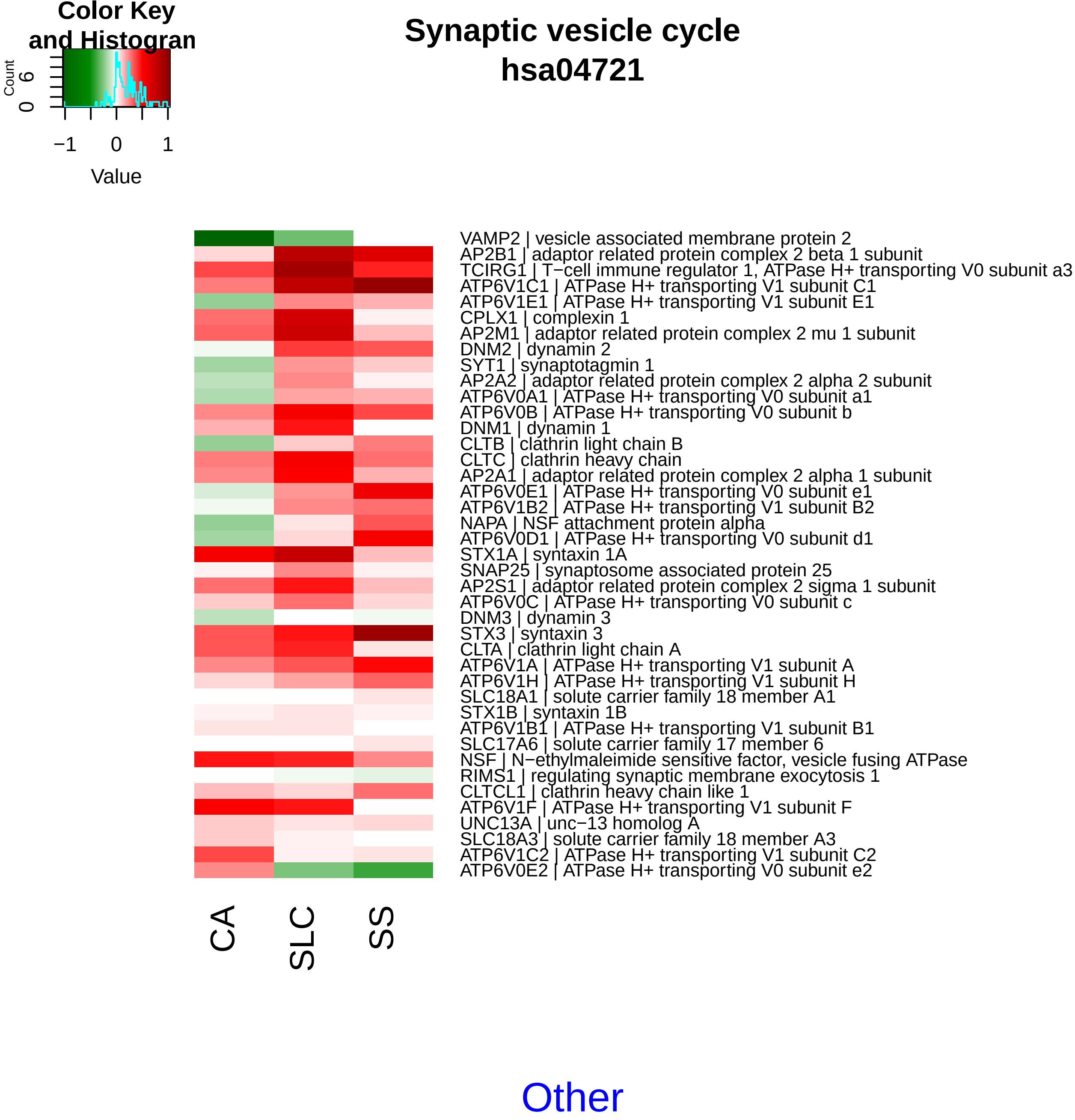
Color Key
and Histogram
Count
0
6
−1
0
1
Value
Synaptic vesicle cycle
hsa04721
VAMP2 | vesicle associated membrane protein 2
AP2B1 | adaptor related protein complex 2 beta 1 subunit
TCIRG1 | T−cell immune regulator 1, ATPase H+ transporting V0 subunit a3
ATP6V1C1 | ATPase H+ transporting V1 subunit C1
ATP6V1E1 | ATPase H+ transporting V1 subunit E1
CPLX1 | complexin 1
AP2M1 | adaptor related protein complex 2 mu 1 subunit
DNM2 | dynamin 2
SYT1 | synaptotagmin 1
AP2A2 | adaptor related protein complex 2 alpha 2 subunit
ATP6V0A1 | ATPase H+ transporting V0 subunit a1
ATP6V0B | ATPase H+ transporting V0 subunit b
DNM1 | dynamin 1
CLTB | clathrin light chain B
CLTC | clathrin heavy chain
AP2A1 | adaptor related protein complex 2 alpha 1 subunit
ATP6V0E1 | ATPase H+ transporting V0 subunit e1
ATP6V1B2 | ATPase H+ transporting V1 subunit B2
NAPA | NSF attachment protein alpha
ATP6V0D1 | ATPase H+ transporting V0 subunit d1
STX1A | syntaxin 1A
SNAP25 | synaptosome associated protein 25
AP2S1 | adaptor related protein complex 2 sigma 1 subunit
ATP6V0C | ATPase H+ transporting V0 subunit c
DNM3 | dynamin 3
STX3 | syntaxin 3
CLTA | clathrin light chain A
ATP6V1A | ATPase H+ transporting V1 subunit A
ATP6V1H | ATPase H+ transporting V1 subunit H
SLC18A1 | solute carrier family 18 member A1
STX1B | syntaxin 1B
ATP6V1B1 | ATPase H+ transporting V1 subunit B1
SLC17A6 | solute carrier family 17 member 6
NSF | N−ethylmaleimide sensitive factor, vesicle fusing ATPase
RIMS1 | regulating synaptic membrane exocytosis 1
CLTCL1 | clathrin heavy chain like 1
ATP6V1F | ATPase H+ transporting V1 subunit F
UNC13A | unc−13 homolog A
SLC18A3 | solute carrier family 18 member A3
ATP6V1C2 | ATPase H+ transporting V1 subunit C2
ATP6V0E2 | ATPase H+ transporting V0 subunit e2
CA
SLC
SS
Other

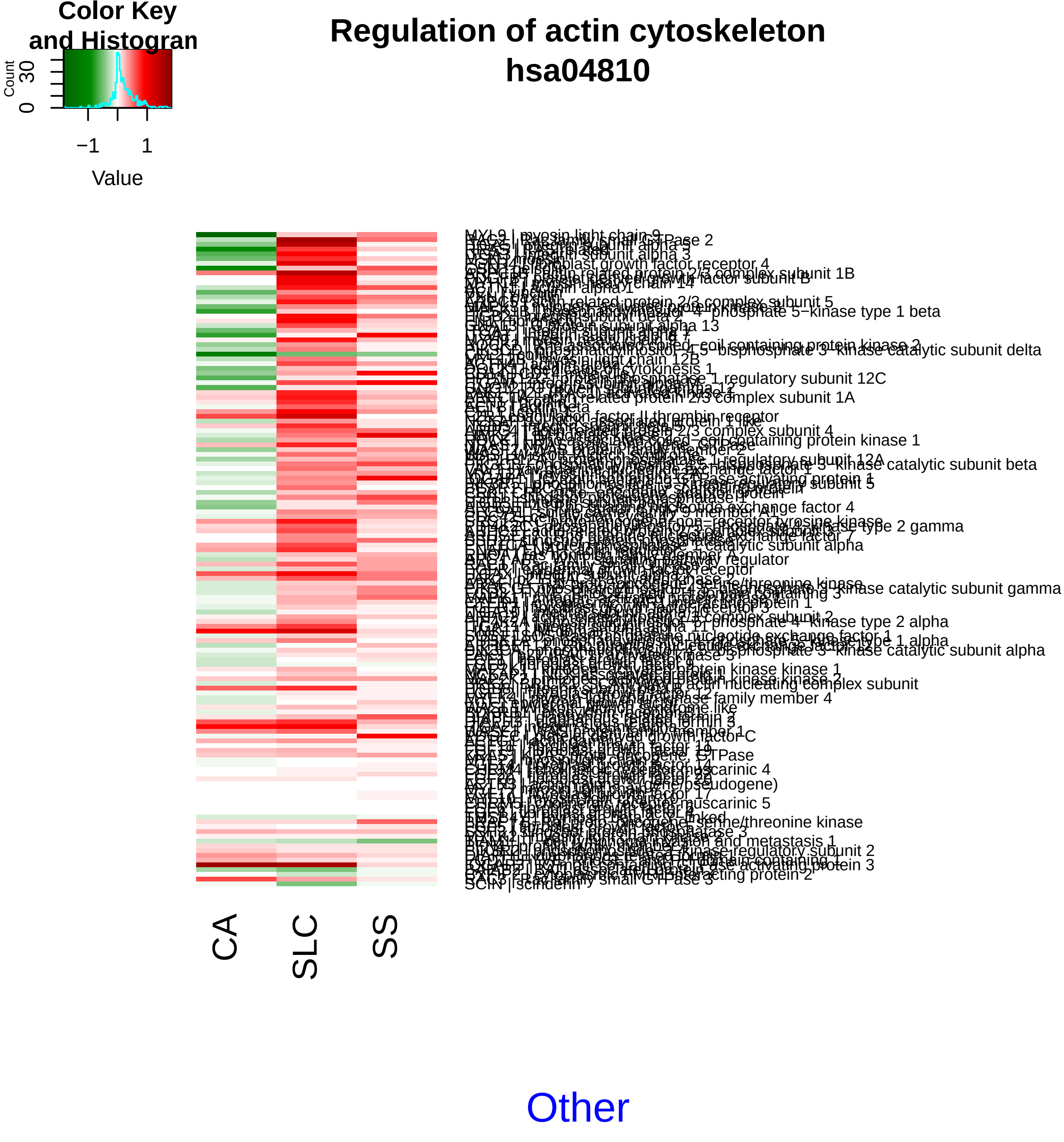
Color Key
and Histogran
Count
0
30
−1
1
Value
Regulation of actin cytoskeleton
hsa04810
MYL9 | myosin light chain 9
SCIN | scinderin
CA
SLC
SS
Other

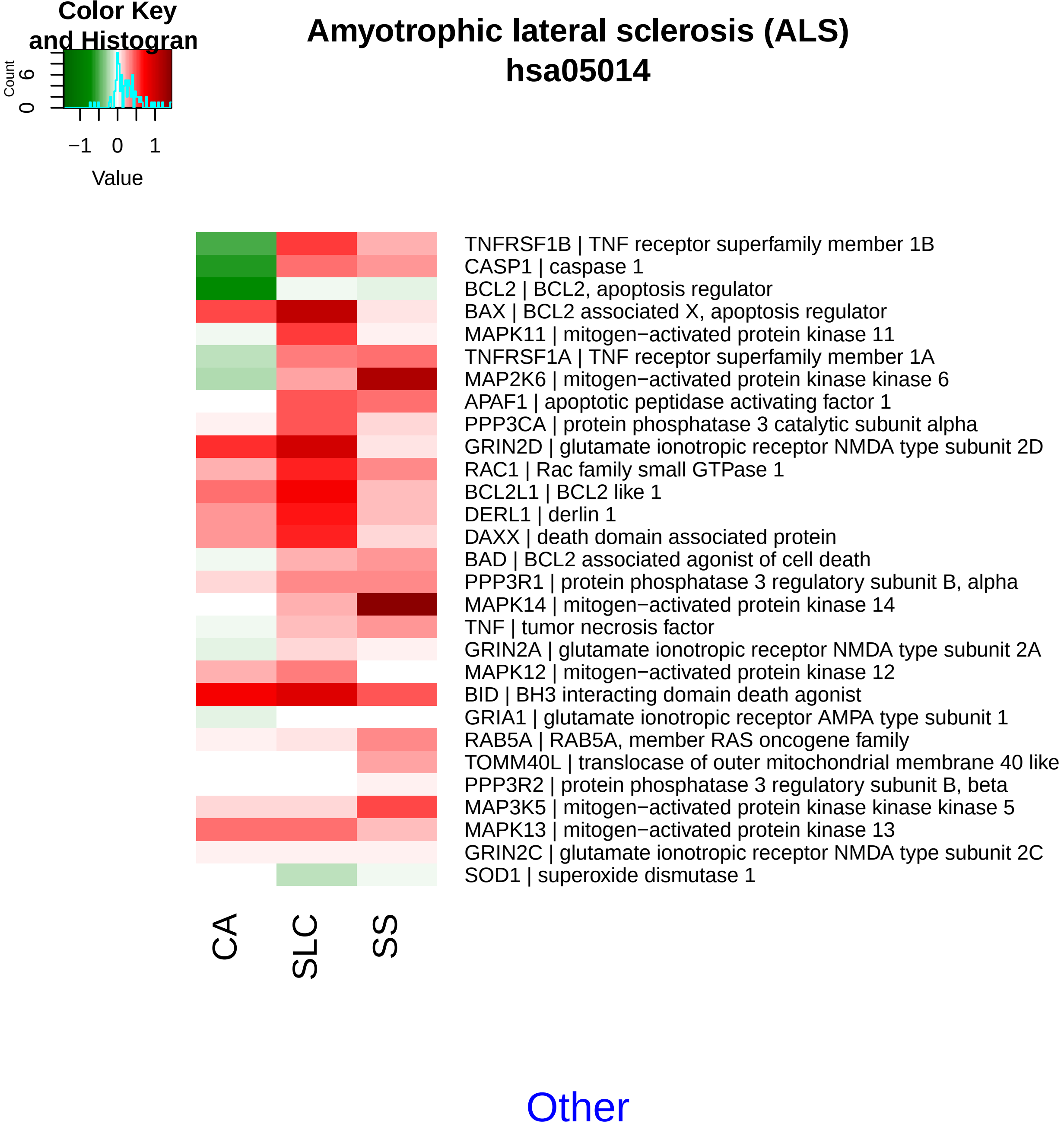

Color Key
and Histogram
Count
0
6
−1
0
1
Value
Amyotrophic lateral sclerosis (ALS)
hsa05014
TNFRSF1B | TNF receptor superfamily member 1B
CASP1 | caspase 1
BCL2 | BCL2, apoptosis regulator
BAX | BCL2 associated X, apoptosis regulator
MAPK11 | mitogen−activated protein kinase 11
TNFRSF1A | TNF receptor superfamily member 1A
MAP2K6 | mitogen−activated protein kinase kinase 6
APAF1 | apoptotic peptidase activating factor 1
PPP3CA | protein phosphatase 3 catalytic subunit alpha
GRIN2D | glutamate ionotropic receptor NMDA type subunit 2D
RAC1 | Rac family small GTPase 1
BCL2L1 | BCL2 like 1
DERL1 | derlin 1
DAXX | death domain associated protein
BAD | BCL2 associated agonist of cell death
PPP3R1 | protein phosphatase 3 regulatory subunit B, alpha
MAPK14 | mitogen−activated protein kinase 14
TNF | tumor necrosis factor
GRIN2A | glutamate ionotropic receptor NMDA type subunit 2A
MAPK12 | mitogen−activated protein kinase 12
BID | BH3 interacting domain death agonist
GRIA1 | glutamate ionotropic receptor AMPA type subunit 1
RAB5A | RAB5A, member RAS oncogene family
TOMM40L | translocase of outer mitochondrial membrane 40 like
PPP3R2 | protein phosphatase 3 regulatory subunit B, beta
MAP3K5 | mitogen−activated protein kinase kinase kinase 5
MAPK13 | mitogen−activated protein kinase 13
GRIN2C | glutamate ionotropic receptor NMDA type subunit 2C
SOD1 | superoxide dismutase 1
CA
SLC
SS
Other

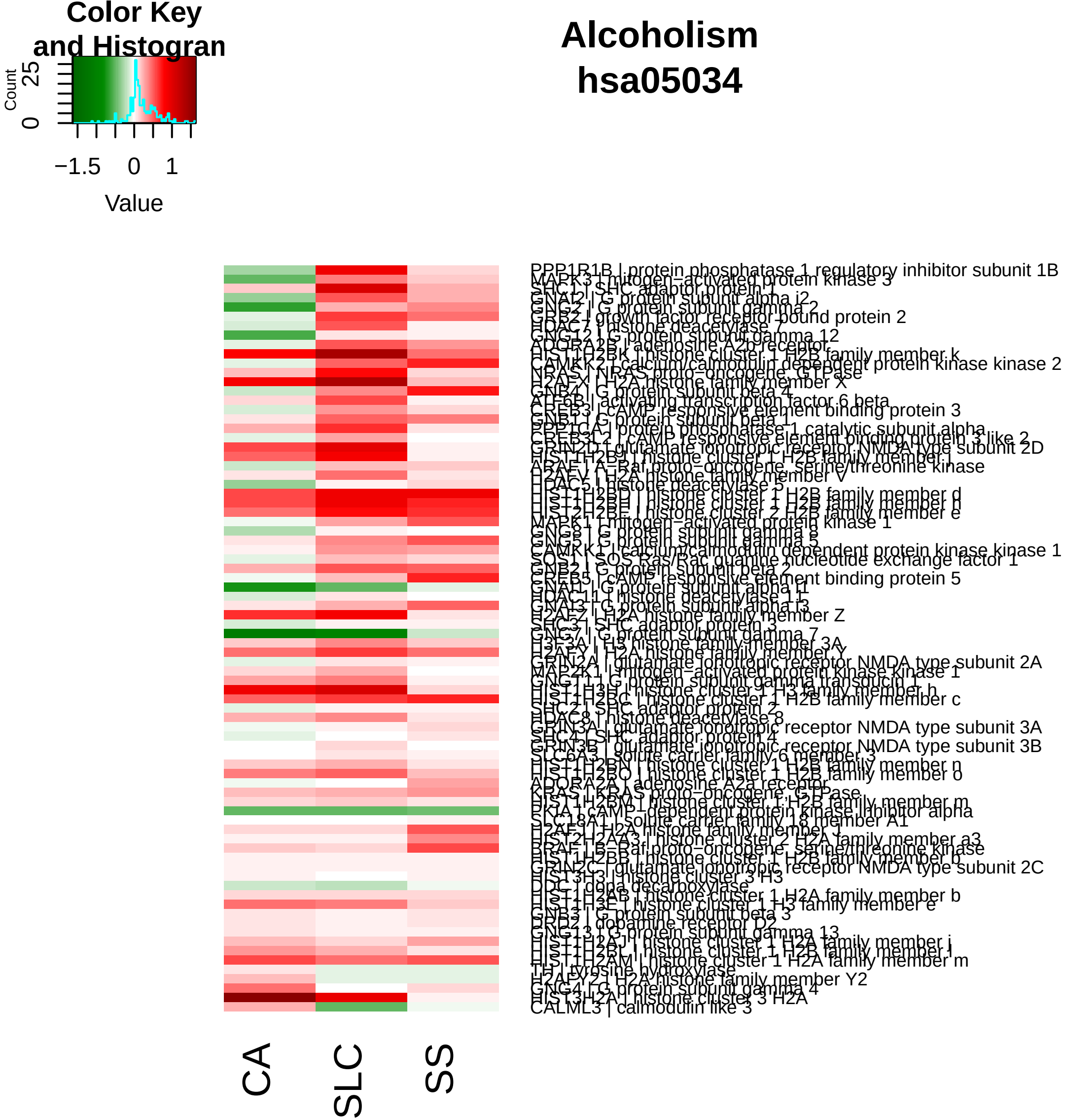
Color Key
and Histogran
Count
0
25
−1.5
0
1
Value
Alcoholism
hsa05034
PPP1R1B | protein phosphatase 1 regulatory inhibitor subunit 1B
MAPK3 | mitogen−activated protein kinase 3
SHC1 | SHC adaptor protein 1
GNAI2 | G protein subunit alpha i2
GNG2 | G protein subunit gamma 2
GRB2 | growth factor receptor bound protein 2
HDAC7 | histone deacetylase 7
GNG12 | G protein subunit gamma 12
ADORA2B | adenosine A2b receptor
HIST1H2BK | histone cluster 1 H2B family member k
CAMKK2 | calcium/calmodulin dependent protein kinase kinase 2
NRAS | NRAS proto−oncogene, GTPase
H2AFX | H2A histone family member X
GNB4 | G protein subunit beta 4
ATF6B | activating transcription factor 6 beta
CREB3 | cAMP responsive element binding protein 3
GNB1 | G protein subunit beta 1
PPP1CA | protein phosphatase 1 catalytic subunit alpha
CREB3L2 | cAMP responsive element binding protein 3 like 2
GRIN2D | glutamate ionotropic receptor NMDA type subunit 2D
HIST1H2BJ | histone cluster 1 H2B family member j
ARAF | A−Raf proto−oncogene, serine/threonine kinase
H2AFV | H2A histone family member V
HDAC5 | histone deacetylase 5
HIST1H2BD | histone cluster 1 H2B family member d
HIST1H2BH | histone cluster 1 H2B family member h
HIST2H2BE | histone cluster 2 H2B family member e
MAPK1 | mitogen−activated protein kinase 1
GNG8 | G protein subunit gamma 8
GNG5 | G protein subunit gamma 5
CAMKK1 | calcium/calmodulin dependent protein kinase kinase 1
SOS1 | SOS Ras/Rac guanine nucleotide exchange factor 1
GNB2 | G protein subunit beta 2
CREB5 | cAMP responsive element binding protein 5
GNAI1 | G protein subunit alpha i1
HDAC11 | histone deacetylase 11
GNAI3 | G protein subunit alpha i3
H2AFZ | H2A histone family member Z
SHC3 | SHC adaptor protein 3
GNG7 | G protein subunit gamma 7
H3F3A | H3 histone family member 3A
H2AFY | H2A histone family member Y
GRIN2A | glutamate ionotropic receptor NMDA type subunit 2A
MAP2K1 | mitogen−activated protein kinase kinase 1
GNGT1 | G protein subunit gamma transducin 1
HIST1H3H | histone cluster 1 H3 family member h
HIST1H2BC | histone cluster 1 H2B family member c
SHC2 | SHC adaptor protein 2
HDAC8 | histone deacetylase 8
GRIN3A | glutamate ionotropic receptor NMDA type subunit 3A
SHC4 | SHC adaptor protein 4
GRIN3B | glutamate ionotropic receptor NMDA type subunit 3B
SLC6A3 | solute carrier family 6 member 3
HIST1H2BN | histone cluster 1 H2B family member n
HIST1H2BO | histone cluster 1 H2B family member o
ADORA2A | adenosine A2a receptor
KRAS | KRAS proto−oncogene, GTPase
HIST1H2BM | histone cluster 1 H2B family member m
PKIA | cAMP−dependent protein kinase inhibitor alpha
SLC18A1 | solute carrier family 18 member A1
H2AFJ | H2A histone family member J
HIST2H2AA3 | histone cluster 2 H2A family member a3
BRAF | B−Raf proto−oncogene, serine/threonine kinase
HIST1H2BB | histone cluster 1 H2B family member b
GRIN2C | glutamate ionotropic receptor NMDA type subunit 2C
HIST3H3 | histone cluster 3 H3
DDC | dopa decarboxylase
HIST1H2AB | histone cluster 1 H2A family member b
HIST1H3E | histone cluster 1 H3 family member e
GNB3 | G protein subunit beta 3
DRD2 | dopamine receptor D2
GNG13 | G protein subunit gamma 13
HIST1H2AJ | histone cluster 1 H2A family member j
HIST1H2BL | histone cluster 1 H2B family member l
HIST1H2AM | histone cluster 1 H2A family member m
TH | tyrosine hydroxylase
H2AFY2 | H2A histone family member Y2
GNG4 | G protein subunit gamma 4
HIST3H2A | histone cluster 3 H2A
CALML3 | calmodulin like 3
CA
SLC
SS

Other

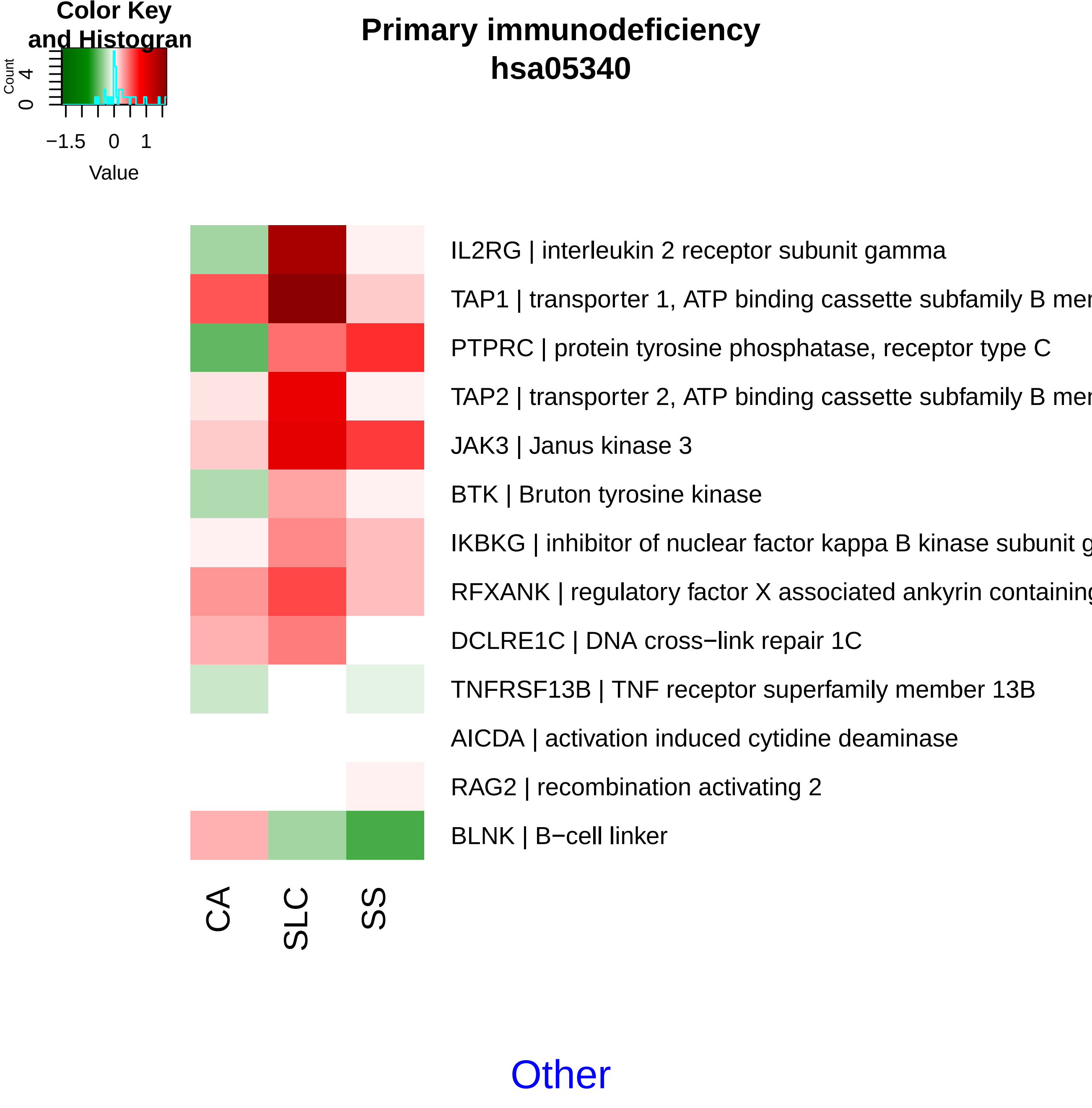
Color Key
and Histogram
Count
0
4
−1.5
0
1
Value
Primary immunodeficiency
hsa05340
IL2RG | interleukin 2 receptor subunit gamma
TAP1 | transporter 1, ATP binding cassette subfamily B mer
PTPRC | protein tyrosine phosphatase, receptor type C
TAP2 | transporter 2, ATP binding cassette subfamily B mer
JAK3 | Janus kinase 3
BTK | Bruton tyrosine kinase
IKBKG | inhibitor of nuclear factor kappa B kinase subunit g
RFXANK | regulatory factor X associated ankyrin containing
DCLRE1C | DNA cross−link repair 1C
TNFRSF13B | TNF receptor superfamily member 13B
AICDA | activation induced cytidine deaminase
RAG2 | recombination activating 2
BLNK | B−cell linker
CA
SLC
SS
Other

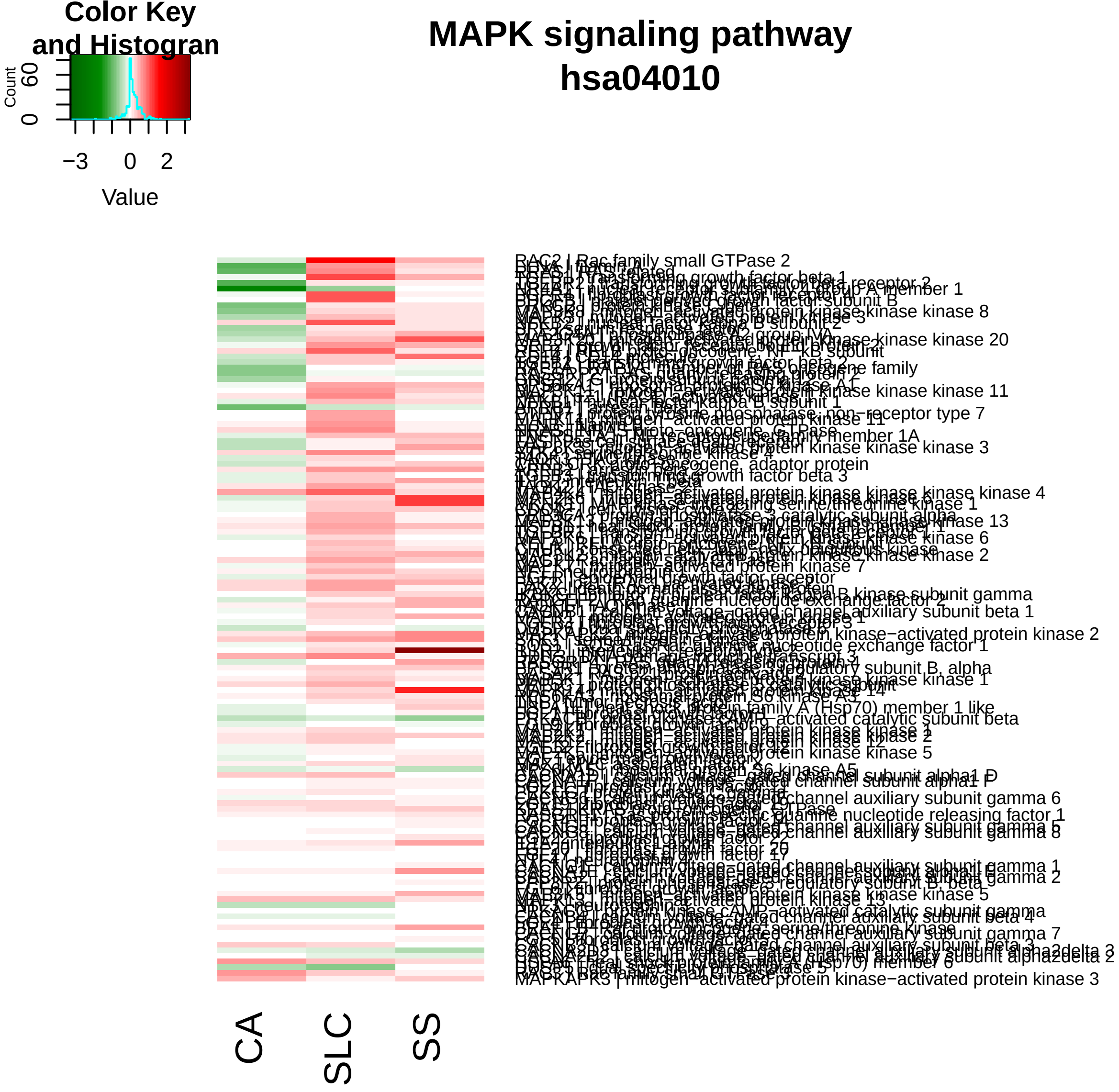

Signal transduction

ErbB signaling pathway
hsa04012

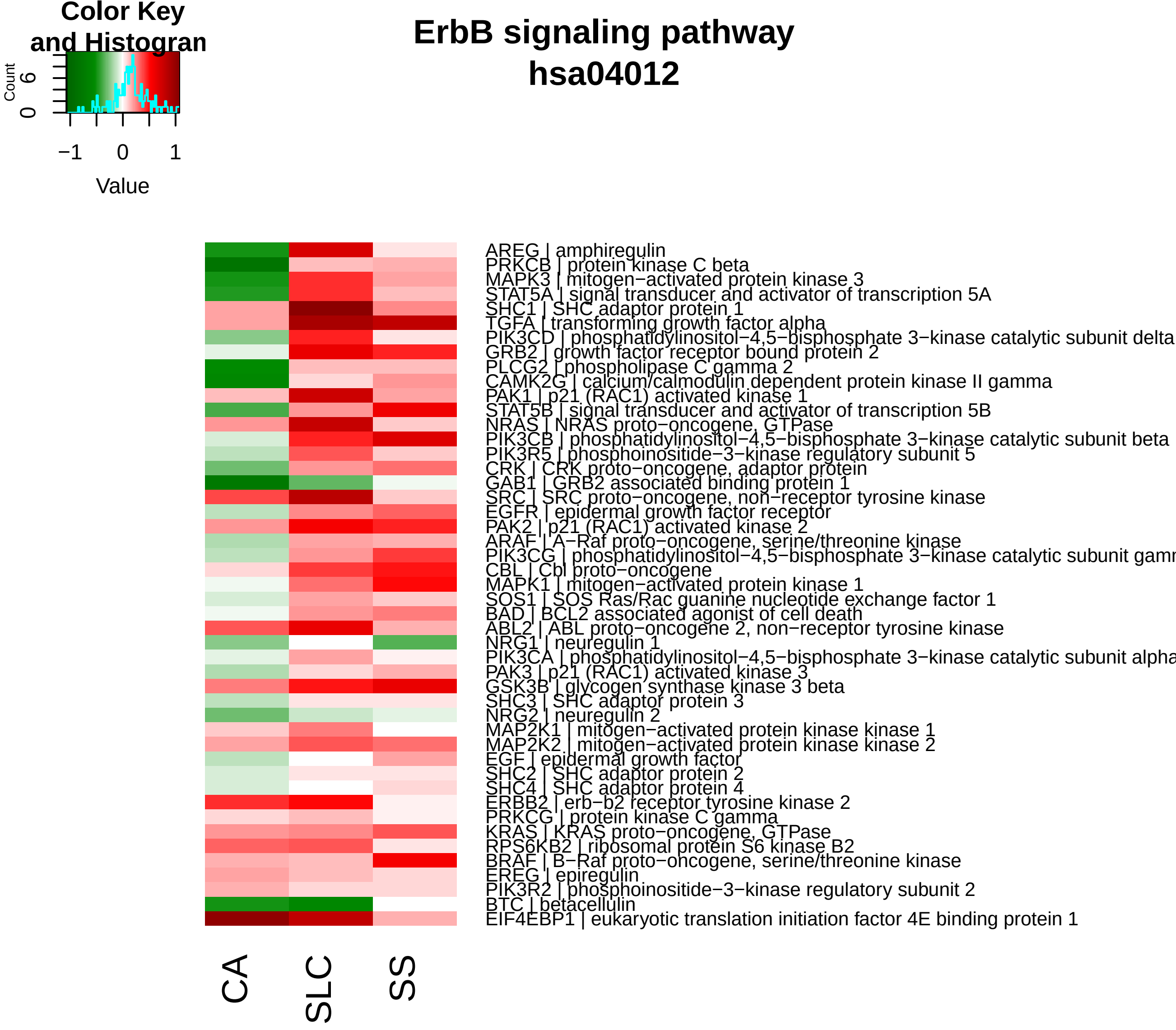

Signal transduction

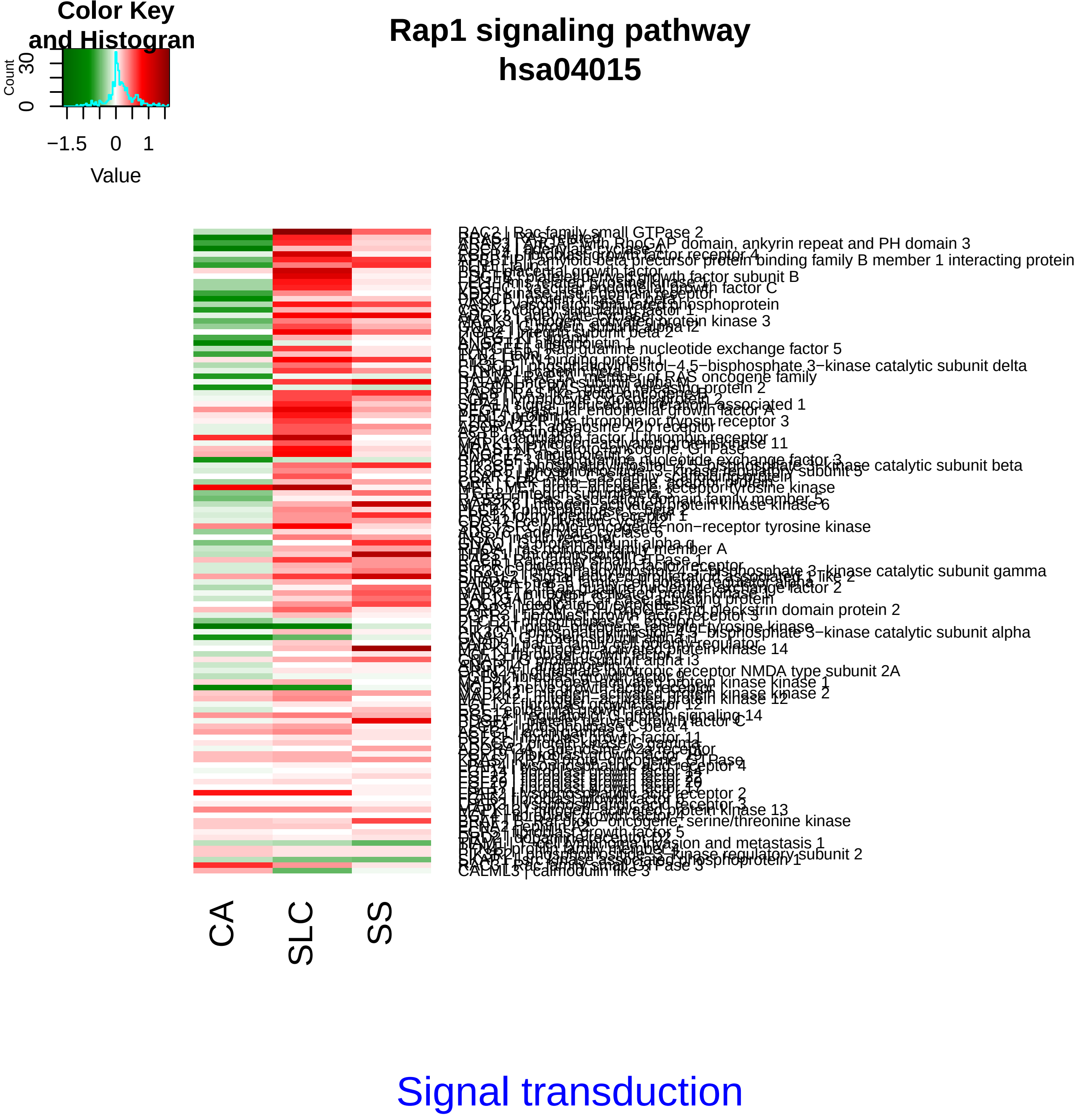

Color Key and Histogram
Count
0
30
−1.5
0
1
Value
Rap1 signaling pathway
hsa04015
CA
SLC
SS
Signal transduction

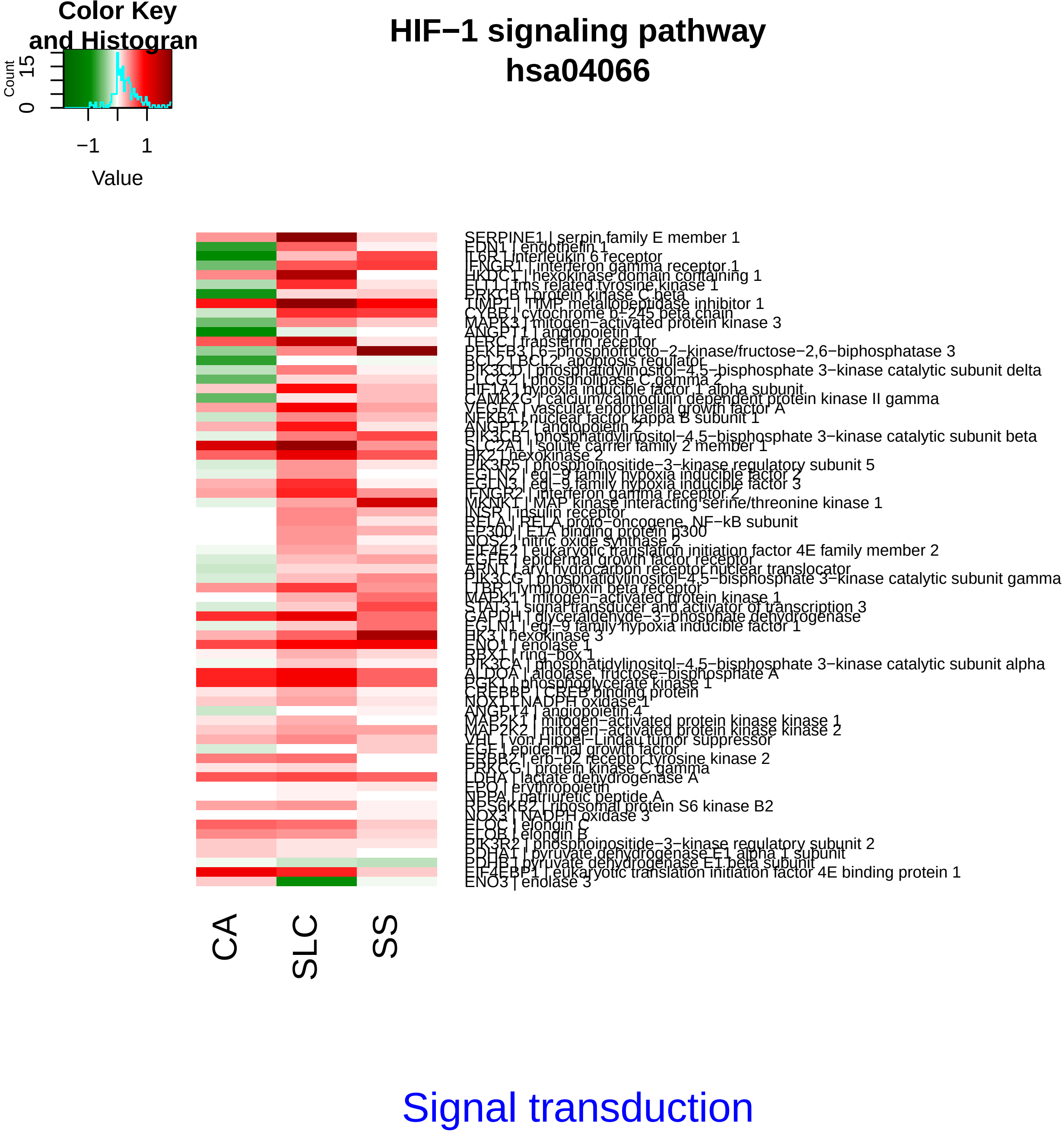
HIF−1 signaling pathway
hsa04066
Color Key
and Histogran
Count
0
15
−1
1
Value
SERPINE1 | serpin family E member 1
EDN1 | endothelin 1
IL6R | interleukin 6 receptor
IFNGR1 | interferon gamma receptor 1
HKDC1 | hexokinase domain containing 1
FLT1 | fms related tyrosine kinase 1
PRKCB | protein kinase C beta
TIMP1 | TIMP metallopeptidase inhibitor 1
CYBB | cytochrome b−245 beta chain
MAPK3 | mitogen−activated protein kinase 3
ANGPT1 | angiopoietin 1
TFRC | transferrin receptor
PFKFB3 | 6−phosphofructo−2−kinase/fructose−2,6−biphosphatase 3
BCL2 | BCL2, apoptosis regulator
PIK3CD | phosphatidylinositol−4,5−bisphosphate 3−kinase catalytic subunit delta
PLCG2 | phospholipase C gamma 2
HIF1A | hypoxia inducible factor 1 alpha subunit
CAMK2G | calcium/calmodulin dependent protein kinase II gamma
VEGFA | vascular endothelial growth factor A
NFKB1 | nuclear factor kappa B subunit 1
ANGPT2 | angiopoietin 2
PIK3CB | phosphatidylinositol−4,5−bisphosphate 3−kinase catalytic subunit beta
SLC2A1 | solute carrier family 2 member 1
HK2 | hexokinase 2
PIK3R5 | phosphoinositide−3−kinase regulatory subunit 5
EGLN2 | egl−9 family hypoxia inducible factor 2
EGLN3 | egl−9 family hypoxia inducible factor 3
IFNGR2 | interferon gamma receptor 2
MKNK1 | MAP kinase interacting serine/threonine kinase 1
INSR | insulin receptor
RELA | RELA proto−oncogene, NF−kB subunit
EP300 | E1A binding protein p300
NOS2 | nitric oxide synthase 2
EIF4E2 | eukaryotic translation initiation factor 4E family member 2
EGFR | epidermal growth factor receptor
ARNT | aryl hydrocarbon receptor nuclear translocator
PIK3CG | phosphatidylinositol−4,5−bisphosphate 3−kinase catalytic subunit gamma
LTBR | lymphotoxin beta receptor
MAPK1 | mitogen−activated protein kinase 1
STAT3 | signal transducer and activator of transcription 3
GAPDH | glyceraldehyde−3−phosphate dehydrogenase
EGLN1 | egl−9 family hypoxia inducible factor 1
HK3 | hexokinase 3
ENO1 | enolase 1
RBX1 | ring−box 1
PIK3CA | phosphatidylinositol−4,5−bisphosphate 3−kinase catalytic subunit alpha
ALDOA | aldolase, fructose−bisphosphate A
PGK1 | phosphoglycerate kinase 1
CREBBP | CREB binding protein
NOX1 | NADPH oxidase 1
ANGPT4 | angiopoietin 4
MAP2K1 | mitogen−activated protein kinase kinase 1
MAP2K2 | mitogen−activated protein kinase kinase 2
VHL | von Hippel−Lindau tumor suppressor
EGF | epidermal growth factor
ERBB2 | erb−b2 receptor tyrosine kinase 2
PRKCG | protein kinase C gamma
LDHA | lactate dehydrogenase A
EPO | erythropoietin
NPPA | natriuretic peptide A
RPS6KB2 | ribosomal protein S6 kinase B2
NOX3 | NADPH oxidase 3
ELOC | elongin C
ELOB | elongin B
PIK3R2 | phosphoinositide−3−kinase regulatory subunit 2
PDHA1 | pyruvate dehydrogenase E1 alpha 1 subunit
PDHB | pyruvate dehydrogenase E1 beta subunit
EIF4EBP1 | eukaryotic translation initiation factor 4E binding protein 1
ENO3 | enolase 3
CA
SLC
SS
Signal transduction

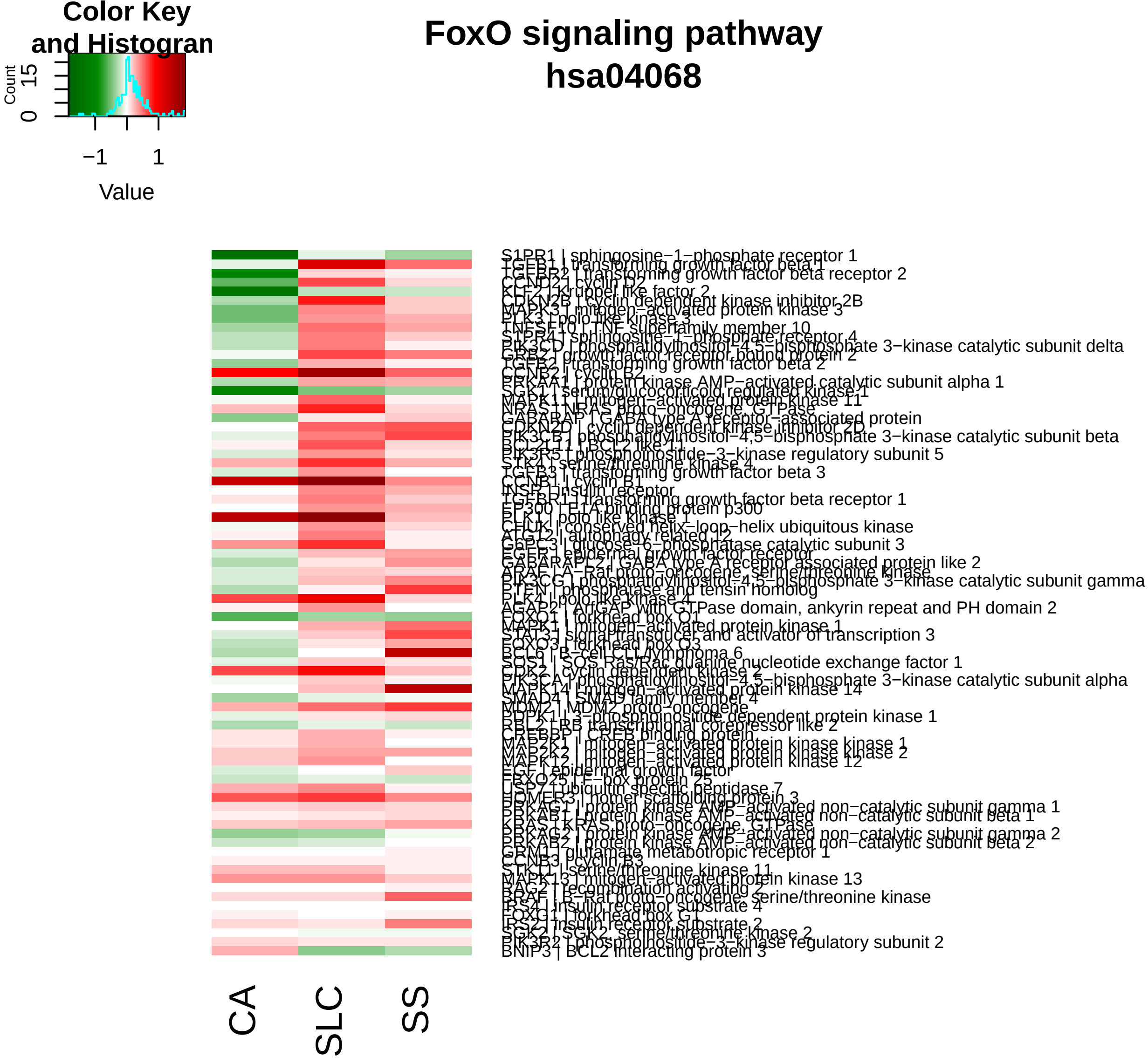

Signal transduction

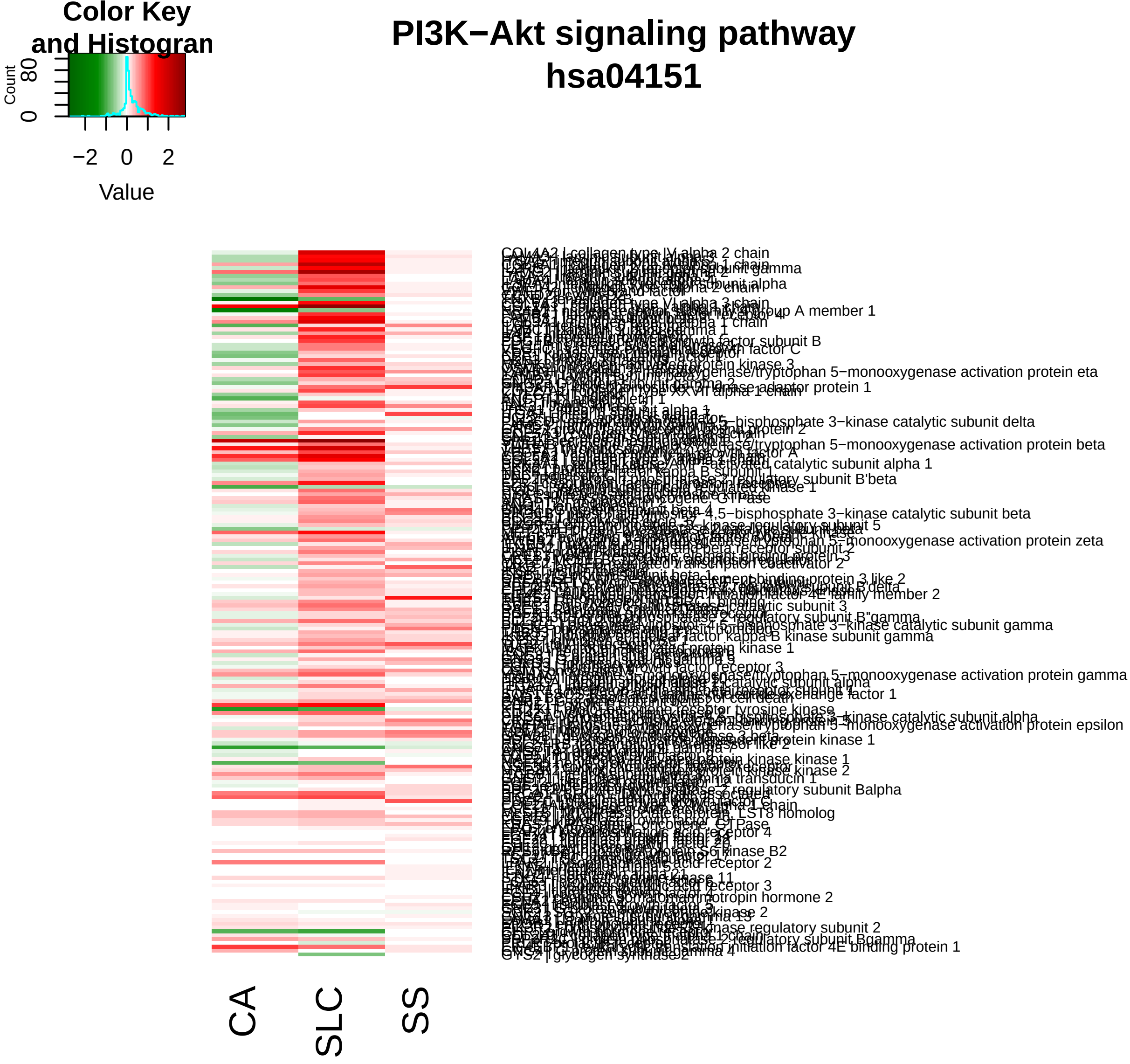

Signal transduction

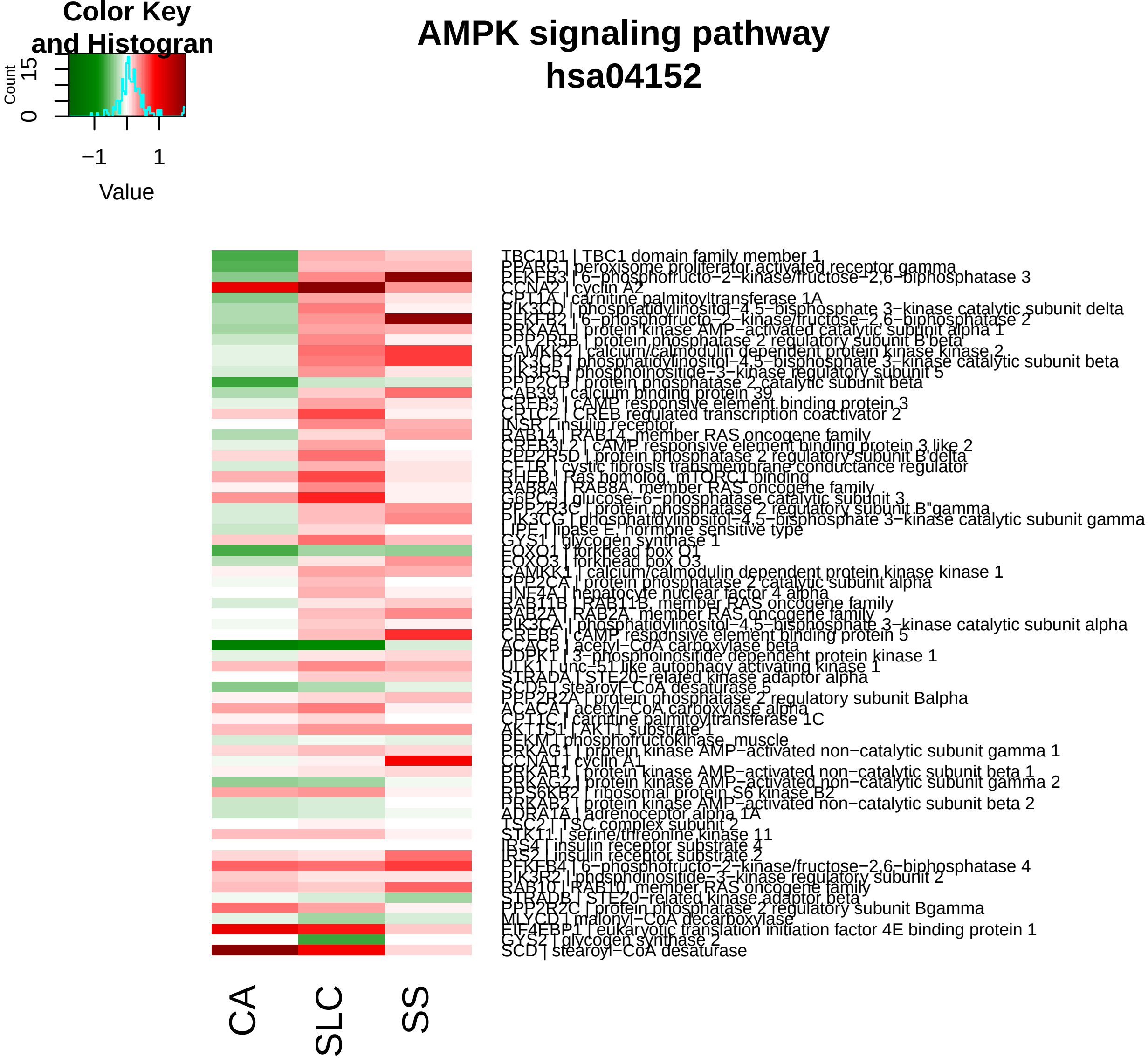

Signal transduction

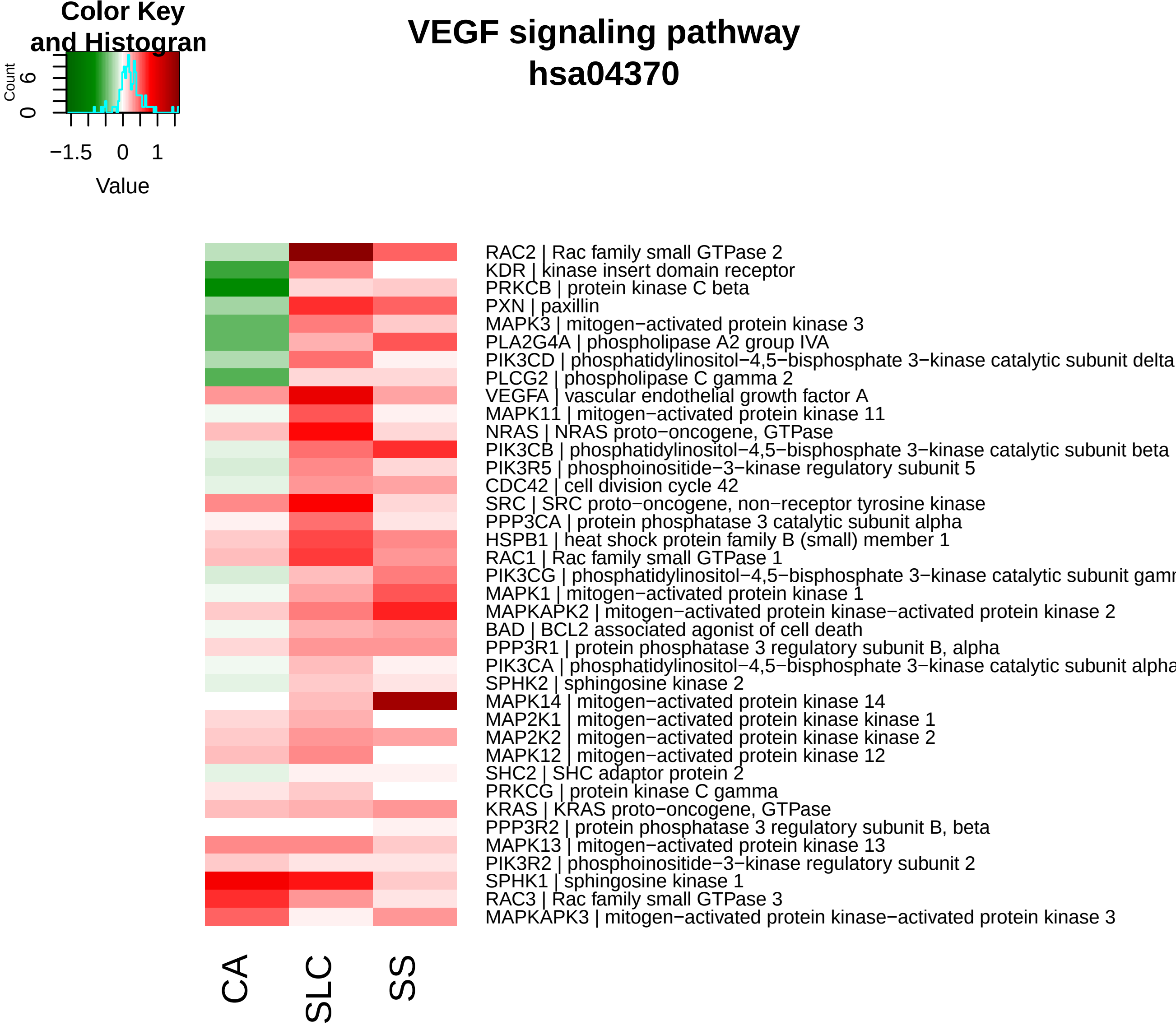

Signal transduction

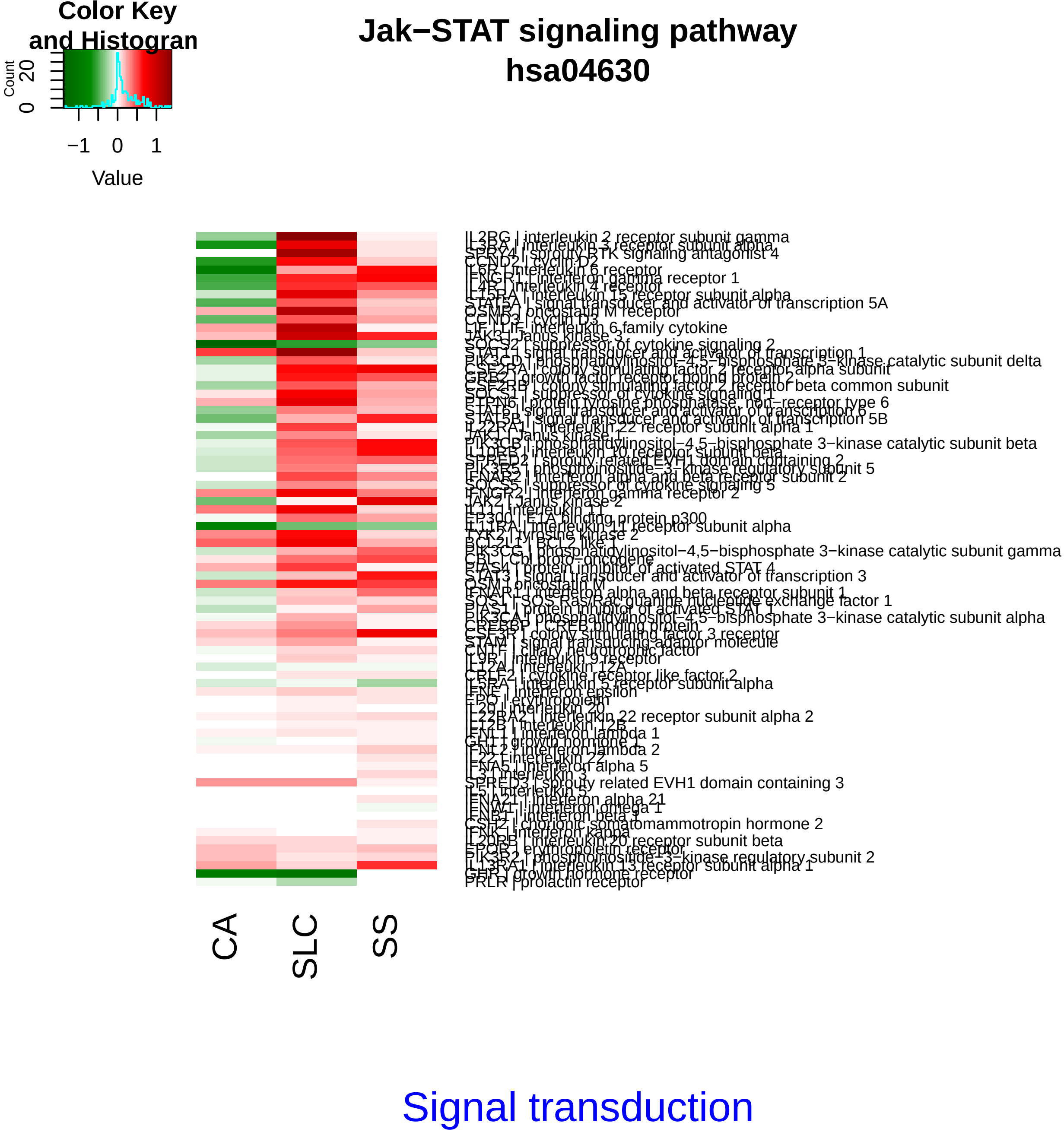
Color Key and Histogran
Count
0
20
−1
0
1
Value
Jak−STAT signaling pathway
hsa04630
IL2RG | interleukin 2 receptor subunit gamma
IL3RA | interleukin 3 receptor subunit alpha
SPRY4 | sprouty RTK signaling antagonist 4
CCND2 | cyclin D2
IL6R | interleukin 6 receptor
IFNGR1 | interferon gamma receptor 1
IL4R | interleukin 4 receptor
IL15RA | interleukin 15 receptor subunit alpha
STAT5A | signal transducer and activator of transcription 5A
OSMR | oncostatin M receptor
CCND3 | cyclin D3
LIF | LIF, interleukin 6 family cytokine
JAK3 | Janus kinase 3
SOCS2 | suppressor of cytokine signaling 2
STAT1 | signal transducer and activator of transcription 1
PIK3CD | phosphatidylinositol−4,5−bisphosphate 3−kinase catalytic subunit delta
CSF2RA | colony stimulating factor 2 receptor alpha subunit
GRB2 | growth factor receptor bound protein 2
CSF2RB | colony stimulating factor 2 receptor beta common subunit
SOCS1 | suppressor of cytokine signaling 1
PTPN6 | protein tyrosine phosphatase, non−receptor type 6
STAT6 | signal transducer and activator of transcription 6
STAT5B | signal transducer and activator of transcription 5B
IL22RA1 | interleukin 22 receptor subunit alpha 1
JAK1 | Janus kinase 1
PIK3CB | phosphatidylinositol−4,5−bisphosphate 3−kinase catalytic subunit beta
IL10RB | interleukin 10 receptor subunit beta
SPRED2 | sprouty related EVH1 domain containing 2
PIK3R5 | phosphoinositide−3−kinase regulatory subunit 5
IFNAR2 | interferon alpha and beta receptor subunit 2
SOCS5 | suppressor of cytokine signaling 5
IFNGR2 | interferon gamma receptor 2
JAK2 | Janus kinase 2
IL11 | interleukin 11
EP300 | E1A binding protein p300
IL11RA | interleukin 11 receptor subunit alpha
TYK2 | tyrosine kinase 2
BCL2L1 | BCL2 like 1
PIK3CG | phosphatidylinositol−4,5−bisphosphate 3−kinase catalytic subunit gamma
CBL | Cbl proto−oncogene
PIAS4 | protein inhibitor of activated STAT 4
STAT3 | signal transducer and activator of transcription 3
OSM | oncostatin M
IFNAR1 | interferon alpha and beta receptor subunit 1
SOS1 | SOS Ras/Rac guanine nucleotide exchange factor 1
PIAS1 | protein inhibitor of activated STAT 1
PIK3CA | phosphatidylinositol−4,5−bisphosphate 3−kinase catalytic subunit alpha
CREBBP | CREB binding protein
CSF3R | colony stimulating factor 3 receptor
STAM | signal transducing adaptor molecule
CNTF | ciliary neurotrophic factor
IL9R | interleukin 9 receptor
IL12A | interleukin 12A
CRLF2 | cytokine receptor like factor 2
IL5RA | interleukin 5 receptor subunit alpha
IFNE | interferon epsilon
EPO | erythropoietin
IL20 | interleukin 20
IL22RA2 | interleukin 22 receptor subunit alpha 2
IL12B | interleukin 12B
IFNL1 | interferon lambda 1
GH1 | growth hormone 1
IFNL2 | interferon lambda 2
IL22 | interleukin 22
IFNA5 | interferon alpha 5
IL3 | interleukin 3
SPRED3 | sprouty related EVH1 domain containing 3
IL5 | interleukin 5
IFNA21 | interferon alpha 21
IFNW1 | interferon omega 1
IFNB1 | interferon beta 1
CSH2 | chorionic somatomammotropin hormone 2
IFNK | interferon kappa
IL20RB | interleukin 20 receptor subunit beta
EPOR | erythropoietin receptor
PIK3R2 | phosphoinositide−3−kinase regulatory subunit 2
IL13RA1 | interleukin 13 receptor subunit alpha 1
GHR | growth hormone receptor
PRLR | prolactin receptor
CA
SLC
SS
Signal transduction

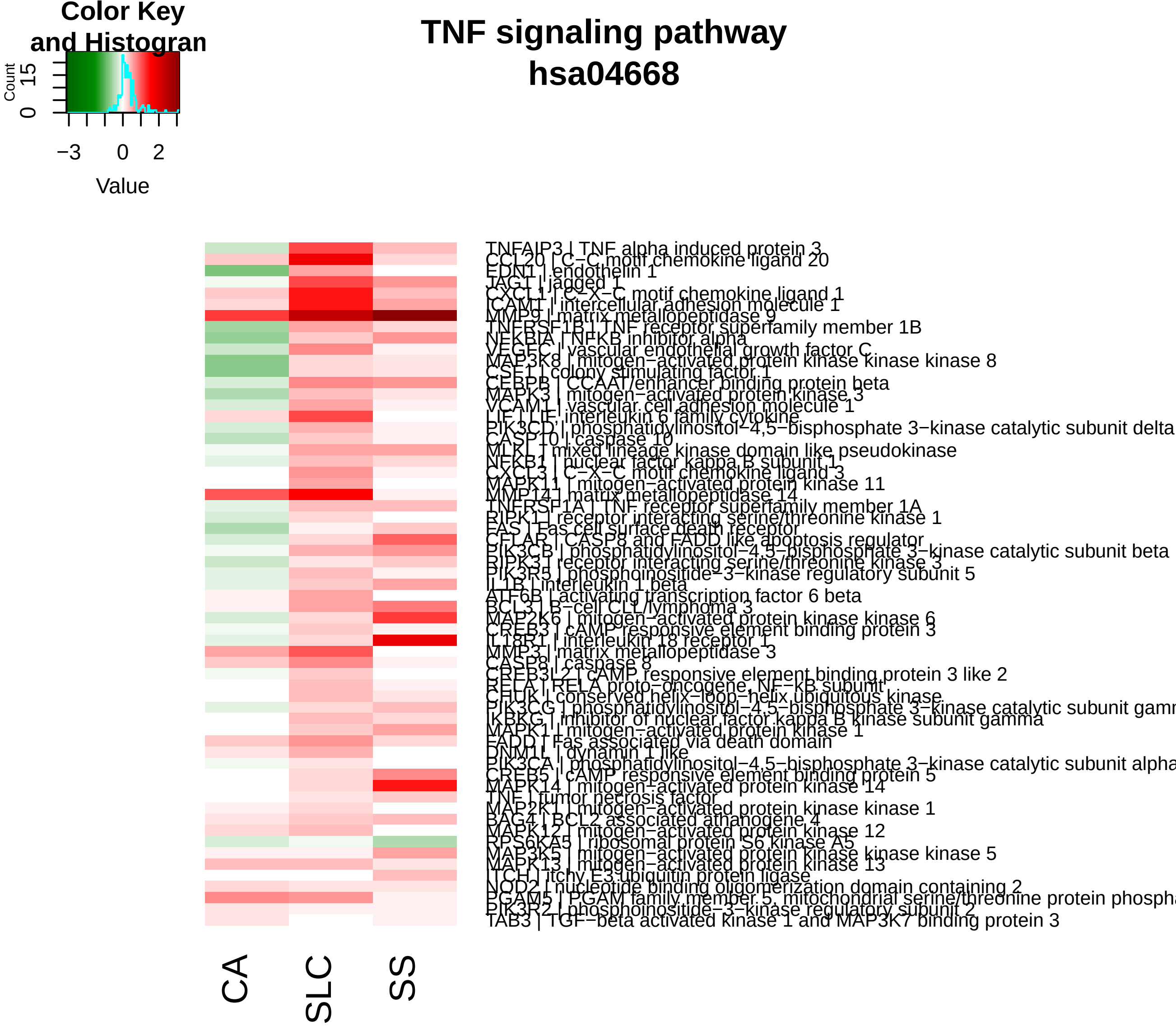

Signal transduction

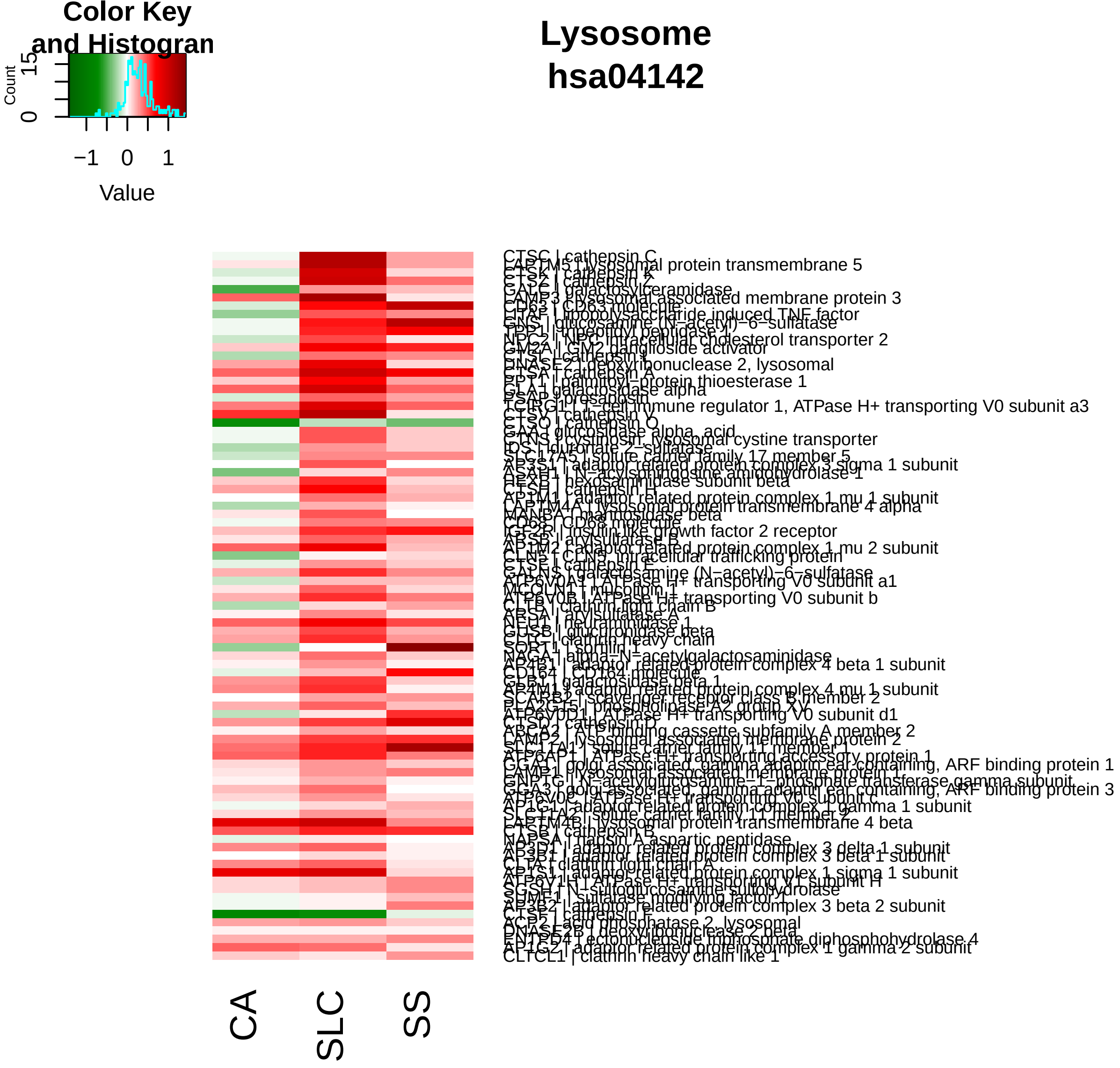

Transport and catabolism

Phagosome
hsa04145

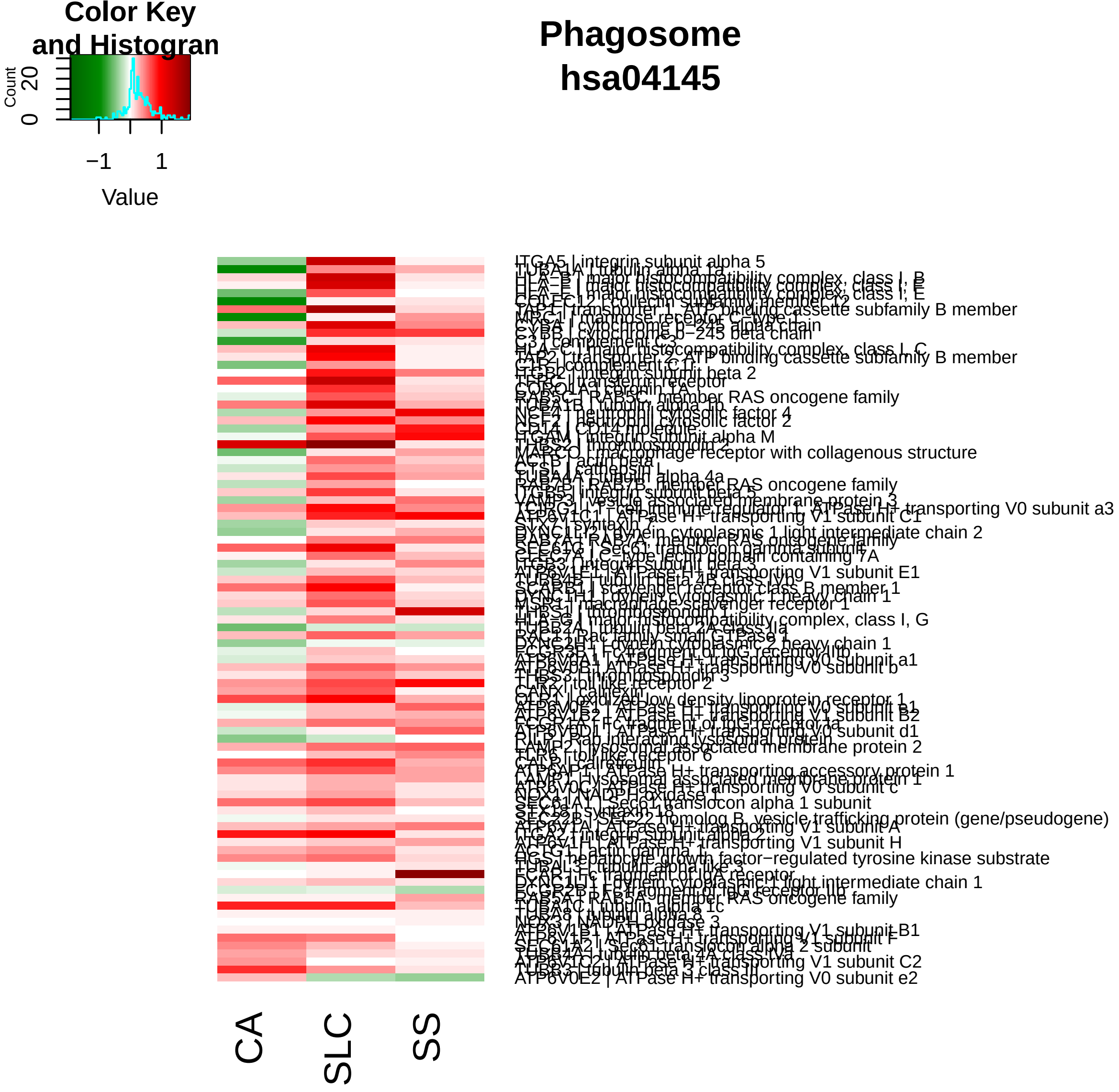

Transport and catabolism
