# Supplementary material for: Sepsis-associated pathways segregate cancer groups
Source: BMC Cancer. 2020 Apr 15;20:309. doi: 10.1186/s12885-020-06774-9 (PMC7160985; doi:10.1186/s12885-020-06774-9)
Supplement: Supplementary file 9 — Additional file 9: Text S1. [Functional classification of the pathways] The selected 66 pathways are divided into categories based on their functional relevance. The categories include catabolism, immune system, infection, cancer, signal transduction, ribosome biogenesis and carbohydrate metabolism. [file 12885_2020_6774_MOESM9_ESM.doc]

**Functional classification of the pathways**

The selected 66 pathways are divided into categories based on their functional relevance. The categories include catabolism, immune system, infection, cancer, signal transduction, ribosome biogenesis and carbohydrate metabolism. Many of these pathways are related to immune response to infection, such as, clot formation, proinflammatory effects, antiviral effects, chemotactic effects and T cell stimulation, degranulation, cytokine gene transcription and generation of eicosanoids, phagocytosis, and migration of leukocytes to the site of injury, and are consistently up-regulated in both SS and SLC group.

***Catabolism***

Two catabolic pathways namely, Lysosome and Phagosome, are observed to be mostly up-regulated in SS and SLC (Fig. 2, Additional File 4: Table S1). Upon network analysis, it is revealed that lysosome pathway has a very high value for betweenness centrality (680.2) (Additional File 5: Table S2), thus being essential to the structure of the network. Likewise, Phagosome, another catabolic pathway linked to lysosome, is uniformly up regulated in SS and SLC groups (Additional File 2: Figure S2).

Muscle wasting is one of the most common phenomena in both SS and cancer [1, 2]. Lysosome mediated protein degradation has been noted in substantial scale in such cases [1, 2]. Further, transcriptome based meta-analysis conducted by Ma et al. [3], reported up-regulation of lysosome pathway in sepsis while increased biogenesis of lysosomes has been observed in cancer cells [4]. In this study, we observe consistent up-regulation of catabolic pathways associated with lysosome and phagosome in SS and SLC, suggesting high catabolic rate in both the groups. Besides, lysosome is now considered a hub of metabolic signalling given its key role in maintaining cellular homeostasis [5]. It also appears as a central node in our network with high betweenness centrality.

***Immune system***

Both cancer and septic shock are associated with dysregulation in immune function. It is observed that immune system pathways associated with clot formation (Platelet activation), proinflammatory effects, antiviral effects, chemotactic effects and T cell stimulation (Toll-like receptor signalling pathway), degranulation, cytokine gene transcription and generation of eicosanoids (Fc epsilon RI signalling pathway), phagocytosis (Fc gamma R-mediated phagocytosis), and migration of leukocytes to the site of injury (Leukocyte transendothelial migration) are uniformly up regulated in both SS and SLC groups (Additional File 2: Figure S2).

***Infection***

Infection is a common theme in both septic shock (which originates as sepsis) and many cancers (known to be induced by microbial infection). Many infection-associated pathways, such as, Bacterial invasion of epithelial cells, *Vibrio cholerae* infection, Epithelial cell signalling in *Helicobacter pylori* infection, Salmonella infection, Pertussis, Legionellosis, Tuberculosis, Chagas disease (American trypanosomiasis), Amoebiasis, Hepatitis C and Hepatitis B are significantly upregulated in SS and SLC group (Additional File 2: Figure S2).

***Cancer pathways***

Many cancer-associated pathways, such as Transcriptional misregulation in cancer, Proteoglycans in cancer, Central carbon metabolism in cancer, Choline metabolism in cancer, Chemical carcinogenesis, Renal cell carcinoma, Pancreatic cancer, Glioma, Prostate cancer, Melanoma, Bladder cancer, Acute myeloid leukemia, Non-small cell lung cancer are up regulated in SS and SLC group (Additional File 2: Figure S2).

***Signal transduction***

Signal transduction pathways involved in cell proliferation, differentiation, adhesion, migration, survival, cell cycle progression, anti-apoptosis (MAPK, ErbB, Rap1, VEGF, JAK-STAT and PI3k-Akt signalling pathways) and cell cycle regulation, apoptosis, autophagy, oxidative stress response, DNA repair, immune regulation, muscle atrophy, growth arrest, leukocyte recruitment and activation, synthesis of inflammatory mediators, remodelling of extracellular matrix (HIF1, AMPK, FoxO and TNF signalling pathways) are uniformly up-regulated in SS group, and in most of the SLC group (Additional File 2: Figure S2).

***Endocrine system***

Five pathways associated with endocrine system viz., Insulin signalling, GnRH signalling, Progesterone-mediated oocyte maturation, Prolactin signalling pathway and Ovarian steroidogenesis are up-regulated in all SS groups as well as in SLC group (Additional File 2: Figure S2).

***Carbohydrate metabolism***

Carbohydrate metabolism pathways including Galactose metabolism, Glycolysis/Gluconeogenesis, and Pentose phosphate pathway are up-regulated in SS group and most of the SLC group cancers. In our study, Galactose metabolism pathway is up-regulated in SLC and SS groups. Glycolysis/Gluconeogenesis (a source of energy and metabolite precursors of other pathways) plays a central role in cell as reflected by highest betweenness centrality parameter value i.e. 1599.06 (Additional File 5: Table S2). Pentose phosphate pathway, which generates precursors for synthesis of purine, pyrimidine and histidine, is up-regulated across the SS group along with the LIHC, CHOL, ESCA and STAD of SLC group.

References:

1. Klaude M, Mori M, Tjäder I, Gustafsson T, Wernerman J, Rooyackers O. Protein metabolism and gene expression in skeletal muscle of critically ill patients with sepsis. Clinical science. 2012 Feb 1;122(3):133-42.
2. Penna F, Costamagna D, Pin F, Camperi A, Fanzani A, Chiarpotto EM, Cavallini G, Bonelli G, Baccino FM, Costelli P. Autophagic degradation contributes to muscle wasting in cancer cachexia. The American journal of pathology. 2013 Apr 1;182(4):1367-78.
3. Ma J, Chen C, Barth AS, Cheadle C, Guan X, Gao L. Lysosome and cytoskeleton pathways are robustly enriched in the blood of septic patients: a meta-analysis of transcriptomic data. Mediators of inflammation. 2015;<http://dx.doi.org/10.1155/2015/984825>.
4. Kallunki T, Olsen OD, Jäättelä M. Cancer-associated lysosomal changes: friends or foes?. Oncogene. 2013 Apr;32(16):1995.
5. Lamming DW, Bar‐Peled L. Lysosome: The metabolic signalling hub. Traffic. 2019 Jan;20(1):27-38.
